# Total chemical synthesis of glycocin F and analogues: *S*-glycosylation confers improved antimicrobial activity†
†Electronic supplementary information (ESI) available: Experimental details for the synthesis of all peptides, analytical LC and HR-MS, and circular dichroism data. See DOI: 10.1039/c7sc04383j


**DOI:** 10.1039/c7sc04383j

**Published:** 2018-01-12

**Authors:** Zaid Amso, Sean W. Bisset, Sung-Hyun Yang, Paul W. R. Harris, Tom H. Wright, Claudio D. Navo, Mark L. Patchett, Gillian E. Norris, Margaret A. Brimble

**Affiliations:** a School of Chemical Sciences , The University of Auckland , 23 Symonds St , Auckland 1142 , New Zealand . Email: m.brimble@auckland.ac.nz ; Fax: +64 9 3737422 ; Tel: +64 9 3737599; b Institute of Fundamental Sciences , Massey University , Colombo Rd , Palmerston North 4442 , New Zealand; c Maurice Wilkins Centre for Molecular Biodiscovery , The University of Auckland , Private Bag 92019 , Auckland 1142 , New Zealand; d School of Biological Sciences , The University of Auckland , 3 Symonds St , Auckland 1142 , New Zealand; e Dept. Química , Universidad de La Rioja , Centro de Investigación en Síntesis Química , E-26006 Logroño , Spain

## Abstract

Glycocin F (GccF) is a unique diglycosylated bacteriocin peptide that possesses potent and reversible bacteriostatic activity against a range of Gram-positive bacteria. GccF is a rare example of a ‘glycoactive’ bacteriocin, with both the *O*-linked *N*-acetylglucosamine (GlcNAc) and the unusual *S*-linked GlcNAc moiety important for antibacterial activity. In this report, glycocin F was successfully prepared using a native chemical ligation strategy and folded into its native structure. The chemically synthesised glycocin appeared to be slightly more active than the recombinant material produced from *Lactobacillus plantarum*. A second-generation synthetic strategy was used to prepare 2 site selective ‘glyco-mutants’ containing either two *S*-linked or two *O*-linked GlcNAc moieties; these mutants were used to probe the contribution of each type of glycosidic linkage to bacteriostatic activity. Replacing the *S*-linked GlcNAc at residue 43 with an *O*-linked GlcNAc decreased the antibacterial activity, while replacing *O*-linked GlcNAc at position 18 with an *S*-linked GlcNAc increased the bioactivity suggesting that the *S*-glycosidic linkage may offer a biologically-inspired route towards more active bacteriocins.

## Abstract 2

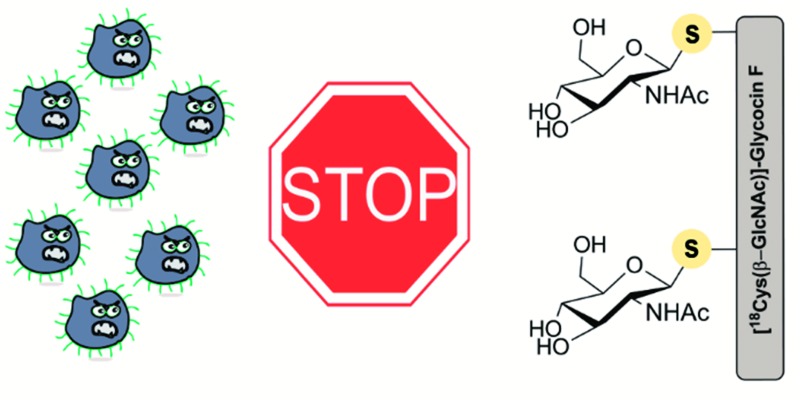
Replacing the *O*-linked saccharide in the bacteriocin glycocin F with an *S*-linked version results in a peptidomimetic that increases the bacteriostatic effect.

## Introduction

Microorganisms produce a highly diverse array of compounds that can be harnessed as anti-bacterial agents.^1^ Bacteriocins are ribosomally synthesized and post-translationally modified peptides (RiPPs) secreted by bacteria as a primary defence mechanism against competing bacteria.^2^ Bacteriocins from lactic acid bacteria have received considerable attention due to a desirable safety profile, stability across wide pH and temperature ranges, antimicrobial activity against various pathogens, and no cross-resistance with antibiotics.^3^–^5^ These features have prompted interest in bacteriocins as novel antimicrobial agents, probiotics and natural preservatives.^4^

Glycocin F (GccF, **1**) is a potent bacteriocin originally isolated from liquid culture of *Lactobacillus plantarum* KW30.^6^ GccF is a 43-residue helical peptide that contains two interlocked disulfide bonds (^5^Cys–^28^Cys and ^12^Cys–^21^Cys) and two β-linked *N*-acetyl-d-glucosamine (GlcNAc) moieties, connected to the side chain of ^18^Ser *via* the oxygen atom and ^43^Cys *via* the sulfur atom.^7^ The glycosylated cysteine, in particular, is an extremely rare post-translational modification in bacteria and has only been found in two other glycopeptides to date (sublancin and thurandacin).^8^–^10^ GccF exhibits bacteriostatic activity against a relatively wide range of Gram positive bacteria, with *L. plantarum* strains suspected to be its natural target.^6^,^7^ Notably, ASM1, a close homologue of GccF, and enterocin 96 are the only glycosylated bacteriocins which have been shown to be “glycoactive”,^11^–^13^ that is, the saccharide moieties are essential for biological activity. In a previous study, removal of the GlcNAc attached to ^18^Ser completely abolished the activity, while removal of the *C*-terminal fragment ^42^His–^43^Cys(β-GlcNAc) reduced the activity 44-fold.^7^ The *C*-terminal *S*-glycosidic linkage is of particular interest, as the natural functions of such bonds remain unclear. Further, the improved biochemical stability of *S*-glycoside linkages^14^ compared to their *O*-linked congeners provides considerable scope for the development of *S*-glycosylated bacteriocins as both preservatives and therapeutics.

Since the discovery of GccF (**1**), attempts to dissect its molecular mechanism and cellular targets have been hampered by the inefficiency of its isolation from cultures of *L. plantarum*. Chemical synthesis of complex bacteriocin glycopeptides is an attractive alternative to direct isolation or recombinant production as it offers atomic-level control over peptide sequence and modifications. However, few total syntheses of glycosylated bacteriocins have been reported to date. An example is the synthesis of 37-residue sublancin 168 by Payne *et al.*^9^*via* solid-phase peptide synthesis coupled with a convergent ligation methodology. Two other glycosylated bacteriocins thurandacin and enterocin 96 have been prepared using chemoenzymatic methods.^8^,^13^

We have previously reported^11^ the first synthesis of a biologically active form of GccF, identical in structure to the natural product except for *C*-terminal amidation. The synthetic *C*-terminal amide analogue of GccF exhibited a reduced bacteriostatic activity compared to the natural peptide derived from *L. plantarum* culture.^11^ We therefore sought to obtain the native GccF that contains the *C*-terminal acid to determine the role of the *C*-terminal carboxylate. We then sought to extend this further by chemically synthesizing ‘glyco-mutants’ of GccF to examine the chemical linkage between the peptide backbone and the GlcNAc. Our hypothesis is that replacing *O*-linked sugars with *S*-linked sugars would not greatly perturb the structural requirements of GccF, thereby preserving the bioactivity but may also result in enhanced enzymatic stability.^14^

Herein, the native form of glycocin F (**1**) was prepared and improvements on its chemical synthesis are reported. Notably, the synthetic GccF is equipotent to the isolated material in a liquid culture assay. Furthermore, several ‘glyco-mutants’ of GccF were generated, in which both of the two native glycosylation sites (^18^Ser and ^43^Cys) were substituted with *S*-linked β-GlcNAc (Cys(β-GlcNAc)) or *O*-linked β-GlcNAc (Ser(β-GlcNAc)) residues. These analogues have enabled us to better understand the contributions of each sugar linkage to the bacteriostatic activity of glycocin F and reveal the considerable potential of *S*-linked glycoconjugates as antimicrobial agents.

## Results and discussion

### Synthesis of glycocin F (**1**)

**Scheme 1:**
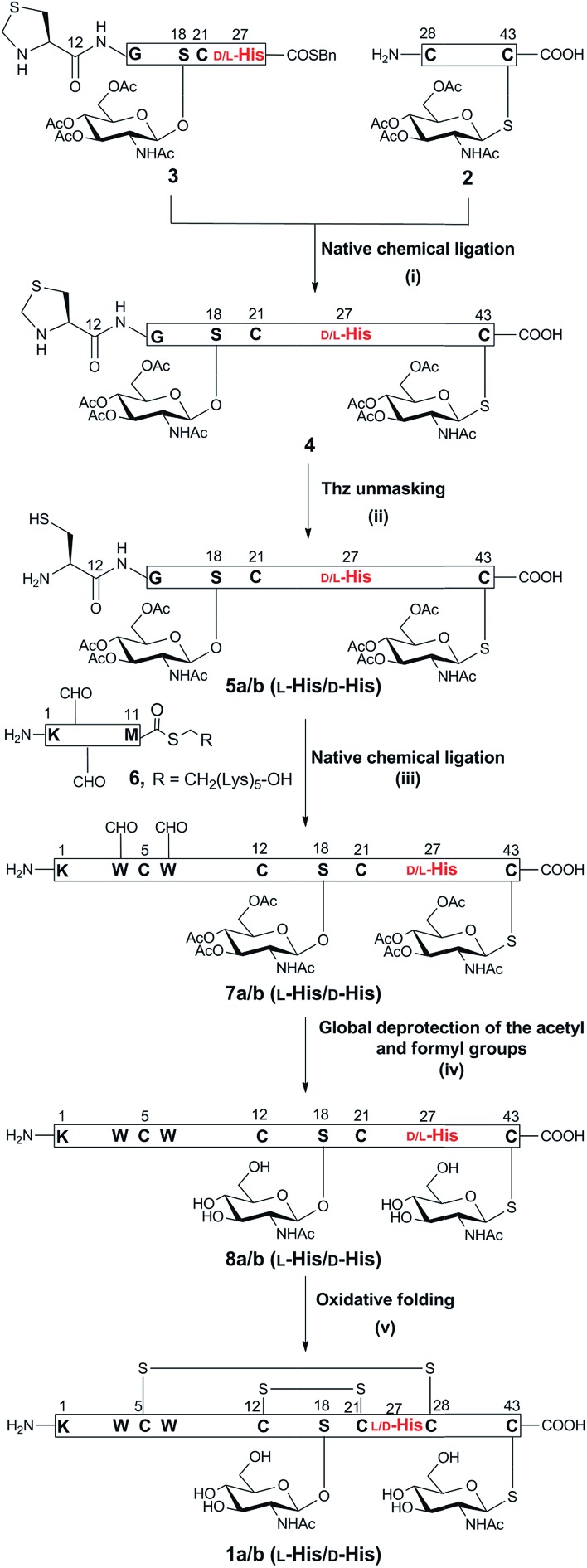
Synthesis of GccF (**1**) and [^27^d-His]-GccF, with the problematic His highlighted in red. Reagents and conditions: (i) 6 M Gn·HCl, 0.2 M Na_2_HPO_4_, 100 mM MPAA, 20 mM TCEP, pH 6.8, r.t, 2 h; (ii) 6 M Gn·HCl, 0.2 M Na_2_HPO_4_, methoxylamine·HCl, pH 4, r.t, 16 h; (iii) 6 M Gn·HCl, 0.2 M Na_2_HPO_4_, 100 mM MPAA, 20 mM TCEP, pH 6.8, r.t, 4 h; (iv) 6 M Gn·HCl, 1 M HEPES, hydrazine, 2-mercaptoethanol, 0 °C, 30 min; (v) 1.5 M Gn·HCl, 50 mM Na_2_HPO_4_, 2 mM cysteine, 0.25 mM cystine, 0.025 mM EDTA, 0.25 mM peptide, pH 8.2, 4 °C, 16 h.

The synthetic strategy to prepare native glycocin F (**1**) was analogous to that described for the previous synthesis of *C*-terminally amidated glycocin F.^11^ In that report, we adopted a three fragment native chemical ligation (NCL) strategy as depicted in Scheme 1. In the present work we employed the 2-chlorotrityl linker for the preparation of the racemisation-prone *C*-terminal acid fragment **2**, bearing a *C*-terminal cysteine (ESI Fig. 14 and Scheme 5†). The use of benzyl alcohol-based linkers was avoided as the esterification conditions for loading a cysteine residue to such linkers can lead to Cα racemisation and/or dehydroalanine by-product formation.^15^ 2-Chlorotrityl based resins does not involve carboxylate activation and thus does not result in racemisation. In our previous synthesis of glycocin F-amide, the 4-(4-hydroxymethyl-3-methoxyphenoxy)-butyric acid (HMPB) linker was used to prepare fragment **3**, causing substantial racemisation at ^27^His; pleasingly changing the linker to 2-chlorotrityl linker reduced this to *ca.* 20%.

The necessary *O*-glycosylated amino acid Fmoc-Ser(β-GlcNAc(OAc)_3_)-OH (Fmoc = 9-fluorenylmethoxycarbonyl) and *S*-glycosylated amino acid Fmoc-Cys(β-GlcNAc(OAc)_3_)-OH building blocks were prepared as previously described (ESI Scheme 1†). Native chemical ligation was undertaken as before and the fully assembled linear peptide was folded without incident (ESI Fig. 23 and 24†). Purification by HPLC yielded epimeric glycocin F (**1a**) and glycocin F (**1b**) in 29% and 32% yield, respectively.

The presence of the two disulfide bonds in **1b** was confirmed by HRESI-MS analysis (HR-ESIMS, *m*/*z* [M + 4H]^4+^; calculated for C_226_H_311_N_57_O_72_S_7_: 1041.4634, observed: 1040.8167) (ESI Fig. 26†). Glycocin F glycopeptides **1a** and **1b**, epimeric at ^27^His, were then compared with an authentic sample of glycocin F isolated from *L. plantarum* (ESI Fig. 25†) and assignment of the natural product was achieved by direct comparison of the HPLC retention time. Epimer **1b** eluted at an identical retention time to the isolated material and therefore was assigned the l-configuration at ^27^His. In contrast, epimer **1a** eluted earlier and was therefore assigned the d-configuration.

The secondary structure of synthetic folded GccF was determined by circular dichroism (CD) spectroscopy. Similar to the isolated GccF, the synthetic derivative **1b** and epimeric analogue **1a** exhibit the features expected for α-helical proteins, namely standard double negative ellipticity maxima at 210 and 221 nm, and a positive maximum near 194 nm (ESI Fig. 43†).

### Second-generation synthetic strategy provides expedient access to ‘glyco-mutants’ of glycocin F

#### A. Development of a two-fragment, single ligation strategy

**Scheme 2:**
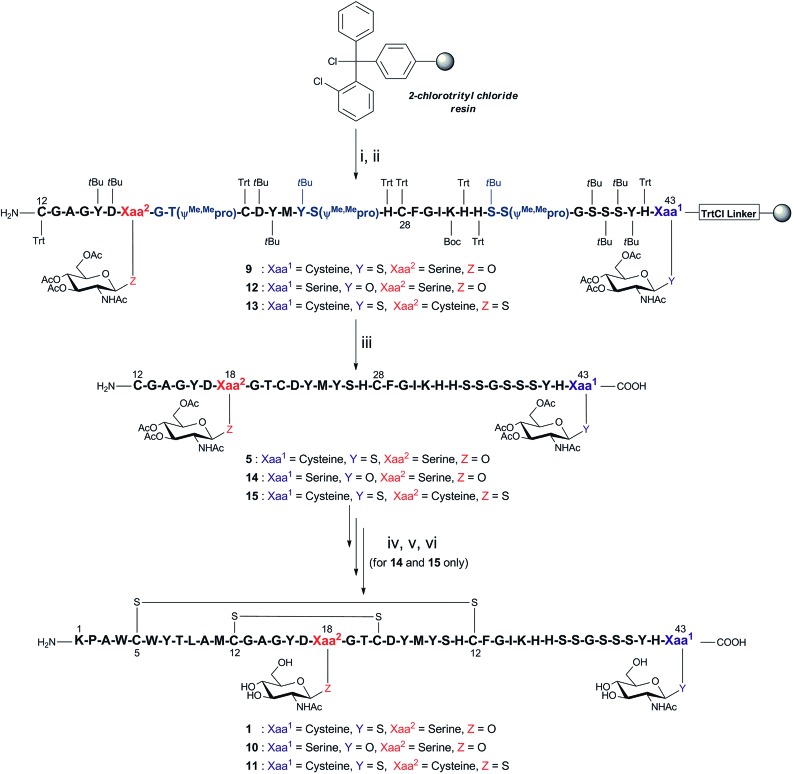
Optimised synthesis of GccF fragment **9**, which was then employed for the synthesis of glycol-mutants **10** and **11**. Reagents and conditions: (i) Fmoc-l-Cys(β-GlcNAc(OAc)_3_)-OH for **9** and **13** or Fmoc-l-Ser(β-GlcNAc(OAc)_3_)-OH for **12**, *i*Pr_2_EtN, CH_2_Cl_2_, r.t, 1 h; (ii) Fmoc-SPPS (Fmoc deprotection: 20% piperidine in DMF (v/v)), r.t, (2 × 5 min); coupling: Fmoc-amino acid, HATU, *i*Pr_2_EtN, DMF, r.t, 40 min except Fmoc-^18^Ser(β-GlcNAc(OAc)_3_)-OH, HATU, HOAt, TMP, DMF, r.t, overnight and Fmoc-^21^Cys(Trt)-OH, HATU, HOAt, TMP, CH_2_Cl_2_: DMF (1 : 1, v/v), r.t, 2 × 1 h; (iii) 94% TFA, 2.5% EDT, 2.5% H_2_O, 1% *i*Pr_3_SiH (v/v/v/v), r.t, 2 h; (iv)–(vi) for **14** and **15**, (iv) 6 M Gn·HCl, 0.2 M Na_2_HPO_4_, 100 mM MPAA, 20 mM TCEP, pH 6.8, r.t, 4 h; (v) 6 M Gn·HCl, 1 M HEPES, hydrazine, 2-mercaptoethanol, 0 °C, 30 min; (vi) 1.5 M Gn·HCl, 50 mM Na_2_HPO_4_, 2 mM cysteine, 0.25 mM cystine, 0.025 mM EDTA, 0.25 mM peptide, pH 8.2, 4 °C, 16 h.

In order to systematically examine structure–activity relationships of complex glycopeptides such as GccF (**1**), a large number of mutations must be made to enable dissection of each residue's contribution to activity. We therefore sought to develop a more efficient synthesis of GccF and analogues that would enable rapid production of a library of such mutants, at a scale sufficient for biochemical characterisation. The above-described strategy to prepare GccF involved synthesis of three polypeptide fragments that underwent two consecutive NCL reactions to produce full-length GccF. An alternative strategy was devised (Scheme 2) using only two fragments, consisting of a 32-amino acid digylcosylated fragment **5**, [^12^Cys–^18^Ser(β-GlcNAc(OAc)_3_)–^43^Cys(β-GlcNAc(OAc)_3_)] and the *N*-terminal fragment **6**, [^1^Lys–^11^Met-COSCH_2_CH_2_–(Lys)_5_]. While the previous synthesis of fragment **5** had required the ligation of the two short peptides **2** and **3** followed by ^12^Thz deprotection, we anticipated that **5** could be accessed through direct Fmoc-SPPS with higher yield. Following this approach, the native residue ^12^Cys could be used as the ligation site between **5** and **6**, thereby avoiding introduction of a non-native thiazolidine that would require further chemical manipulation after the ligation step. Critically, a single-ligation strategy would by-pass the problematic thioesterification step at ^27^His, avoiding racemisation at this residue.

Direct synthesis of fragment **5** was initiated on 2-ClTrtCl-polystyrene resin (Scheme 2), and subsequent peptide elongation was performed *via* Fmoc-SPPS using HATU/*i*Pr_2_EtN as the coupling reagent and 20% piperidine/DMF (v/v) as the Fmoc deblocking reagent. As peptide aggregation and thus reduced coupling yields had hindered previous efforts to synthesise this long fragment, three pseudoproline dipeptides^16^,^17^ were incorporated within the peptide chain, namely ^19^Gly–^20^Thr, ^25^Tyr–^26^Ser, and ^35^Ser–^36^Ser. Previous analysis of a truncated fragment ^16^Gly–^27^His, built on 2-ClTrtCl-based resin, revealed the presence of a significant, contaminating by-product (25% relative to expected peptide) with the same mass, which was attributed to ^21^Cys racemisation (ESI Fig. 27†). In order to prevent this, the coupling of ^21^Cys was achieved using HATU and 2,4,6-trimethylpyridine (TMP) in the presence of 1-hydroxy-7-azabenzotriazole (HOAt) in CH_2_Cl_2_/DMF (v/v; 1 : 1), conditions known to suppress racemisation.^18^,^19^ Following Fmoc-SPPS, the fully assembled peptide **9** was recovered from the resin with simultaneous removal of side chain protecting groups using the cleavage mixture TFA/EDT/H_2_O/*i*Pr_3_SiH (v/v/v/v; 94 : 2.5 : 2.5 : 1). Under the above conditions, the desired glycopeptide **5** was successfully obtained in 30% crude yield and >73% purity, with successful inhibition of ^21^Cys racemisation and avoiding ^27^His racemisation (ESI Fig. 28†).

#### B. Generation of ‘glyco-mutants’ of glycocin F

As glycocin F (**1**) was already synthesised, its preparation from glycopeptide **5** using the optimised synthetic strategy was not undertaken. Instead, we employed the improved methodology for the synthesis of ‘glyco-mutant’ analogues of glycocin F. Thus, two analogues of glycocin F (peptides **10** and **11**) were prepared, each bearing a single modification at one of the glycoside positions. Thus, analogue **10** was designed to contain *O*-linked GlcNAc moieties at amino acid positions 18 and 43. To prepare this analogue, the *C*-terminal ^43^Cys(β-GlcNAc) was replaced with a Ser(β-GlcNAc). Analogue **11** was designed to have *S*-linked GlcNAc moieties at positions 18 and 43. To achieve this, the internal ^18^Ser(β-GlcNAc) was replaced with a Cys(β-GlcNAc). Both analogues were designed to elucidate the biological rationale for incorporation of an *S*-glycosidic linkage in native glycocin F. The requisite resin-bound fragments **12** and **13** containing the desired glycosides, were synthesised in an analogous fashion to that described above for peptide **9** (Scheme 2 and ESI Fig. 29 and 30†). Polypeptides **14** and **15** were then individually ligated with the N-terminal fragment, peptide thioester ^1^Lys–^11^Met-COSCH_2_CH_2_–(Lys)_5_**6**. NCL reactions were performed as described previously (6 M Gn·HCl, 0.2 M Na_2_HPO_4_, 20 mM TCEP, and 100 mM MPAA, pH 6.8) at a final peptide concentration of 1 mM, and provided the ligation products in good yields (40% from **12**, and 44% from **13**) (ESI Schemes 31 and 33†). The ligation products were subjected to simultaneous formyl and acetyl deprotection, purified by RP-HPLC and subjected to oxidative folding, following the procedures developed above for the preparation of GccF **1**. Each ‘glyco-mutant’ of GccF folded successfully to afford a single folded species, as determined by HPLC, within 16 h (ESI Fig. 39 and 41†). The presence of two disulfide bonds in each analogue was confirmed by high resolution mass spectrometry (HRMS) with an observed mass for glyco-mutants **10** and **11**, of 1037.6213 ([M + 5H]^5+^) and 1044.0121 ([M + 5H]^5+^) Da, respectively, consistent with the calculated masses of the desired products (ESI Fig. 40 and 42†).

The secondary structure of glyco-mutants **10** and **11** was determined by circular dichroism (CD) spectroscopy, which confirmed a similar α-helical structure to the native glycosin F bacteriocin (ESI Fig. 43†).

### Biological evaluation of synthetic GccF (**1b**) and [^27^d-His]-GccF and “glyco-mutants”

Recent research from one of our laboratories (Norris) implicates a GlcNAc-specific phosphotransferase system (PTS) transporter in the cell membranes of susceptible Gram-positive bacteria as the likely target of glycocin F. It is hypothesised that glycocin F binds to the transmembrane domain of its receptor through the tethered GlcNAc residues and disrupts sugar-processing and regulatory activities essential for bacterial growth. Although the exact details remain unknown, a full understanding of the mechanism of action of glycocin F will afford a unique opportunity to develop an entirely new suite of PTS-targeted glycoconjugate antimicrobials for therapeutic and industrial application.

To evaluate the antibacterial activity of synthetic glycocin F (**1b**), [^27^d-His]-GccF (**1a**) and glyco-mutants **10** and **11**, the IC_50_ values against *Lactobacillus plantarum* ATCC 8014 were measured using a liquid culture assay. Synthetic GccF (**1b**) was used as the positive control (Table 1).

**Table 1:** Activity of bacterially isolated GccF, the synthesised GccF peptides and GccF glyco-mutants

Compound #	Modification	IC_50_
**Native**	Isolated GccF	2.0 ± 0.20 nM
**1b**	Synthetic GccF	1.13 ± 0.20 nM
**1a**	[^27^d-His]-GccF	0.98 ± 0.17 nM
**10**	^43^Cys(GlcNAc) to Ser(GlcNAc)	12.1 ± 0.20 nM
**11**	^18^Ser(GlcNAc) to Cys(GlcNAc)	0.60 ± 0.10 nM

The IC_50_ of the bacterially produced glycocin F was found to be 2.0 ± 0.20 nM. The synthetic glycocin F (**1b**) exhibited an enhanced antibacterial activity relative to this batch of isolated material (IC_50_ = 1.13 ± 0.20 nM). Considering that the previously reported GccF-NH_2_ exhibited reduced bacteriostatic activity (IC_50_ = 1.6 nM),^11^ this current result provides insight into the relationship of the target glycopeptide with its target receptor: amidation of the *C*-terminus was in fact responsible for the reduced effect, implying that a positively charged residue within the receptor may be interacting with the negatively charged carboxylate *C*-terminus.

The importance of the *C*-terminal carboxylate for bioactivity was also verified from the IC_50_ value of [^27^d-His]-GccF (**1a**) (0.98 ± 0.17 nM). Interestingly, the previously reported [^27^d-His]-GccF-NH_2_ showed a significant reduction in activity (IC_50_ = 4.8 nM) compared to both isolated GccF and GccF-NH_2_, suggesting that incorporation of d-His negatively influences bioactivity. However, we herein show that the previously reported reduction in bioactivity of the [^27^d-His]-GccF-NH_2_ was in fact a consequence of the *C*-terminal amidation.

The antibacterial activity of the two glyco-mutants, **10** and **11** revealed an intriguing trend. The activity of glycocin F analogue **10**, which contains two *O*-linked GlcNAc moieties, exhibited decreased activity (approximately 10-fold, IC_50_ = 12.1 ± 0.20 nM) compared to the native glycocin F (IC_50_ = 1.13 ± 0.20 nM). Notably, the activity of analogue **11**, which contains two *S*-linked GlcNAc moieties, was increased by approximately 2-fold (IC_50_ = 0.60 ± 0.10 nM) relative to the native glycopeptide. Thus, in this series, the bacteriostatic activity is enhanced by replacing the *O*-glycoside with an *S*-glycoside, with the maximum activity obtained by glycopeptide **11** bearing *S*-linked GlcNAc at both residues 18 and 43.

This striking result is predicted to be due to the enhanced resistance of the stable *S*-glycosidic linkages to hydrolytic cleavage compared to *O*-glycosidic linkages, when exposed to the cell envelope and/or secreted glycosidases of target bacteria. Indeed, the Pratt group has recently demonstrated the resistance of *S*-linked GlcNAc to cleavage by human *O*-GlcNAcase (OGA).^14^ By avoiding enzymatic cleavage, the *S*-linked GlcNAc moieties could remain available for binding at the GccF target receptor, leading to enhanced potency and prolonged bacteriostasis.

## Conclusions

The first total synthesis of native glycocin F **1**, containing both *S*- and *O*-linked β-GlcNAc moieties, was accomplished using a three-fragment NCL strategy followed by oxidative folding. Structural identity to the naturally isolated glycocin F was confirmed by analytical RP-HPLC and CD spectroscopy. To avoid troublesome racemisation at ^27^His and to increase the overall efficiency of glycocin F synthesis, an alternative synthetic strategy was successfully developed using a two-fragment NCL strategy. This strategy was then employed for the synthesis of two ‘glyco-mutant’ analogues of glycocin F (**10** and **11**), each bearing a single modification at one of the residues bearing a sugar moiety. Strikingly, the bioactivity increased significantly when the sequence incorporated two *S*-GlcNAc moieties at positions 18 and 43 (in peptide **11**).

The results reported herein highlight the potential of glycocin F analogue **11** and related *S*-linked glycoconjugates as leads for the development of new anti-bacterial agents. Our preparation of glycocin F and analogues aims to provide fundamental insights into a novel antimicrobial mechanism of action, knowledge that is vital for the successful conversion of natural products into the ‘smart antibiotics’ needed for future therapies and industrial applications.

## Conflicts of interest

There are no conflicts to declare.

## Supplementary Material

Supplementary information
