# Supplementary material for: Total chemical synthesis of glycocin F and analogues: S-glycosylation confers improved antimicrobial activity
Source: Chem Sci. 2018 Jan 12;9(6):1686–91. doi: 10.1039/c7sc04383j (PMC5890784; doi:10.1039/c7sc04383j)
Supplement: Supplementary file 1 [file SC-009-C7SC04383J-s001.pdf]

Electronic Supplementary Material (ESI) for Chemical Science.
This journal is © The Royal Society of Chemistry 2018

Total chemical synthesis of glycocin F and analogues: *S*-glycosylation confers improved antimicrobial activity

Zaid Amso,[a] Sean W. Bisset,[bc] Sung-Hyun Yang,[a] Paul W. R. Harris,[acd] Tom H. Wright,[a] Caudio. D. Navo,[e] Mark L. Patchett,[b] Gillian E. Norris,[bc] and Margaret A. Brimble[*acd]

[a.] *School of Chemical Sciences, The University of Auckland, 23 Symonds St, Auckland 1142, New Zealand. E-mail: m.brimble@auckland.ac.nz; Fax: +64 9 3737422; Tel: +64 9 3737599*

[b.] *Institute of Fundamental Sciences, Massey University, Colombo Rd, Palmerston North 4442, New Zealand.*

[c.] *Maurice Wilkins Centre for Molecular Biodiscovery, The University of Auckland, Private Bag 92019, Auckland 1142, New Zealand.*

[d.] *School of Biological Sciences, The University of Auckland, 3 Symonds St, Auckland 1142, New Zealand*

[e.] *Dept. Química, Universidad de La Rioja, Centro de Investigación en Síntesis Química, E-26006 Logroño, Spain.*

Supporting information

Table of Contents

1. General information 3

1.1 Materials 3

1.2 TLC, HPLC, MS and NMR 4

2. Synthesis of glycoside building blocks **S6** and **S11** 5

2.1 Synthesis of chloro-GlcNAc(OAc)$_3$ **S2** 6

2.2 Synthesis of Fmoc-Ser-O^{t}Bu **S4** 6

2.3 Synthesis of glycoside **S6** 7

2.4 Synthesis of bromide **S9** 8

2.5 Synthesis of thio-GlcNAc(OAc)$_3$ **S8** 9

2.6 Synthesis of glycoside **S11** 9

3. ^{1}H and ^{13}C NMR Spectra 11

4. Peptide synthesis 16

4.1 Synthesis of GccF (**1b**) and *epi*-GccF (**1a**) using three fragments pathway 16

4.1.1 Synthesis of peptide **6** 16

4.1.2 Synthesis of peptide **3** 18

4.1.3 Synthesis of peptide **2** 23

4.1.4 Synthesis of polypeptide **5a** and **5b** 24

4.1.5 Synthesis of polypeptide **7a** and **7b** 26

4.1.6 Synthesis of polypeptide **8a** and **8b** 29

4.1.7 Synthesis of *epi*-(*D*-^{27}His)-GccF **1a** and native GccF **1b** 32

4.2 Optimised synthesis of fragment H_2N-^{12}Gly-^{27}His-COOH, by Fmoc-SPPS 36

4.3 Synthesis of 'Glyco-mutant' of GccF **10** and **11** 37

4.3.1 Direct synthesis of peptides **14** and **15** 37

4.3.2 Synthesis of polypeptides **S21** and **S22** 39

4.3.3 Synthesis of polypeptides **S23** and **S24** 42

4.3.4 Synthesis of 'Glyco-mutant' **10** and **11** 44

5. Circular Dichroism (CD) spectroscopy 47

6. Antibacterial Assay 48

7. References 49

1. General information

1.1 Materials

All reagents were acquired as reagent grade and used without further purification. Solvents for RP-HPLC were purchased as RP-HPLC grade and used without further purification. Unless mentioned, all organic chemistry experiments were executed under an oxygen-free atmosphere of nitrogen using standard techniques.

6-Chloro-1-hydroxybenzotriazole (6-Cl-HOBt), Fmoc-Gly-Thr($\psi^{Me,Me}$pro)-OH, Fmoc-Tyr(*t*Bu)-Ser($\psi^{Me,Me}$pro)-OH, and Fmoc-Ser(*t*Bu)-Ser($\psi^{Me,Me}$pro)-OH were purchased from Aapptec (Louisville, Kentucky). 2-(7-Aza-1*H*-benzotriazole-1-yl)-1,1,3,3-tetramethyluronium hexafluorophosphate (HATU), *O*-(6-chlorobenzotriazol-1-yl)-*N*,*N*,*N′*,*N″*-tetramethyluronium hexafluorophosphate (HCTU), 1-hydroxy-7-azabenzotriazole (HOAt), 2-chlorotrityl chloride resin, and *S*-trityl-3-mercaptopropionic acid were purchased from GL Biochem (Shanghai, China). *N,N*-dimethylformamide (DMF) (AR grade), and acetonitrile (CH_3CN) [high-performance liquid chromatography (HPLC) grade] were purchased from Scharlau (Barcelona, Spain). *N,N′*-diisopropylethylamine (*i*Pr_2EtN), triisopropylsilane (*i*Pr_3SiH), 2,2′-(ethylenedioxy)-diethanethiol (DODT), *N,N′*-diisopropylcarbodiimide (DIC), *N*,*N*-dimethylaminopyridine (DMAP), 2,2,2-trifluoroethanol (TFE), 1,2-ethanedithiol (EDT), formic acid, 2,2,6,6-tetramethylpyridine (TMP), 4-mercaptophenylacetic acid (MPAA), tris(2-carboxyethyl)hydrochloride (TCEP.HCl), guanidine hydrochloride (Gn.HCl), 4-(2-hydroxyethyl)-1-piperazineethanesulfonic acid (HEPES), tetrabutylammonium iodide (TBAI), methoxyamine.HCl, *p*-cresol, dimethyl sulfide (Me_2S), (ethylenedinitrilo)tetraacetic acid (EDTA), benzyl mercaptan, hydrazine hydrate, 2-mercaptoethanol, L-cysteine, L-cystine, and Fmoc-Ser-OH were purchased from Sigma–Aldrich (Sydney, Australia). Aminomethyl polystyrene resin (AM-PS) was purchased from Rapp Polymere (Tuebingen, Germany). Dichloromethane (CH_2Cl_2) was purchased from ECP Limited (Auckland, New Zealand). Boc-Lys(2-Cl-Z)-PAM (Z = benzyloxycarbonyl; PAM = phenylacetamidomethyl) was purchased from PolyPeptide Laboratories Group (Strasbourg, France). Diethyl ether (Et_2O) was purchased from Avantor Performance Materials. Trifluoroacetic acid (TFA) was purchased from Halocarbon (River Edge, USA). 4-(4-Hydroxymethyl-3-methoxyphenoxy)-butyric acid (HMPB linker) was purchased from NovaBioChem (Läufelfingen, Switzerland). Anhydrous hydrogen fluoride (HF) was purchased from Matheson Trigas (Basking Ridge, New Jersey).

The following Fmoc-amino acids were purchased from GL Biochem: Fmoc-Gly-OH, Fmoc-Ala-OH, Fmoc-Met-OH, Fmoc-Phe-OH, Fmoc-Ile-OH, and Fmoc protected amino acids with the following side-chain protection: Fmoc-Tyr(*t*Bu)-OH (*t*Bu = *tert*-butyl), Fmoc-Asp(*t*Bu)-OH,

Fmoc-Ser(*t*Bu)-OH, Fmoc-Thr(*t*Bu)-OH, Fmoc-Cys(Trt)-OH (Trt = triphenylmethyl), Fmoc-His(Trt)-OH, Fmoc-Lys(Boc)-OH.

The following Boc-amino acids were purchased from PolyPeptide laboratories: Boc-Pro-OH, Boc-Ala-OH, Boc-Leu-OH.H_2O, Boc-Met(O)-OH, Boc-Thz-OH (Thz = thiazolidine), and Boc protected amino acids with the following side-chain protection: Boc-Lys(2-CL-Z)-OH, Boc-Tyr(2-Br-Z)-OH, Boc-Trp(CHO)-OH (CHO = formyl), Boc-Cys(4-MeBn)-OH (Bn = benzyl), Boc-Thr(Bn)-OH.

1.2 TLC, HPLC, MS and NMR

Thin-layer chromatography (TLC) was performed with Merck silica gel plates using UV light as the visualising agent and/or developed using an ethanolic solution of vanillin. Analytical RP-HPLC spectra were performed on a Dionex (California, USA) Ultimate 3000 System equipped with a two-channel UV detector using the analytical column XTerra® MS C_{18}, 4.6 mm x 150 mm, 5 µm or Grace Vydac 219TP Diphenyl 4.6 x 250 mm, 5 µm and a linear gradient of either 5% to 75%B over 35 min or 5% to 65% over 30 min (*ca.* 2%B per minute) at a flow rate of 1 mL/min. The solvent system used was A (0.1% TFA in H_2O) and B (0.1% TFA in CH_3CN). The ratio of products was determined by integration of spectra recorded at 210 nm or 214 nm.

Peptides were purified using a Waters (Massachusetts, USA) S600E system using a semi-preparative column (Waters XTerra® C_{18}, 300 mm x 19 mm, 10 µm) at a flow rate of 10 mL/min and eluted using a one-step slow gradient protocol with detection at 210 nm.[1, 2] Fractions were collected, analysed by analytical RP-HPLC or ESI-MS, pooled and lyophilised.

Peptide masses were confirmed by a Bruker micrOTOF-Q II mass spectrometer (Bremen, Germany) or a Hewlett Packard (HP) 1100 series mass spectrometer (California, USA) using direct flow injection at 0.3 mL/min into an ESI source in the positive mode.

NMR experiments were performed at room temperature in $CDCl_3$ and $CDCl_3/CD_3OD$ (*v/v*; 9:1) on a Bruker (Billerica, Massachusetts, U.S) BRX400 spectrometer operating at 400 MHz for ^1H nuclei and 100 MHz for ^{13}C nuclei. ^1H-NMR spectra were recorded with 32768 data points and with spectral width of 15.9793 ppm. ^{13}C-NMR spectra were recorded with 65539 data points and with spectral width of 238.8941 ppm. Spectra were analysed with Topspin (Bruker, Germany) software. Chemical shifts were reported in parts per million (ppm) on the δ scale downfield from tetramethylsilane as a reference. J Coupling constants were reported in Hertz (Hz). Multiplicities were reported as s = singlet, d = doublet, dd = doublet of doublets, ddd = doublet of doublets of doublets, t = triplet, and m = multiplet.

2 Synthesis of glycoside building blocks S6 and S11

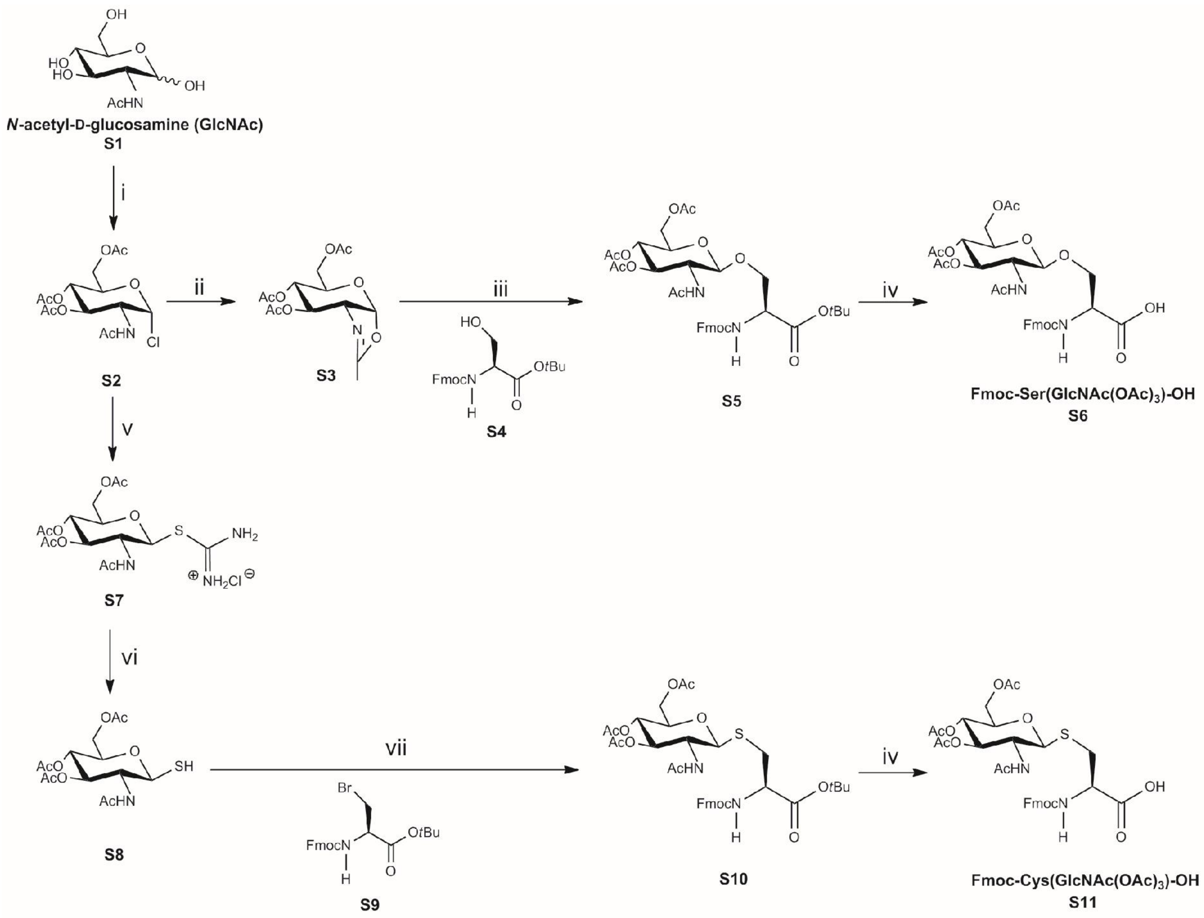

Scheme 1. Synthesis of Fmoc-*L*-Ser(β-GlcNAc(OAc)$_3$)-OH **S6** and Fmoc-*L*-Cys(β-GlcNAc(OAc)$_3$)-OH **S11**. *Reagents and conditions*: (i) acetyl chloride, r.t, 48 h, 70%; (ii) TMSOTf, 1,2-dichloroethane, reflux, 3 h, 68%; (iii) Anh. $CuCl_2$, 1,2-dichloroethane, reflux, 24 h, 44%; (iv) TFA:H_2O (9:1, *v*/*v*), r.t, 1 h, quant. for **S6** and quant. for **S11**; (v) thiourea, acetone, reflux, 2 h; (vi) $Na_2S_2O_5$, CH_2Cl_2, H_2O, reflux, 3 h, 86% over 3 steps from **S1**; (vii) tetrabutylammonium sulfate, 5% aq. $NaHCO_3$:EtOAc (1:1, *v*/*v*), 50 ^oC, 3 h, 95%.

2.1 Synthesis of 2-acetamido-2-deoxy-3,4,6-tri-*O*-acetyl-1-chloro-α-*D*-glucopyranose (S2)

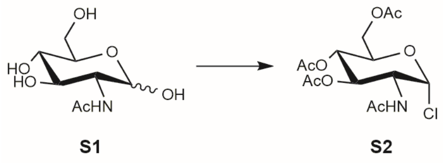

N-acetyl-D-glucosamine (GlcNAc) **S1** (5.0 g, 22.6 mmol) was dissolved in acetyl chloride (30 mL), the solution was sealed tight and stirred at room temperature for 2 days.[3] Upon completion of the reaction, as judged by TLC, the solution was diluted with CH_2Cl_2 (80 mL) and transferred slowly into ice water (300 mL). The organic layer was separated and the aqueous phase was extracted with CH_2Cl_2 (3 × 50 mL). The organic layers were pooled and their pH was adjusted to 7.0 by the addition of saturated aq. $NaHCO_3$. The combined organic extracts were then dried over sodium sulfate, filtered and concentrated *in vacuo*. The crude product was purified by flash column chromatography on silica gel (EtOAc/petroleum ether, 1:1) to afford compound **S2** (5.8 g, 70%) as a light brown solid. ^{1}H NMR and ^{13}C NMR data were in agreement with that reported in the literature.[4, 5]

^{1}H NMR (400 MHz, $CDCl_3$): δ 6.19 (d, *J* 3.7 Hz, 1H, H-1), 5.82 (d, *J* 8.7 Hz, 1H, H-2), 5.35-5.30 (m, 1H, H-3), 5.24-5.19 (m, 1H, H-4), 4.57-4.51 (m, 1H, H-5), 4.31-4.25 (m, 2H, H-5, H-6_a), 4.16-4.11 (m, 1H, H-6_b), 2.06 (s, 6H, COCH_3), 2.06 (s, 3H, COCH_3), 1.99 (s, 3H, COCH_3); **^{13}C NMR (100 MHz, $CDCl_3$):** δ 171.6, 170.7, 170.3, 169.3, 93.8, 71.1, 70.3, 67.2, 61.3, 53.6, 23.2, 20.8, 20.7.

2.2 *tert*-Butyl *N*-(9-fluorenylmethoxycarbonyl)-*L*-serine (S4)

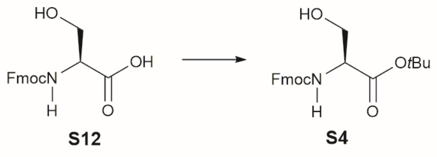

A mixture of DCC (3.78 g, 18.3 mmol) and a catalytic amount of freshly prepared Cu(I)Cl were added to freshly distilled (from calcium hydride) *tert*-butanol (1.76 mL, 18.3 mmol), and the mixture was stirred at room temperature for 5 days.[6] The reaction was then diluted with CH_2Cl_2 (10 mL) followed by a dropwise addition of a solution of Fmoc-*L*-Ser-OH **S12** (1.50 g, 4.58 mmol) in THF/CH_2Cl_2 (*v/v*; 1:9; 10 mL). Upon completion of the reaction as judged by TLC (usually takes 3 h), the reaction was diluted with CH_2Cl_2 (20 mL), then filtered, washed with saturated aq. NH_4Cl (3 × 20 mL), dried over sodium sulfate, and concentrated *in vacuo*. The crude product was purified by flash column chromatography on silica gel (EtOAc/petroleum ether, 7:3) to afford alcohol **S4** (1.62 g, 94%) as a white solid. The ^{1}H NMR and ^{13}C NMR data were in agreement with that reported in the literature.[7]

^{1}H NMR (400 MHz, CDCl$_3$): δ 7.75 (d, *J* 7.6 Hz, 2H, Fmoc-Ar), 7.55 (d, *J* 7.4 Hz, 2H, Fmoc-Ar), 7.39 (t, 7.4 Hz, 2H, Fmoc-Ar), 7.31 (m, 2H, Fmoc-Ar), 4.41 (d, *J* 6.9 Hz, 2H, C*H$_2$* Fmoc), 4.32 (s, 1H, C*H* Fmoc), 4.21 (t, *J* 6.92 Hz, 1H, C*H$_2$* Ser), 3.92 (s, 1H, C*H*α Ser), 1.48 (s, 9H, COOC(C*H$_3$*)$_3$); **^{13}C NMR (100 MHz, CDCl$_3$):** δ 169.7, 156.5, 144.0, 143.9, 141.5, 141.5, 127.9, 127.3, 125.3, 120.2, 83.1, 67.4, 63.9, 56.8, 47.4, 28.2.

2.3 *N*-(9-fluorenylmethoxycarbonyl)-(2-acetamido-2-deoxy-3,4,6-tri-*O*-acetyl-β-*D*-gluco pyranosyl)-*L*-serine (S6)

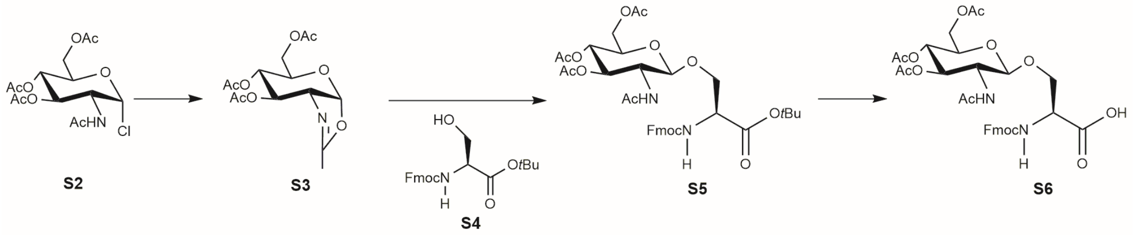

α-Cl-GlcNAc(OAc)$_3$ **S2** (3.0 g, 7.71 mmol) was dissolved in anhydrous 1,2-dichloroethane (25 mL). Freshly distilled TMSOTf[8] (2.8 mL, 15.4 mmol) was then added and the mixture was heated under reflux. Upon completion of the reaction as judged by TLC (usually 3 h), triethylamine was added to quench the residual TMSOTf and the solvent was removed *in vacuo*. The crude product was purified by flash column chromatography on silica gel (CH$_2$Cl$_2$/MeOH, 99:1) to afford oxazoline **S3** (1.70 g, 68%) as a brown oil.

Oxazoline **S3** (0.44 g, 1.33 mmol) was dissolved in 1,2-dichloroethane (15 mL) to which anhydrous CuCl$_2$[9] (0.18 g, 1.33 mmol) and Fmoc-Ser-O*t*Bu **S4** (1.02 g, 2 mmol) was added. The reaction mixture was then heated under reflux until completion of the reaction, as judged by TLC (usually 24 h). The reaction mixture was then cooled to room temperature, and the solvent was removed *in vacuo.* The crude product was then diluted with EtOAc, and the organic layer was washed with 2 M HCl followed by saturated aq. NaHCO$_3$ (50 mL) and brine. Following the washing steps, the organic layer was dried over sodium sulfate, filtered and concentrated *in vacuo* to yield the *tert*-butyl-protected glycoside **S5** as an off-white solid (416 mg, 44%).

The *tert*-butyl ester **S5** (0.416 g, 0.58 mmol) was then treated with a mixture of trifluoroacetic acid and water (*v*/*v*; 9:1, 20 mL) at room temperature. Upon completion of the reaction, as judged by TLC (usually 1 h), the reaction mixture was concentrated *in vacuo* followed by re-dissolving in toluene and evaporation which was repeated twice to remove traces of trifluoroacetic acid. The crude product was purified by flash column chromatography on silica gel (CH$_2$Cl$_2$/MeOH/AcOH, 94:5:1) to afford compound **S6** (380 mg, quant.) as a white solid. The ^{1}H NMR and ^{13}C NMR data were in agreement with that reported in the literature.[10]

HRESI-MS (EI): *m/z* $[M+Na]^+$ calculated for $C_{32}H_{36}N_2NaO_{13}$: 679.2217, found: 679.2110; **^{1}H NMR (400 MHz, $CDCl_3/CD_3OD$ (*v/v*; 9:1)):** δ 7.77 (d, *J* 7.5 Hz, 2H, Fmoc-Ar), 7.63 (d, *J* 7.1 Hz, 2H, Fmoc-Ar), 7.39 (t, *J* 7.5 Hz, 2H, Fmoc-Ar), 7.32 (m, 2H, Fmoc-Ar), 5.18 (t, *J* 9.7 Hz, 1H, H-3), 5.01 (t, *J* 9.4 Hz, 1H, H-4), 4.56 (d, *J* 8.3 Hz, 1H, H-1), 4.51-4.37 (m, 3H, CH_{2a} Fmoc, C*H*α Ser, CH_{2b} Fmoc), 4.26-4.10 (m, 4H, H-6_a, C*H* Fmoc, CH_{2a} Ser, H-6_b), 3.88-3.84 (m, 2H, CH_{2b} Ser, H-2), 3.67 (ddd, *J* 9.9, 4.5, 2.4 Hz, 1H, H-5), 2.07 (s, 3H, COCH_3), 2.03 (s, 3H, COCH_3), 2.02 (s, 3H, COCH_3), 1.84 (s, 3H, COCH_3); **^{13}C NMR (100 MHz, $CDCl_3/CD_3OD$ (*v/v*; 9:1)):** δ 171.5, 170.7, 170.5, 169.2, 155.9, 143.3, 143.2, 140.8, 127.3, 126.7, 124.5, 119.5, 100.5, 71.9, 71.2, 68.8, 68.2, 66.3, 61.6, 53.9, 53.4, 46.7, 22.0, 20.1, 20.0.

Acquisition Parameter

Source Type	ESI	Ion Polarity	Positive	Set Nebulizer	0.4 Bar
Focus	Not active	Set Capillary	4500 V	Set Dry Heater	180 °C
Scan Begin	50 m/z	Set End Plate Offset	-500 V	Set Dry Gas	4.0 l/min
Scan End	1000 m/z	Set Collision Cell RF	150.0 Vpp	Set Divert Valve	Waste

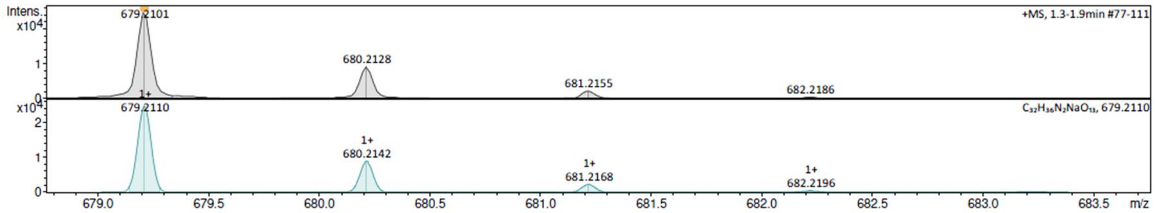

Meas. m/z	#	Ion Formula	m/z	err [ppm]	mSigma	# Sigma	Score	rdb	e⁻ Conf	N-Rule
679.2101	1	C32H33N5O12	679.2120	2.9	2.0	1	81.63	19.0	odd	ok
	2	C32H152N9O	679.2114	2.0	13.4	2	100.00	-38.5	even	ok
	1	C32H33N5O12	679.2120	2.9	2.0	1	81.63	19.0	odd	ok
	2	C32H152N9O	679.2114	2.0	13.4	2	100.00	-38.5	even	ok
	1	C32H36N2NaO13	679.2110	-1.3	6.9	1	79.87	15.5	even	ok
	2	C32H155N6NaO2	679.2104	0.5	10.6	2	100.00	-42.0	odd	ok
	3	C32H26N13NaO4	679.2123	-3.3	12.2	3	29.41	26.0	odd	ok

2.4 *tert*-Butyl *N*-(9-fluorenylmethoxycarbonyl)-β-bromo-*L*-alanine (S9)

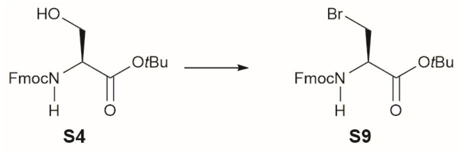

Fmoc-Ser-O*t*Bu **S4** (1.65 g, 4.30 mmol) was dissolved in CH_2Cl_2 (90 mL) and cooled to 0 °C. To this solution, carbon tetrabromide (2.85 g, 8.61 mmol) and triphenylphosphine (2.26 g, 8.61 mmol) was then added. The reaction mixture was allowed to warm to room temperature, then stirred for 3 h and dried over sodium sulfate. The reaction mixture was then filtered and concentrated *in vacuo* to afford compound **S9** (1.5 g, 78%) as a light brown solid which was used in the next step without further purification

2.5 Synthesis of 2-acetamido-2-deoxy-3,4,6-tri-*O*-acetyl-1-thio-β-*D*-glucopyranose (S8)

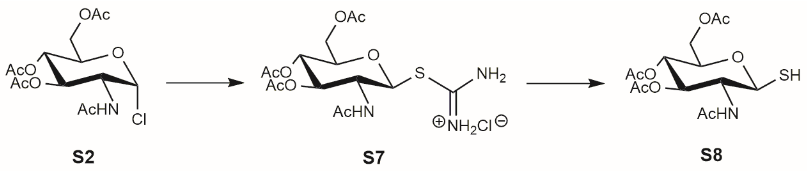

α-Cl-GlcNAc(OAc)$_3$ **S2** (4.0 g, 11.3 mmol) was dissolved in acetone (60 mL). To this solution, thiourea (1.5 g, 20.1 mmol) was added and the mixture heated under reflux for 2 h, after which a white precipitate was observed.[3] The reaction was cooled to room temperature and the precipitate was filtered. The filtrate was repeatedly heated under reflux and filtered until the solid ceased to precipitate. The generated white solid **S7** was used in the next step without further purification.

Thiourea **S7** (4.5 g, 10.2 mmol) was dissolved in CH_2Cl_2/H_2O (*v/v*; 3:2, 50 mL). To this mixture, sodium metabisulfite (2.5 g, 12.7 mmol) was added and the reaction was heated under reflux for 3 h then cooled to room temperature.[3] The organic phase was separated and the aqueous phase was extracted with CH_2Cl_2 (3 × 50 mL). The combined organic extracts were washed with brine (50 mL), water (50 mL) and dried over sodium sulphate, filtered and concentrated *in vacuo*. The crude product **S8** (3.2 g, 86% over three steps from *N*-acetyl-D-glucosamine **S1** was recovered as a white solid. ^{1}H NMR and ^{13}C NMR data were in agreement with that reported in the literature.[11]

^{1}H NMR (400 MHz, CDCl$_3$): δ 5.56 (d, *J* 9.6 Hz, 1H, H-1), 5.15-5.05 (m, 2H, H-3, H4), 4.57 (dd, *J* 10.0, 9.5 Hz, 1H, H-2), 4.24 (dd, *J* 12.4, 4.8 Hz, 1H, H-6$_a$), 4.14 (m, 1H, H-6$_b$), 3.70-3.66 (m, 1H, H-5), 2.10 (s, 3H, COC*H$_3$*), 2.04 (s, 3H, COC*H$_3$*), 2.03 (s, 3H, COC*H*$_3$), 1.98 (s, 3H, COC*H*$_3$); **^{13}C NMR (100 MHz, CDCl$_3$):** δ 171.2, 170.7, 170.4, 169.2, 80.3, 76.3, 73.5, 68.1, 62.1, 56.8, 23.3, 20.7, 20.6, 20.5.

2.6 *N*-(9-Fluorenylmethoxycarbonyl)-(2-acetamido-2-deoxy-3,4,6-tri-*O*-acetyl-β-*D*-gluco pyranosyl)-*L*-cysteine (S11)

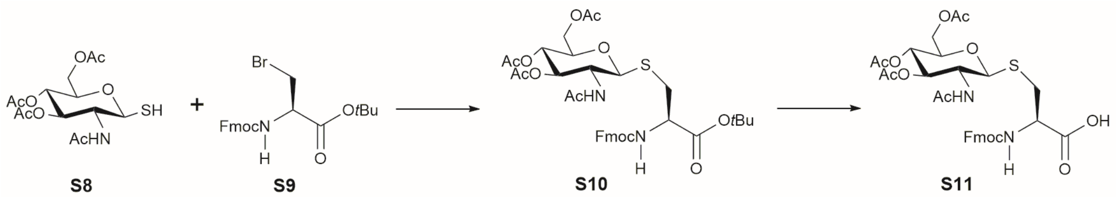

β-GlcNAc(OAc)$_3$-thiol **S8** (1.60 g, 4.41 mmol) and bromide **S9** (1.31 g, 2.94 mmol) in EtOAc (56 mL) were first added to a 5% (*v/v*) solution of $NaHCO_3$ in water (*w/v*; 56 mL). Tetrabutylammonium hydrogensulfate (4.0 g, 11.7 mmol) was then added and the mixture was stirred vigorously at 50 °C.

Upon completion of the reaction, as judged by TLC (usually 3 h), the organic phase was separated and the aqueous layer was extracted with EtOAc (3 × 50 mL). The combined organic layers were then washed with saturated aq. $NaHCO_3$ (50 mL), dried over sodium sulfate, filtered and concentrated *in vacuo* to yield the *tert*-butyl-protected glycoside **S10** as an off-white solid (2.03 g, 95%).

The protected thioglycoside **S10** (0.56 g, 0.77 mmol) was then treated with a mixture of TFA/H_2O (*v*/*v*; 9:1, 20 mL) at room temperature. Upon completion of the reaction, as judged by TLC (usually 1 h), the reaction mixture was concentrated *in vacuo* followed by re-dissolving in toluene and evaporation which was repeated twice to remove traces of trifluoroacetic acid. The crude product was purified by flash column chromatography on silica gel (CH_2Cl_2/MeOH, 95:5) to afford compound **S11** (0.51 g, 100%) as an off-white solid. The ^{1}H NMR and ^{13}C NMR data were in agreement with that reported in the literature.[12]

HRESI-MS (EI): *m/z* $[M+Na]^+$ calculated for $C_{32}H_{36}N_2NaO_{1s}S$: 695.1989, found: 695.1881; **^{1}H NMR (400 MHz, $CDCl_3/CD_3OD$ (*v/v*; 9:1)):** δ 7.77 (d, *J* 7.5 Hz, 2H, Fmoc-Ar), 7.66-7.60 (m, 2H, Fmoc- Ar), 7.41 (t, *J* 7.5 Hz, 2H, Fmoc-Ar), 7.35-7.31 (m, 2H, Fmoc-Ar), 5.14-5.03 (m, 2H, H-3, H-4), 4.68 (d, *J* 10.4 Hz, 1H, H-1), 4.56-4.49 (m, 1H, C*H*α Cys), 4.46-4.35 (m, 2H, C*H₂* Fmoc), 4.26-4.22 (t, *J* 6.9 Hz, 1H, C*H* Fmoc), 4.19-4.15 (m, 2H, H-6_a, H-6_b), 4.09-4.04 (t, *J* 9.9 Hz, 1H, H-2), 3.68-3.66 (m, 1H, H-5), 3.35-3.30 (m, 1H, CH_{2a} Cys), 2.96-2.90 (m, 1H, CH_{2b} Cys), 2.06 (s, 3H, $COCH_3$), 2.03 (s, 3H, $COCH_3$), 2.02 (s, 3H, $COCH_3$), 1.86 (s, 3H, $COCH_3$); **^{13}C NMR (100 MHz, $CDCl_3/CD_3OD$ (*v/v*; 9:1)):** δ 171.2, 171.0, 170.9, 169.4, 156.4, 143.6, 143.5, 141.0, 127.6, 126.9, 124.8, 119.8, 83.4, 75.6, 73.7, 68.4, 66.9, 62.1, 53.3, 52.5, 46.9, 31.9, 22.4, 20.3.

Acquisition Parameter

Source Type	ESI	Ion Polarity	Positive	Set Nebulizer	0.4 Bar
Focus	Not active	Set Capillary	4500 V	Set Dry Heater	180 °C
Scan Begin	50 m/z	Set End Plate Offset	-500 V	Set Dry Gas	4.0 l/min
Scan End	1000 m/z	Set Collision Cell RF	150.0 Vpp	Set Divert Valve	Waste

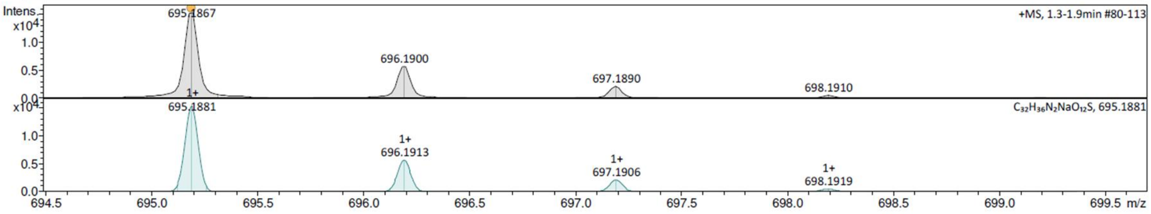

Meas. m/z	#	Ion Formula	m/z	err [ppm]	mSigma	# Sigma	Score	rdb	e⁻ Conf	N-Rule
695.1867	1	C32H33N5O11S	695.1892	3.6	3.7	1	78.64	19.0	odd	ok
	2	C32H152N9S	695.1886	2.8	16.2	2	100.00	-38.5	even	ok
	1	C32H33N5O11S	695.1892	3.6	3.7	1	78.64	19.0	odd	ok
	2	C32H152N9S	695.1886	2.8	16.2	2	100.00	-38.5	even	ok
	1	C32H36N2NaO12S	695.1881	2.1	3.6	1	83.87	15.5	even	ok
	2	C32H155N6NaOS	695.1875	1.2	13.3	2	100.00	-42.0	odd	ok

3. ^{1}H and ^{13}C NMR Spectra

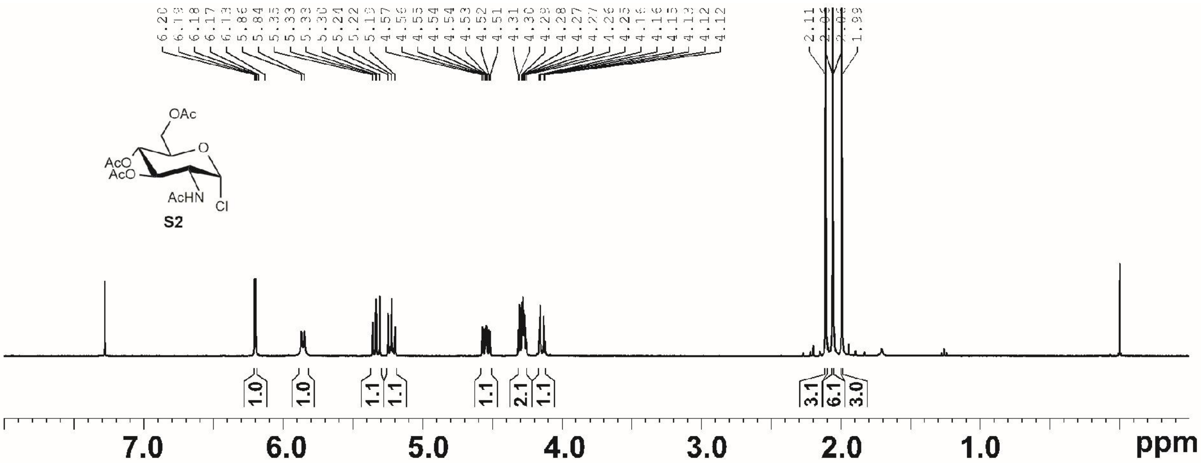

Figure 1. ^{1}H (400 MHz, $CDCl_3$) spectrum of 2-acetamido-2-deoxy-3,4,6-tri-*O*-acetyl-1-chloro-α-*D*-glucopyranose (**S2**)

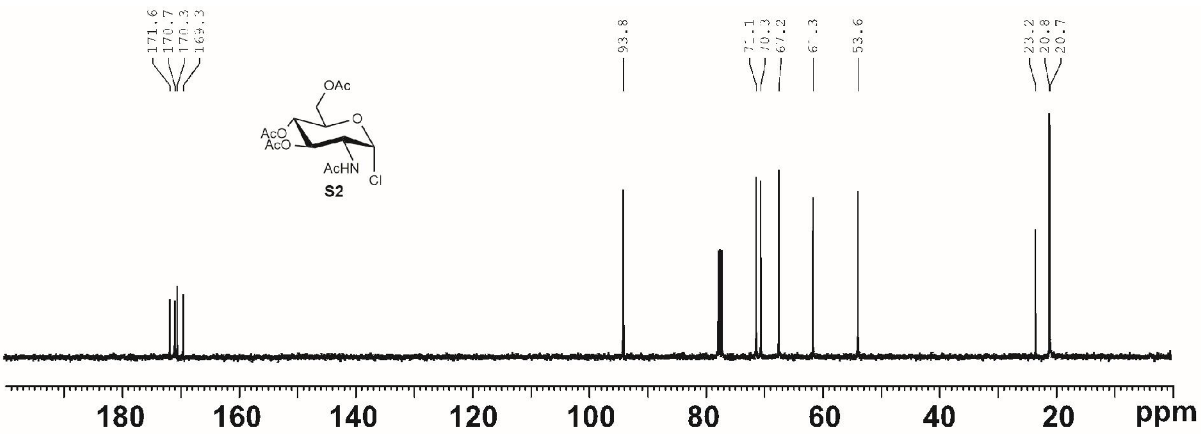

Figure 2. ^{13}C (100 MHz, $CDCl_3$) spectrum of 2-acetamido-2-deoxy-3,4,6-tri-*O*-acetyl-1-chloro-α-*D*-glucopyranose (**S2**)

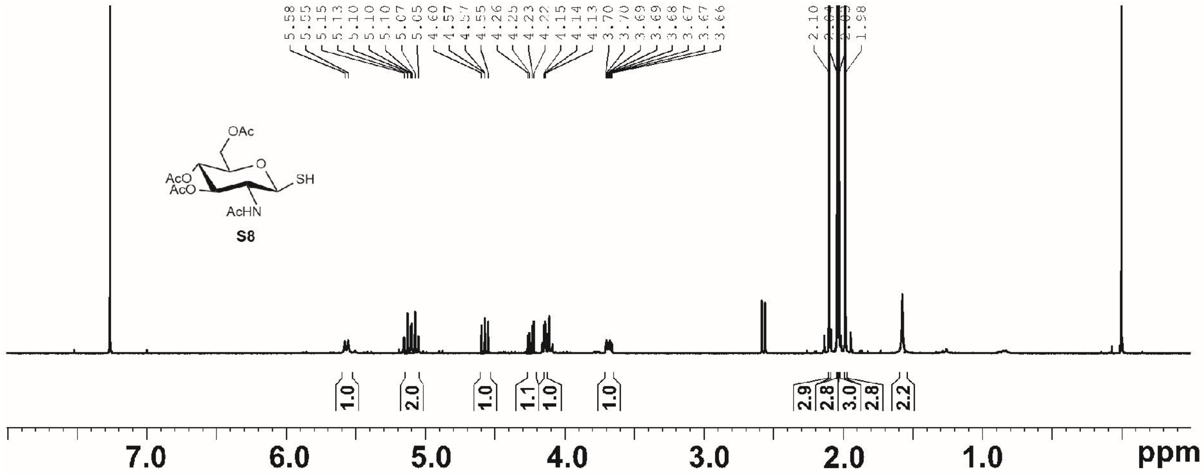

Figure 3. ^{1}H (400 MHz, $CDCl_3$) spectrum of 2-acetamido-2-deoxy-3,4,6-tri-*O*-acetyl-1-thio-β-*D*-glucopyranose (**S8**)

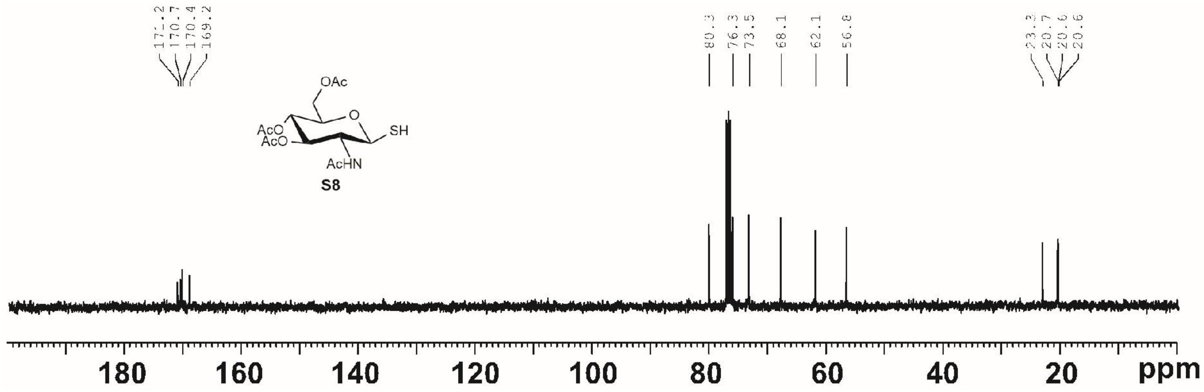

Figure 4. ^{13}C (100 MHz, $CDCl_3$) spectrum of 2-acetamido-2-deoxy-3,4,6-tri-*O*-acetyl-1-thio-β-*D*-glucopyranose (**S8**)

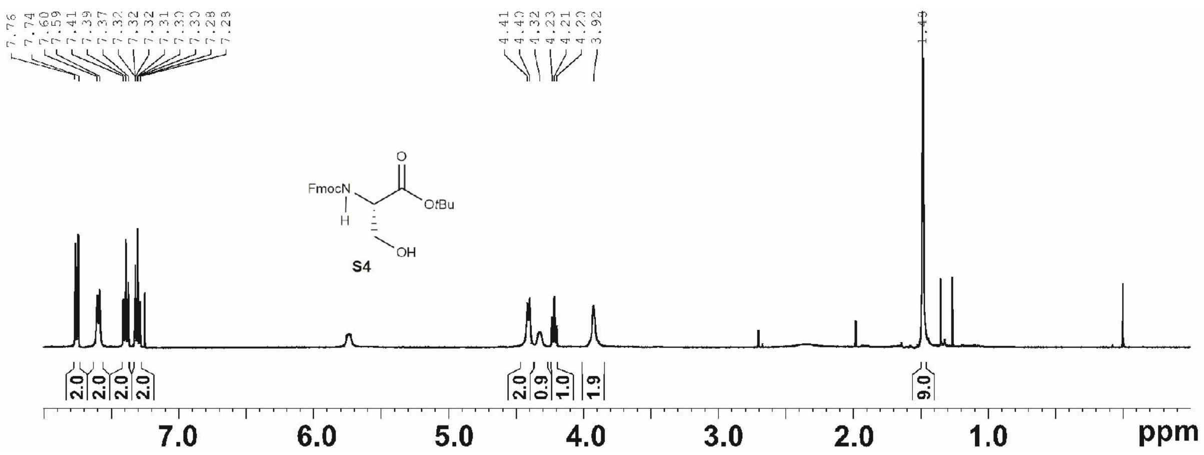

Figure 5. ^{1}H (400 MHz, $CDCl_3$) spectrum of *tert*-butyl *N*-(9-fluorenylmethoxycarbonyl)-*L*-serine (**S4**)

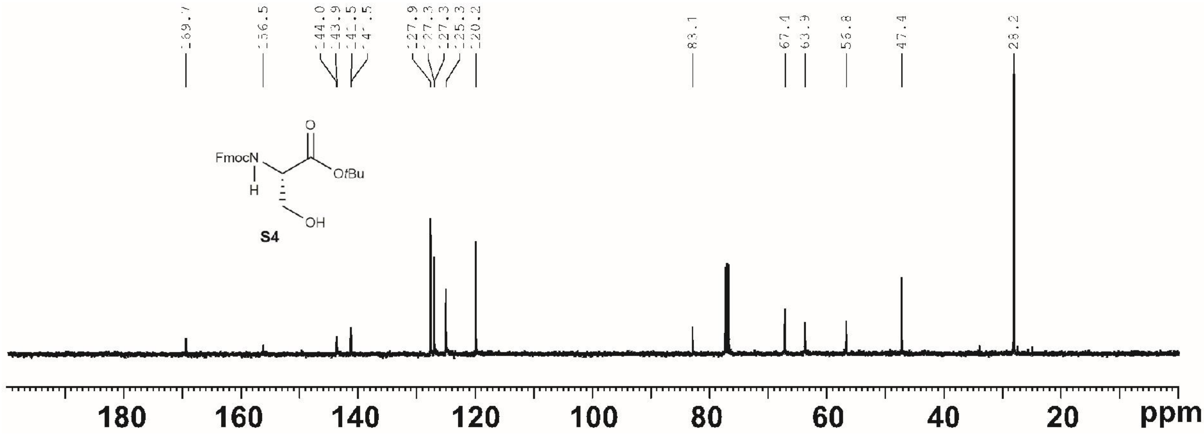

Figure 6. ^{13}C (100 MHz, $CDCl_3$) spectrum of *tert*-butyl *N*-(9-fluorenylmethoxycarbonyl)-*L*-serine (**S4**)

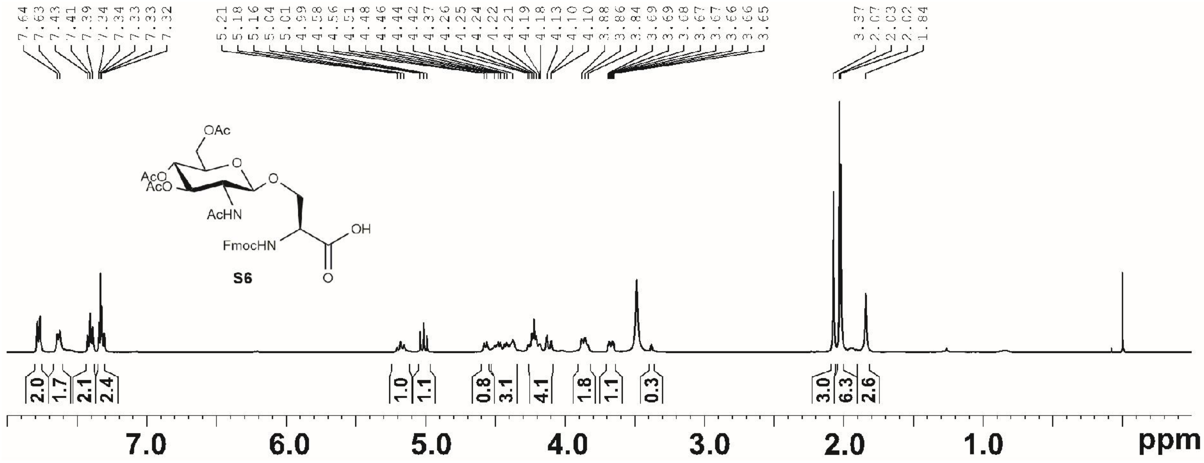

Figure 7. ^{1}H (400 MHz, $CDCl_3/CD_3OD$ (*v/v*; 9:1)) spectrum of *N*-(9-fluorenylmethoxycarbonyl)-(2-acetamido-2-deoxy-3,4,6-tri-*O*-acetyl-β-*D*-glucopyranosyl)-*L*-serine (**S6**)

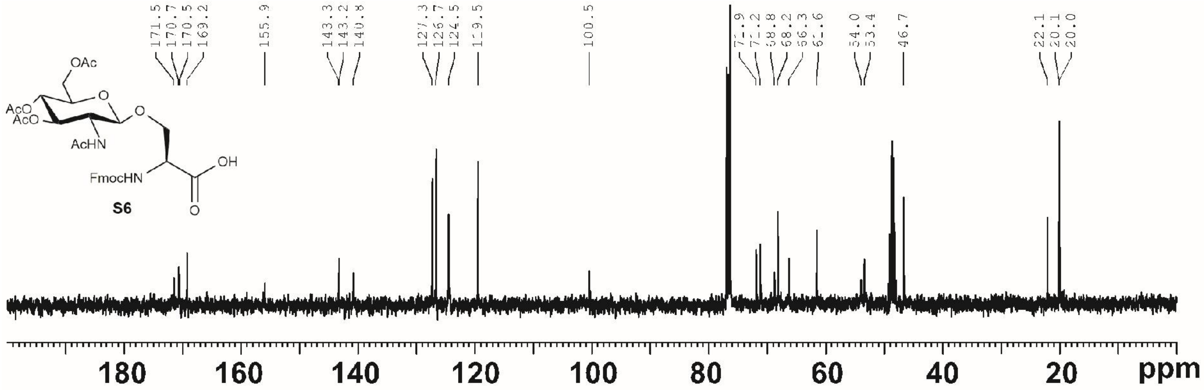

Figure 8. ^{13}C (100 MHz, $CDCl_3/CD_3OD$ (*v/v*; 9:1)) spectrum of *N*-(9-fluorenylmethoxycarbonyl)-(2-acetamido-2-deoxy-3,4,6-tri-*O*-acetyl-β-*D*-glucopyranosyl)-*L*-serine (**S6**)

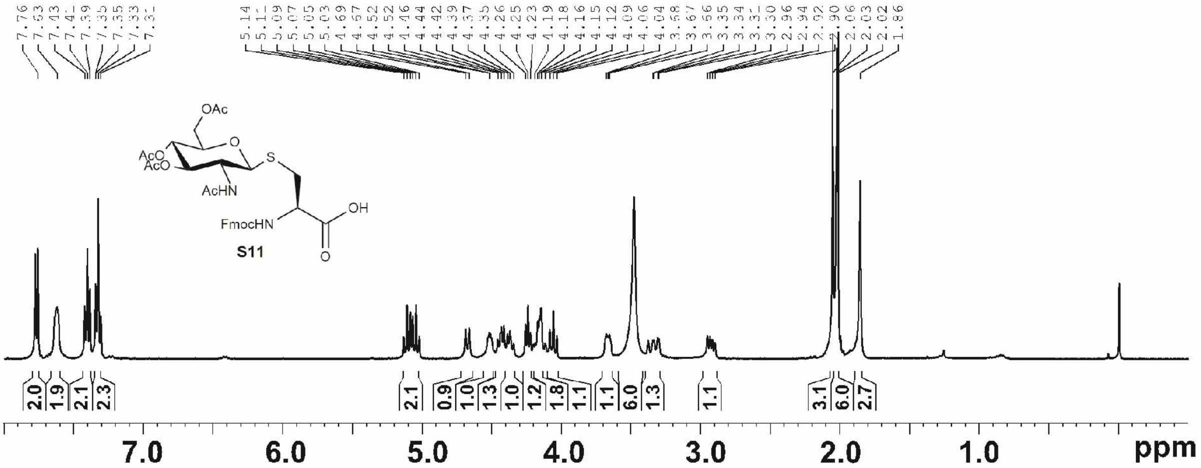

Figure 9. ^{1}H (400 MHz, $CDCl_3/CD_3OD$ (*v/v*; 9:1)) spectrum of *N*-(9-fluorenylmethoxycarbonyl)-(2-acetamido-2-deoxy-3,4,6-tri-*O*-acetyl-β-*D*-glucopyranosyl)-*L*-cysteine (**S11**)

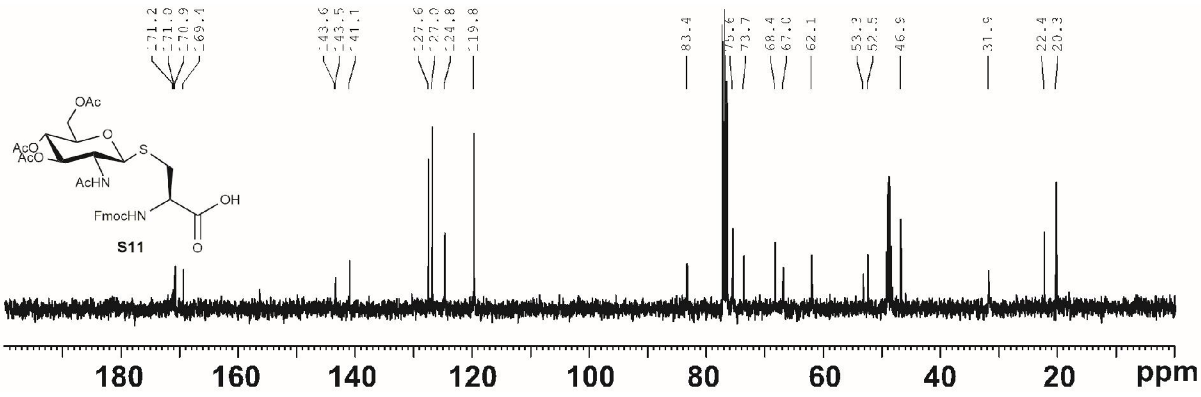

Figure 10. ^{13}C (100 MHz, $CDCl_3/CD_3OD$ (*v/v*; 9:1)) spectrum of *N*-(9-fluorenylmethoxycarbonyl)-(2-acetamido-2-deoxy-3,4,6-tri-*O*-acetyl-β-*D*-glucopyranosyl)-*L*-cysteine (**S11**)

4. Peptide Synthesis

4.1 Synthesis of GccF (1b) and *epi*-GccF (1a), [*D*-[27]His]-GccF, using three fragments pathway

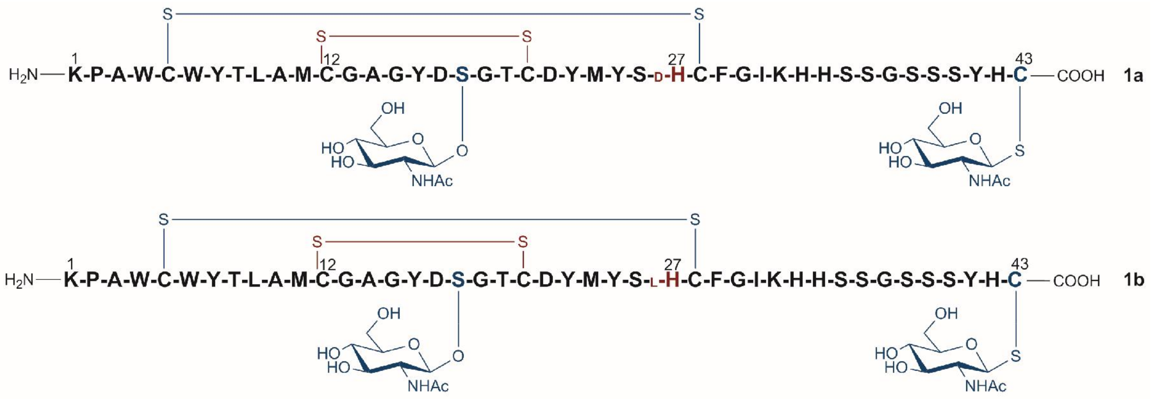

4.1.1 Synthesis of peptide 6

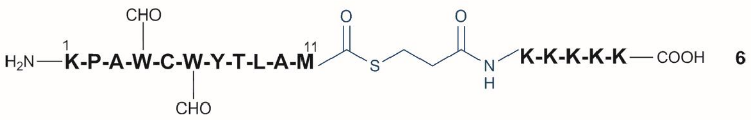

A mixture of Boc-Lys(2-Cl-Z)-PAM linker (166 mg, 0.4 mmol) and DIC (62 µL, 0.4 mmol) in CH_2Cl_2/DMF (*v/v*; 3 mL) was added to pre-swollen (CH_2Cl_2, 3 mL, 20 min) aminomethyl polystyrene resin (220.0 mg, 0.2 mmol, loading of 0.91 mmol/g). The reaction mixture was gently agitated at room temperature for 5 h, drained and washed with DMF (4 x 3 mL). A negative Kaiser test[13] confirmed the coupling. The Boc group was then removed by 1 x 2 min treatment with TFA (6 mL) and thoroughly washed with DMF. To this H_2N-Lys(2-Cl-Z)-PAM linker resin was coupled Boc-Lys(2-Cl-Z)-OH (5.0 eq.) four times as part of a penta-lysine tag by Boc *in situ* neutralisation procedure,[14] using 100% TFA as a deblocking reagent and HATU (4.75 eq.)/*i*Pr_2EtN (8.0 eq.) as coupling reagents/base to yield peptidyl-resin **S13**. To the deprotected H_2N-$(Lys)_5$-PAM linker resin **S13** was added a mixture of *S*-trityl-3-mercaptopropionic acid (139.4 mg, 0.4 mmol), HATU (140.7 mg, 0.37 mmol) and *i*Pr_2EtN (139.4 µL, 0.8 mmol) in DMF (3 mL) and the reaction mixture was shaken at room temperature for 30 min.[15] The resulting trityl-3-mercaptopropionic acid-$(Lys)_5$-PAM resin was used as a starting resin for polypeptide chain assembly. Trityl-protecting group was removed by treatment with TFA/*i*Pr_3SiH/H_2O (*v/v/v*; 95:2.5:2.5) for 2 x 5 min to yield **S14**. Elongation of peptide was then performed *via* the Boc *in situ* neutralisation procedure, as previously described, to yield peptidyl-resin **S15**. Upon completion of the peptide assembly, the peptidyl resin was treated with 30 mM Me_2S and 30 mM TBAI in TFA for 2 min to effect a one-step reduction of methionine sulfoxide and Boc deprotection. The resin was then washed with DMF (4 x 8 mL), CH_2Cl_2 (4 x 5 mL), and dried under vacuum. The resulting peptidyl resin was cleaved, with simultaneous removal of side-

chain protecting groups using anhydrous HF/*p*-cresol (*v/v*; 19:1, 10 mL) for 1 h at 0 °C. Following evaporation of HF, the peptide was precipitated with cold Et_2O and isolated by centrifugation (4000 rpm, 6 min). The precipitated product was dissolved in a 1:1 mixture of H_2O and CH_3CN (*v/v*, containing 0.1% TFA) and lyophilised to afford the crude peptide. Purification by RP-HPLC afforded peptide **6**, H_2N-^{1}Lys-^{4}Trp(CHO)-^{6}Trp(CHO)-^{11}Met-$COSCH_2CH_2CO$-$(Lys)_5$-COOH, as an amorphous solid.

Peptide **6** (142 mg, 33% yield, 99% purity); R_t 15.7 min; *m/z* (ESI-MS) 1078.0 ($[M+2H]^{2+}$ calculated: 1078.1). Analysed by XTerra® MS C_{18} column (4.6 x 150 mm; 5 µm), gradient 5-75% B over 35 mins, 1.0 mL/min.

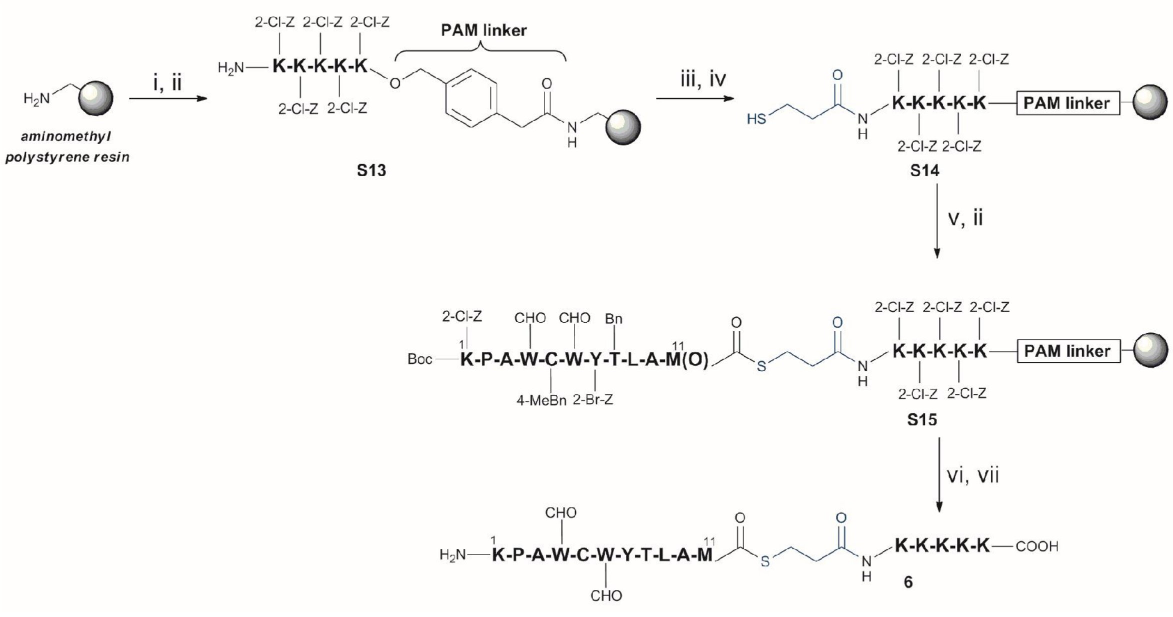

Scheme 2. Synthesis of the thioester fragment **6**. *Reagents and conditions*: (i) Boc-Lys(2-Cl-Z)-PAM, DIC, CH_2Cl_2, r.t, 4 h; (ii) Boc-SPPS (Boc deprotection: 100% TFA, r.t, 2 min; coupling: Boc-amino acid, HATU, *i*Pr_2EtN, DMF, r.t, 5 min; (iii) *S*-trityl-3-mercaptopropionic acid, HATU, *i*Pr_2EtN, DMF, r.t, 30 min; (iv) TFA/*i*Pr_3SiH/H_2O (95:2.5:2.5, *v/v/v*), r.t, 2 x 5 min; (v) Boc-Met(O)-OH, HATU, *i*Pr_2EtN, DMF, r.t, 45 min; (vi) 30 mM Me_2S, 30 mM TBAI, TFA, r.t, 2 min; (vii) HF, *p*-cresol, 0 °C, 1 h.

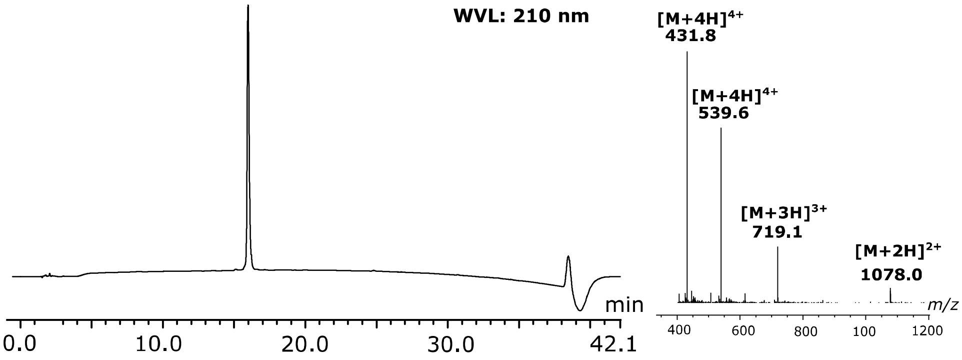

Figure 11. LC-MS chromatogram of purified thioester peptide **6**. Analysed by XTerra® MS C_{18} column (4.6 x 150 mm; 5 µm), gradient 5-75% B over 35 mins, 1.0 mL/min.

4.1.2 Synthesis of peptide 3

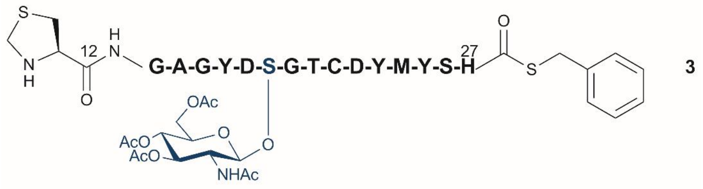

3

Procedure one

A solution of HMPB linker (72.1 mg, 0.3 mmol) and DIC (47 µL, 0.3 mmol) in CH_2Cl_2/DMF (*v/v*; 2:1, 3 mL) was added to pre-swollen (CH_2Cl_2, 3 mL, 20 min) aminomethyl polystyrene resin (110.0 mg, 0.1 mmol, loading 0.91 mmol/g) and the mixture was shaken for 4 h at room temperature, filtered, and washed with DMF (4 x 3 mL). A negative Kaiser test[13] confirmed the coupling. A mixture of Fmoc-His(Trt)-OH (185.9 mg, 0.3 mmol), DIC (47 µL, 0.3 mmol) and DMAP (1.2 mg, 0.01 mmol) in DMF (3 mL) was then added to the resin and the reaction mixture was shaken for 1 h at room temperature to yield Fmoc-His(Trt)-HMPB-resin **S16**. This procedure was repeated once with fresh reagents. Solid phase peptide synthesis was performed on the resulting resin at room temperature using a PS3™ automated Peptide Synthesizer (Protein technologies Inc., Arizona, USA). Deprotection of the Fmoc group was accomplished using 20% piperidine in DMF (3 mL, *v/v*) for 2×5 min with consecutive DMF washes after each addition. Amino acid couplings were performed using a mixture of Fmoc-amino acid (0.4 mmol), HATU (136.9 mg, 0.36 mmol), *i*Pr_2EtN (139.4 µL, 0.8 mmol) in DMF at room temperature for 35 min, except Fmoc-*L*-Ser(β-GlcNAc(OAc)$_3$)-OH **S6** which was coupled using a mixture of Fmoc-amino acid (131.3 mg, 0.2 mmol), HATU (74.1 mg, 0.195 mmol), HOAt (26.5, 0.195 mmol), and 2,4,6-trimethylpyridine (25.8 µL, 0.195 mmol) in DMF at room temperature overnight to yield **S17**. The complete side-chain protected peptide was released from the resin by the treatment of 1% TFA in CH_2Cl_2 (4 mL, *v/v*) for 4 x 5 min followed by removal of the resin by filtration. Solvent from the combined filtrates was evaporated under reduced pressure and the residue was re-suspended in 3:7 mixture of H_2O and CH_3CN (*v/v*, containing 0.1% TFA) and lyophilised to yield the protected precursor **S18**, which was used without further purification.

The crude fully protected peptide **S18** solution in CH_2Cl_2 (20 mL) was reacted with benzyl mercaptan (469.6 µL, 4.0 mmol), DIC (626.3 µL, 4.0 mmol), 6-Cl-HOBt (678.3 mg, 4.0 mmol) and *i*Pr_2EtN (231.7 µL, 1.33 mmol) at room temperature overnight. Solvent was removed by evaporation using a flow of N_2 and the crude product treated with TFA/*i*Pr_3SiH/H_2O/EDT (*v/v/v/v*; 94:1:2.5:2.5, 10 mL) for 2 h for deprotection of side-chain protecting groups. Following evaporation of TFA, the peptide was precipitated with cold Et_2O and isolated by centrifugation (4000 rpm, 6 min). The precipitated product was dissolved in 1:1 mixture of H_2O and CH_3CN (*v/v*, containing 0.1% TFA) and lyophilised to afford the crude peptide. Purification by RP-HPLC afforded peptide **3**, H_2N-^{12}Thz-^{18}Ser(β-GlcNAc(OAc)$_3$)-^{27}His-COSBn, as an amorphous solid.

Glycopeptide **3** (50.1 mg, 23% yield based on 0.1 mmol, 98% purity); R_t 17.3 min; *m/z* (ESI-MS) 1089.0 ($[M+2H]^{2+}$ calculated: 1088.9). Analysed by XTerra® MS C_{18} column (4.6 x 150 mm; 5 µm), gradient 5-75% B over 35 mins, 1.0 mL/min.

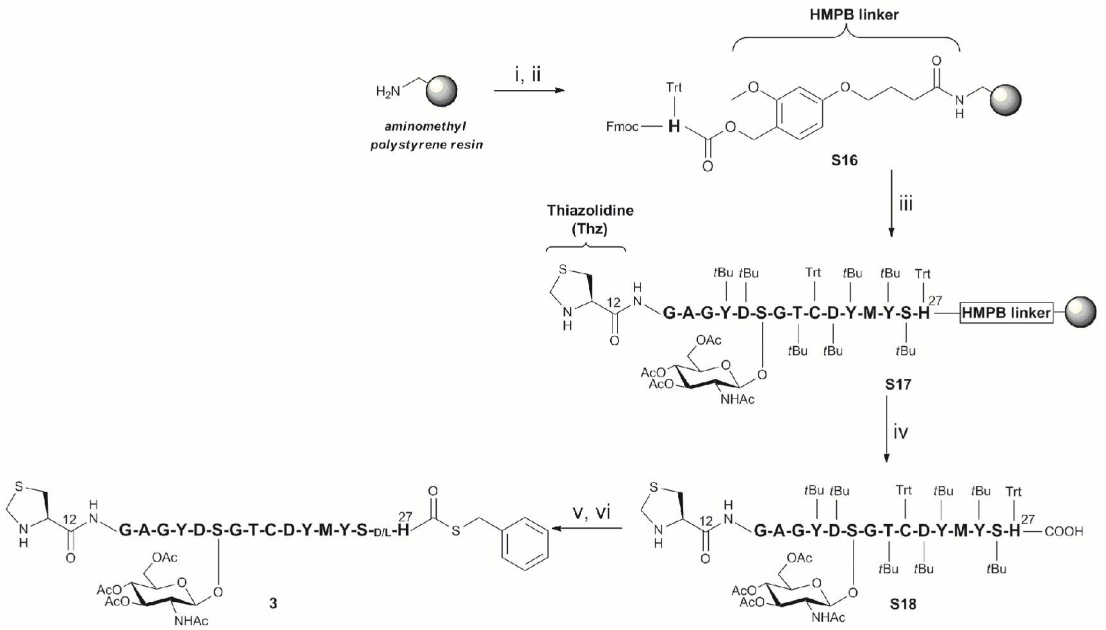

Scheme 3. Synthesis of glycopeptide **3**. *Reagents and conditions*: (i) 4-(4-Hydroxymethyl-3-methoxyphenoxy)butyric acid (HMPB) , DIC, CH_2Cl_2, r.t, 4h; (ii) Fmoc-His(Trt)-OH, DIC, DMAP, DMF, r.t, 2 x 1 h; (iii) Fmoc-SPPS (Fmoc deprotection: 20% piperidine in DMF (*v/v*), r.t, 2 x 5 min; coupling: Fmoc-amino acid (4.0 eq.), HATU (3.6 eq.), *i*Pr_2EtN (8 eq.), DMF, r.t, 40 min (except Fmoc-*L*-Ser(β-GlcNAc(OAc)$_3$)-OH **S6** (2 eq.), HATU (1.95 eq.), HOAt (1.95 eq.), TMP (1.95 eq.), DMF, r.t, overnight); (iv) 1% TFA in CH_2Cl_2 (*v/v*), r.t, 4 x 5 min; (v) benzyl mercaptan (4.0 eq.), DIC (4.0 eq.), 6-Cl-HOBt (4.0 eq.), *i*Pr_2EtN (1.3 eq.), CH_2Cl_2, r.t, overnight; (vi) TFA/EDT/H_2O/*i*Pr_3SiH (94:2.5:2.5:1, *v/v/v/v*), r.t, 2 h.

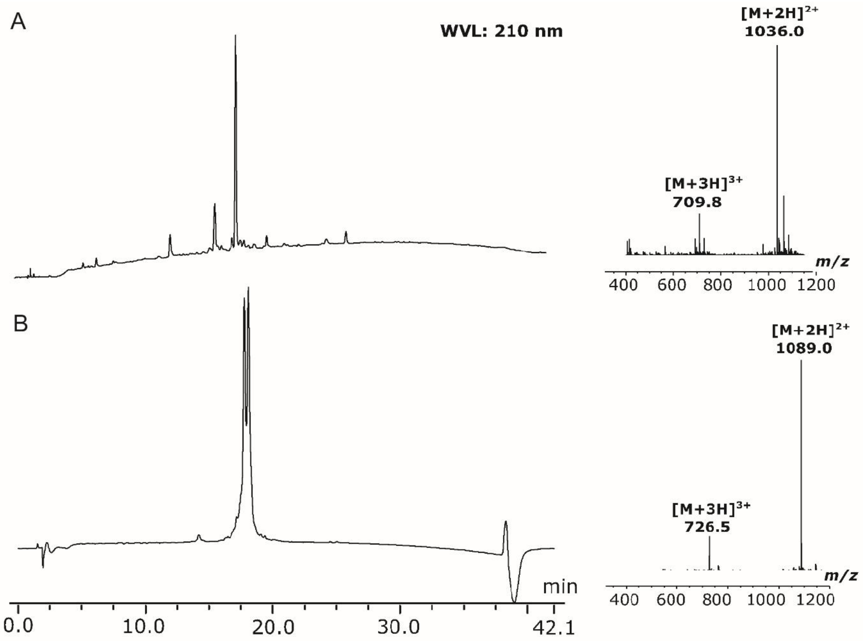

Figure 12. LC-MS chromatograms for the synthesis of the thiazolidine-thioester fragment **3**. (A) Crude TFA-treated peptide **S18**; (B) Purified glycopeptide **3**. Analysed by XTerra® MS C_{18} column (4.6 x 150 mm; 5 µm), gradient 5-75% B over 35 mins, 1.0 mL/min.

Procedure two (optimised Method)

Fmoc-His(Trt)-OH (123.9 mg, 0.2 mmol) was dissolved in CH_2Cl_2 (2 mL) and added to 2-ClTrtCl resin (53 mg, 0.1 mmol, loading of 0.8-3.0 mmol/g). *i*Pr_2EtN (35 µL, 0.2 mmol) was then added and the mixture was shaken at room temperature for 1 h. The resin was then filtered, washed with DMF, and unreacted resin beads were capped by treatment with CH_2Cl_2/CH_3OH/*i*Pr_2EtN (*v/v/v*; 80:15:5) for 2 x 10 min. The resin was then washed with DMF (4 x 4 mL). The remaining sequence was assembled using Fmoc-SPPS, as described in 'procedure one' except for Fmoc-Cys(Trt)-OH (for the coupling of ^{21}Cys) which was coupled using a mixture of Fmoc-amino acid (234.3 mg, 0.4 mmol), HCTU (148.9 mg, 0.36 mmol), HOAt (49 mg, 0.36 mmol), and 2,4,6-trimethylpyridine (106 µL, 0.8 mmol) in CH_2Cl_2/DMF (*v/v*; 1:1) at room temperature for 1 h. The peptidyl-resin **S19** was then suspended in a mixture of TFE/CH_2Cl_2 (*v/v*; 2:8, 5 mL), shaken at room temperature for 1 h, and filtered. The residual solvents were then removed *in vacuo* and the residue was re-suspended in 1:1 mixture of H_2O and CH_3CN (*v/v*, containing 0.1% TFA) and lyophilised to afford side-chain protected precursor **S18**, which was used without further purification.

The crude fully protected peptide **S18** in CH_2Cl_2/DMF (*v/v*; 2:1, 30 mL) was reacted with benzyl mercaptan (408 µL, 3.48 mmol), DIC (539 µL, 3.48 mmol), and 6-Cl-HOBt (590.1 mg, 3.48 mmol) at 4 ^{0}C for 1 h then left at room temperature overnight. Solvent was removed by evaporation using a flow of N_2 and the crude product was precipitated with H_2O, isolated by centrifugation (4000 rpm, 6 min), and lyophilised. The crude side-chain protected peptide was then treated with TFA/*i*Pr_3SiH/H_2O/EDT (*v/v/v/v*; 94:1:2.5:2.5, 10 mL) for 2 h for deprotection of side-chain protecting groups followed by precipitation of the crude peptide using cold Et_2O and isolation by centrifugation (4000 rpm, 6 min). The precipitated product was then dissolved in 1:1 mixture of H_2O and CH_3CN (*v/v*, containing 0.1% TFA) and lyophilised to afford the crude peptide **3** (89.mg, 41% crude yield). Analysed by XTerra® MS C_{18} column (4.6 x 150 mm; 5 µm), gradient 5-75% B over 35 mins, 1.0 mL/min.

Scheme 4. Optimised synthesis of the thiazolidine-thioester fragment **3**. *Reagents and conditions*: (i) Fmoc-His(Trt)-OH (2 eq.), *i*Pr_2EtN (2 eq.), CH_2Cl_2, r.t, 1 h then $CH_2Cl_2/CH_3OH/$*i*Pr_2EtN (*v/v/v*; 80:15:5) for 2 x 10 min; (ii) Fmoc-SPPS (Fmoc deprotection: 20% piperidine in DMF, r.t, 2 x 5 min; coupling: Fmoc-amino acid (4.0 eq.), HATU (3.6 eq.), *i*Pr_2EtN (8.0 eq.), DMF, r.t, 40 min (except Fmoc-*L*-^{18}Ser(β-GlcNAc(OAc)$_3$)-OH **S6** (2.0 eq.), HATU (1.95 eq.), HOAt (1.95 eq.), TMP (1.95 eq.), DMF, r.t, overnight and Fmoc-*L*-^{21}Cys(Trt)-OH **S11** (4.0 eq.), HCTU (3.6 eq.), HOAt (3.6 eq.), TMP (8.0 eq.), CH_2Cl_2/DMF (1:1, *v/v*), r.t, 2 x 1 h); (iii) 20% TFE/CH_2Cl_2 (2:8, *v/v*), r.t, 1 h; (iv) benzyl mercaptan (3.48 eq.), DIC (3.48 eq.), 6-Cl-HOBt (3.48 eq.), CH_2Cl_2/DMF (2:1, *v/v*), 4 °C for 1 h then r.t overnight; (v) TFA/EDT/H_2O/*i*Pr_3SiH (94:2.5:2.5:1, *v/v/v/v*), r.t, 2 h.

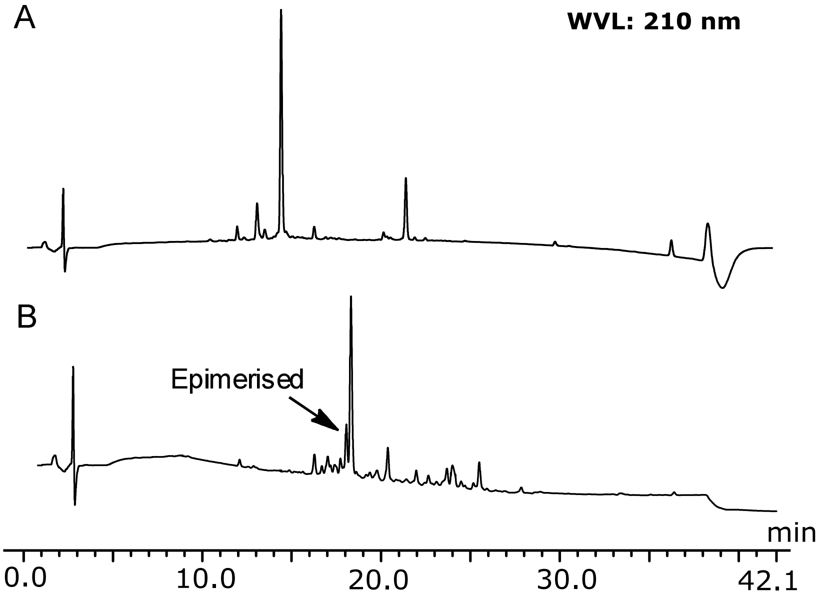

Figure 13. Analytical RP-HPLC chromatograms for the synthesis of peptide **3**. A) prior to activation, B) after activation. Analysed by XTerra® MS C_{18} column (4.6 x 150 mm; 5 μm), gradient 5-75% B over 35 mins, 1.0 mL/min.

4.1.3 Synthesis of peptide 2

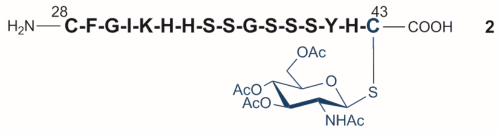

Fmoc-*L*-Cys(β-GlcNAc(OAc)$_3$)-OH **S11** (134.5 mg, 0.2 mmol) was dissolved in CH_2Cl_2 (2 mL) and added to 2-ClTrtCl resin (53 mg, 0.1 mmol, loading of 0.8-3.0 mmol/g) followed by addition of *i*Pr_2EtN (35 µL, 0.2 mmol) which was then shaken at room temperature for 1 h. The resin was then filtered, washed with DMF, and unreacted resin beads were capped by treatment with CH_2Cl_2/CH_3OH/*i*Pr_2EtN (*v/v/v*; 80:15:5) for 2 x 10 min. The remaining sequence was assembled using Fmoc-SPPS, as described in the synthesis of peptide **3**. The resin-bound peptide **S20** was then treated with TFA/*i*Pr_3SiH/H_2O/DODT (*v/v/v/v*; 94:1:2.5:2.5, 10 mL) for 2 h. After work-up and RP-HPLC purification (as previously described), glycopeptide **2**, H_2N-^{28}Cys-^{43}Cys(β-GlcNAc(OAc)$_3$)-COOH, was afforded as an amorphous solid.

Glycopeptide **2** (60 mg, 29% yield, 98% purity); R_t 11.9 min; *m/z* (ESI-MS) 1033.8 ($[M+2H]^{2+}$ calculated: 1033.9). Analysed by XTerra® MS C_{18} column (4.6 x 150 mm; 5 µm), gradient 5-65% B over 30 mins, 1.0 mL/min.

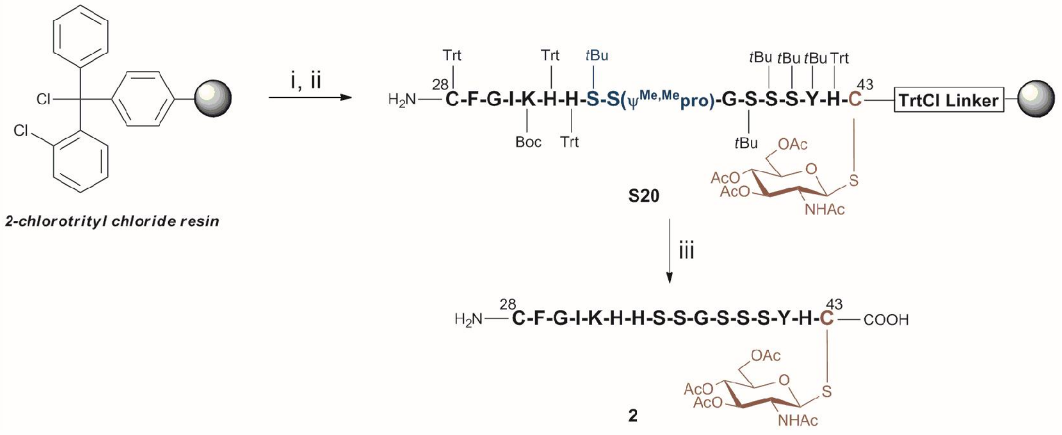

Scheme 5. Synthesis of the cysteinyl peptide **2**. *Reagents and conditions*: (i) Fmoc-*L*-Cys(β-GlcNAc(OAc)$_3$)-OH **S11** (2.0 eq.), *i*Pr_2EtN (2.0 eq.), CH_2Cl_2, r.t, 1 h; (ii) Fmoc-SPPS (Fmoc deprotection: 20% piperidine in DMF (*v/v*), r.t, 2 x 5 min; coupling: Fmoc-amino acid (4.0 eq.), HATU (3.6 eq.), *i*Pr_2EtN (8.0 eq.), DMF, r.t, 40 min; (iii) TFA/DODT/H_2O/*i*Pr_3SiH (94:2.5:2.5:1, *v/v/v/v*), r.t, 2 h.

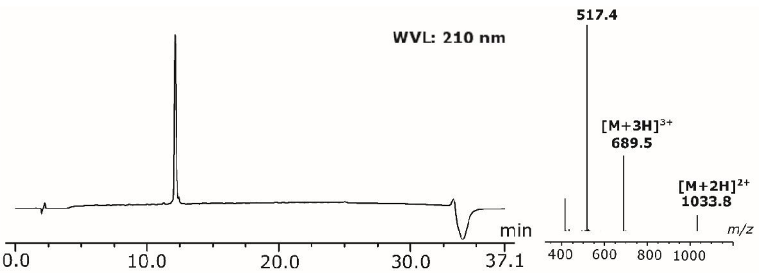

Figure 14. LC-MS chromatogram of purified peptide **2**. Analysed by XTerra® MS C_{18} column (4.6 x 150 mm; 5 µm), gradient 5-65% B over 30 mins, 1.0 mL/min.

4.1.4 Synthesis of polypeptides 5a and 5b

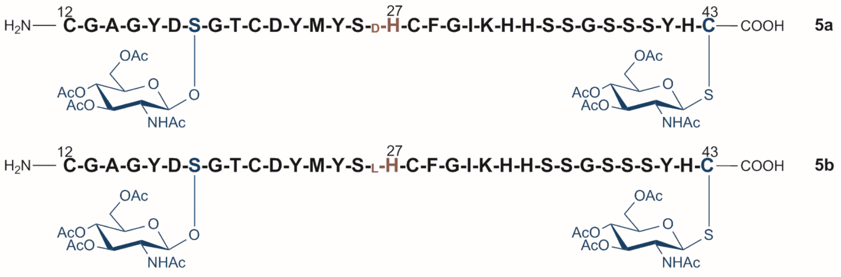

To a degassed solution of 6 M Gn·HCl containing 200 mM Na_2HPO_4 (18.4 mL) was added 100 mM MPAA and 20 mM TCEP. The pH of the resulting solution was adjusted to 6.8 by the addition of 10 M NaOH. To this solution was added peptide **3** (40 mg, 18.4 µmol, final peptide conc. 1 mM) and peptide **2** (38 mg, 18.4 µmol, final peptide conc. 1 mM). The reaction mixture was left at room temperature under argon. Upon completion of the ligation which yielded **4**, as judged by LC-MS of the reaction mixture, the pH of the reaction mixture was adjusted to 4.0 by the addition of 5 M HCl. To effect Thz unmasking, 200 mM methoxyamine·HCl was added, and the reaction mixture left at room temperature for 16 h. Purification by RP-HPLC afforded peptide **5a**, H_2N-^{12}Cys-^{18}Ser(β-GlcNAc(OAc)$_3$)-*D*-^{27}His-^{43}Cys(β-GlcNAc(OAc)$_3$)-COOH and peptide **5b**, H_2N-^{12}Cys-^{18}Ser(β-GlcNAc(OAc)$_3$)-*L*-^{27}His-^{43}Cys(β-GlcNAc(OAc)$_3$)-COOH as amorphous solids.

Glycopeptide **5a** (15 mg, 20% yield, 98% purity); R_t 14.7 min; *m/z* (ESI-MS) 1369.8 ($[M+3H]^{3+}$ calculated: 1369.9). Analysed using a linear gradient of 5%B to 75%B over 35 min, *ca* 2%B/min.

Glycopeptide **5b** (16.5 mg, 22% yield, 98% purity); R_t 15.2 min; *m/z* (ESI-MS) 1369.8 ($[M+3H]^{3+}$ calculated: 1369.9). Analysed using a linear gradient of 5%B to 75%B over 35 min, *ca* 2%B/min.

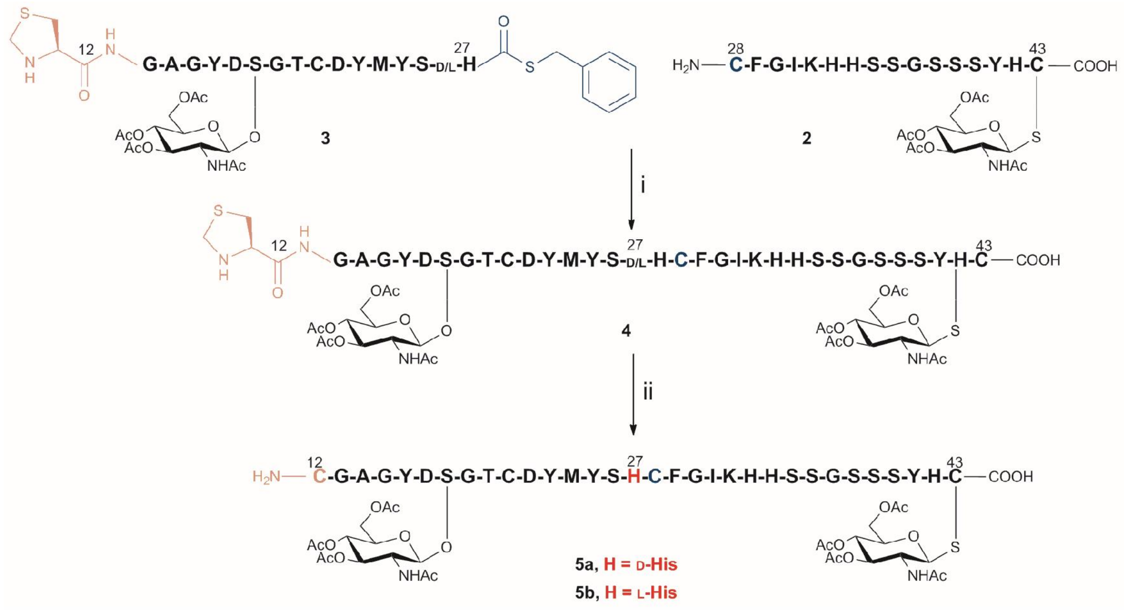

Scheme 6. Synthesis of glycopeptides **5a** and **5b**. *Reagents and conditions*: (i) 6 M Gn.HCl, 0.2 M Na_2HPO_4, 100 mM MPAA, 20 mM TCEP, pH 6.8, r.t, 2 h; (ii) methoxyamine·HCl, pH 4, r.t, 16 h.

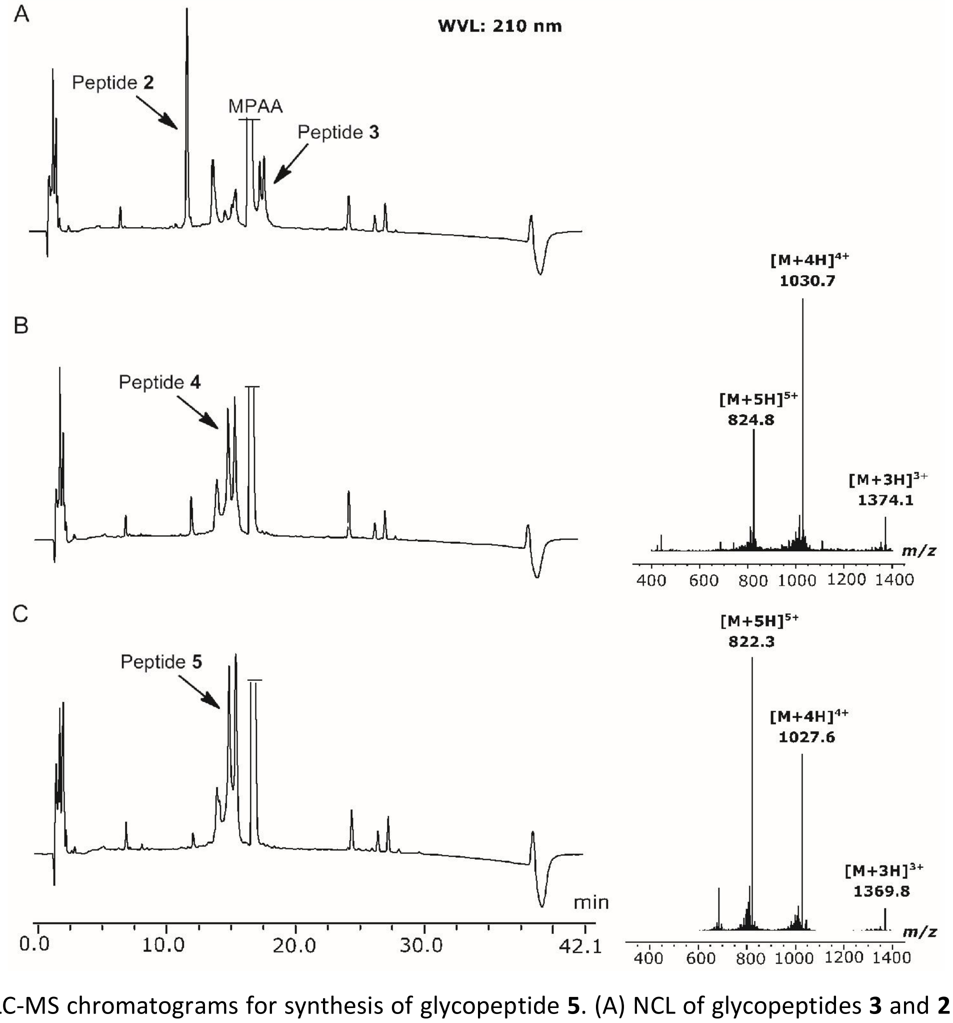

Figure 15. LC-MS chromatograms for synthesis of glycopeptide **5**. (A) NCL of glycopeptides **3** and **2** at t=0 min; (B) Generation of glycopeptide **4** at t = 2 h. (C) Thz to Cys conversion reaction to make glycopeptide **5** after 16 h. Analysed by XTerra® MS C_{18} column (4.6 x 150 mm; 5 µm), gradient 5-75% B over 35 mins, 1.0 mL/min.

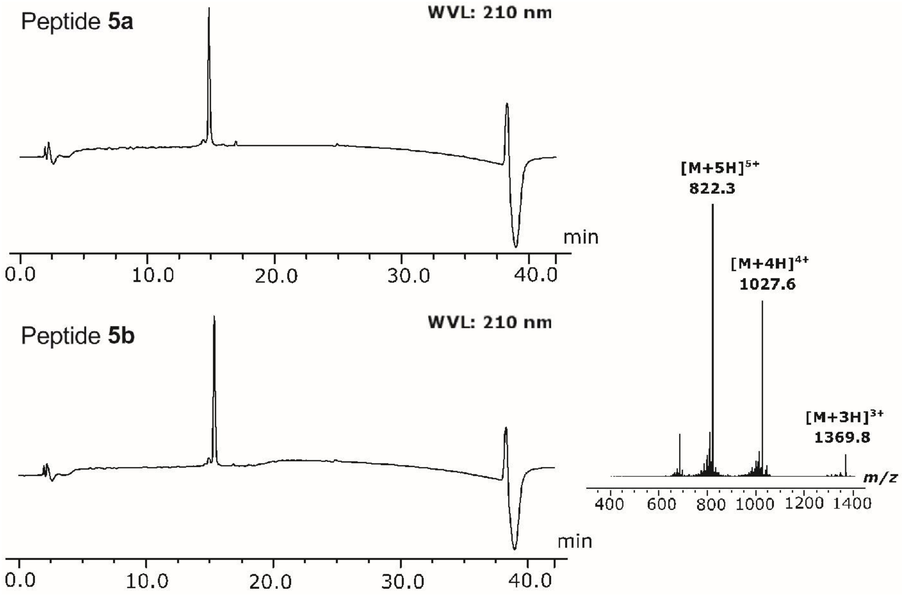

Figure 16. LC-MS chromatograms of purified peptides **5a** and **5b**. Analysed using a linear gradient of 5%B to 75%B over 35 min, *ca* 2%B/min.

4.1.5 Synthesis of polypeptides 7a and 7b

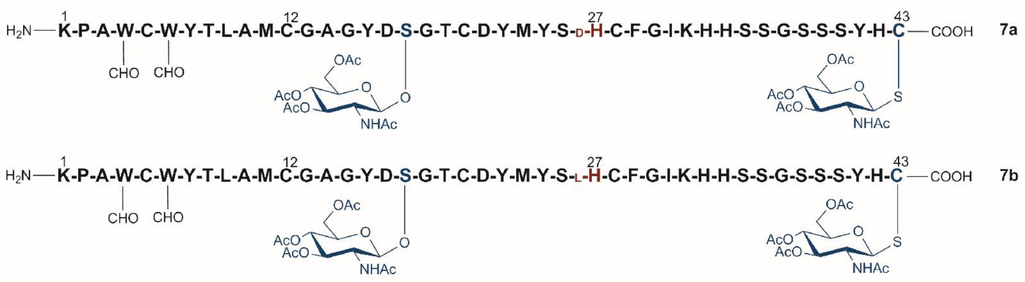

To a degassed solution of 6 M Gn·HCl containing 200 mM Na_2HPO_4 (4.87 mL) was added 100 mM MPAA and 20 mM TCEP. The pH of the resulting solution was adjusted to 6.8 by the addition of 10 M NaOH. To this solution was added peptide **5a** or **5b** (13 mg, 3.17 µmol, final peptide conc. 1 mM) and peptide **6** (5.8 mg, 3.17 µmol, final peptide conc. 1 mM). The reaction mixture was left at room temperature under argon. Upon completion of the ligation, as judged by LC-MS of the reaction mixture, the pH of the reaction mixture was adjusted to 4.0 by the addition of 5 M aq. HCl. The product recovered by solid phase extraction and lyophilised to afford peptide **7a**, H_2N-^{1}Lys-^{4}Trp(CHO)-^{6}Trp(CHO)-^{18}Ser(β-GlcNAc(OAc)$_3$)-*D*-^{27}His-^{43}Cys(β-GlcNAc(OAc)$_3$)-COOH, and peptide **7b**, H_2N-^{1}Lys-^{4}Trp(CHO)-^{6}Trp(CHO)-^{18}Ser(β-GlcNAc(OAc)$_3$)-*L*-^{27}His-^{43}Cys(β-GlcNAc(OAc)$_3$)-COOH as amorphous solids, which were used in the next step without further purification.

Glycopeptide **7a** (10.8 mg, 62% crude yield); R_t 17.8 min; *m/z* (ESI-MS) 1379.7 ($[M+4H]^{4+}$ calculated: 1379.7). Analysed using a linear gradient of 5%B to 75%B over 35 min, *ca* 2%B/min.

Glycopeptide **7b** (11.4 mg, 65% crude yield), R_t 18.4 min; *m/z* (ESI-MS) 1379.7 ($[M+4H]^{4+}$ calculated: 1379.7). Analysed using a linear gradient of 5%B to 75%B over 35 min, *ca* 2%B/min.

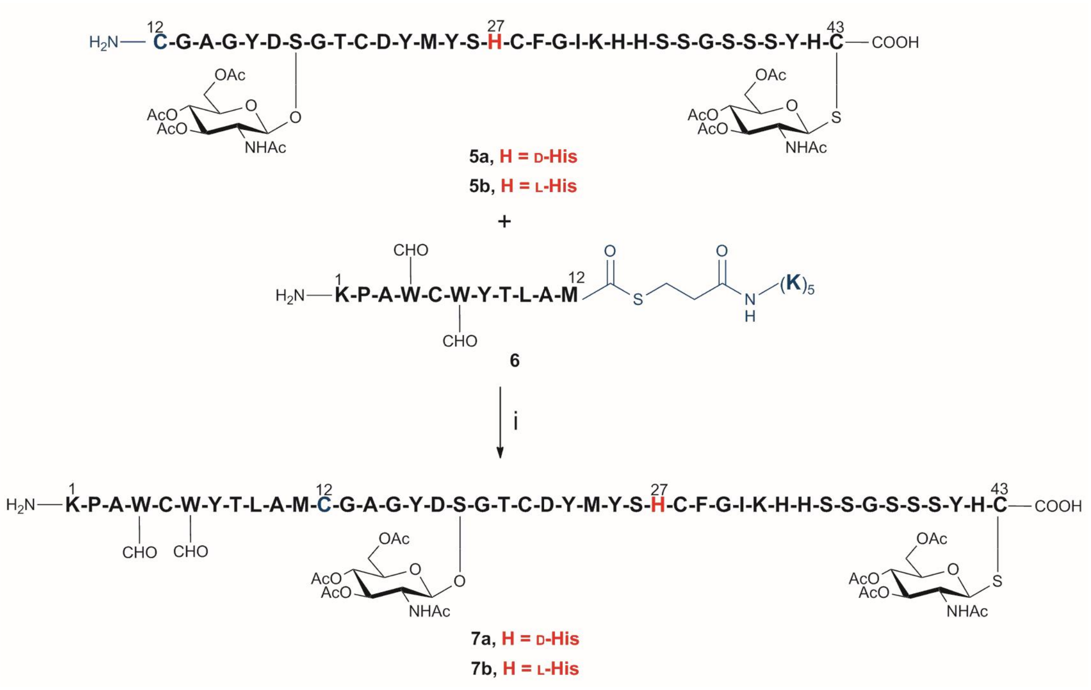

Scheme 7. Synthesis of glycopeptides **7a** and **7b**. *Reagents and conditions*: (i) 6 M Gn.HCl, 0.2 M Na_2HPO_4, 100 mM MPAA, 20 mM TCEP, pH 6.8, r.t, 4 h.

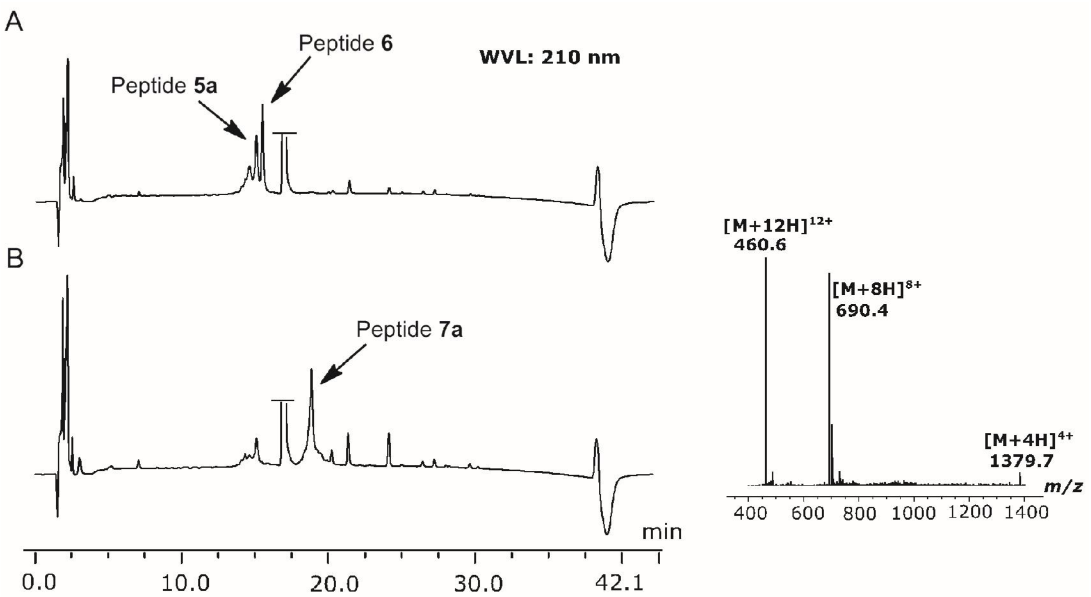

Figure 17. LC-MS chromatograms for synthesis of glycopeptide **7a**. (A) NCL of glycopeptides **6** and **5a** at t=0 min; (B) Generation of the desired glycopeptide at t = 4 h. Analysed by XTerra® MS C_{18} column (4.6 x 150 mm; 5 µm), gradient 5-75% B over 35 mins, 1.0 mL/min.

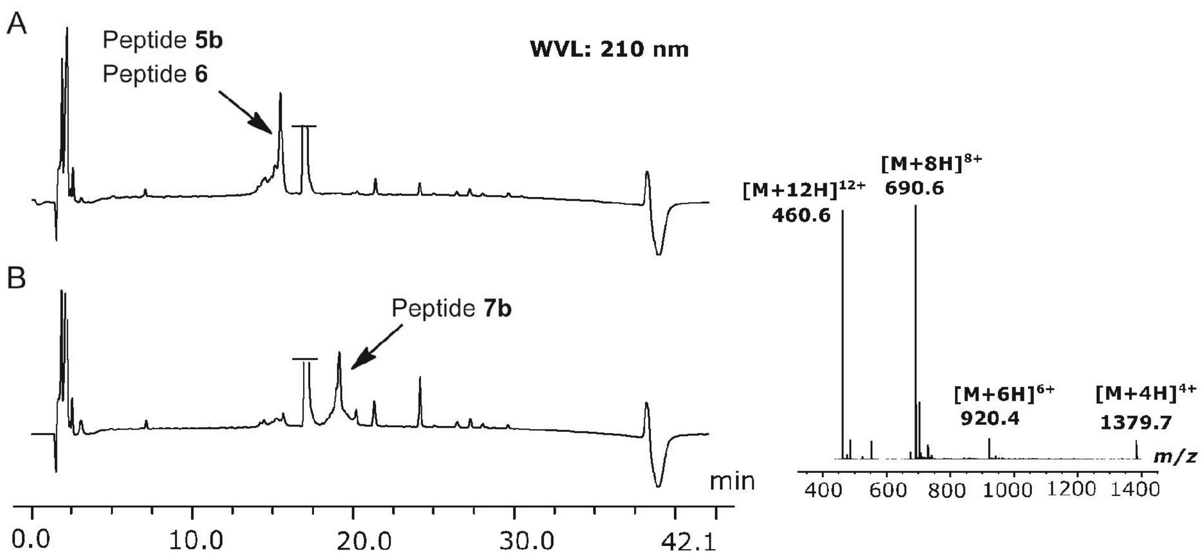

Figure 18. LC-MS chromatograms for synthesis of glycopeptide **7b**. (A) NCL of glycopeptides **6** and **5b** at t=0 min; (B) Generation of the desired glycopeptide at t = 4 h. Analysed by XTerra® MS C_{18} column (4.6 x 150 mm; 5 µm), gradient 5-75% B over 35 mins, 1.0 mL/min.

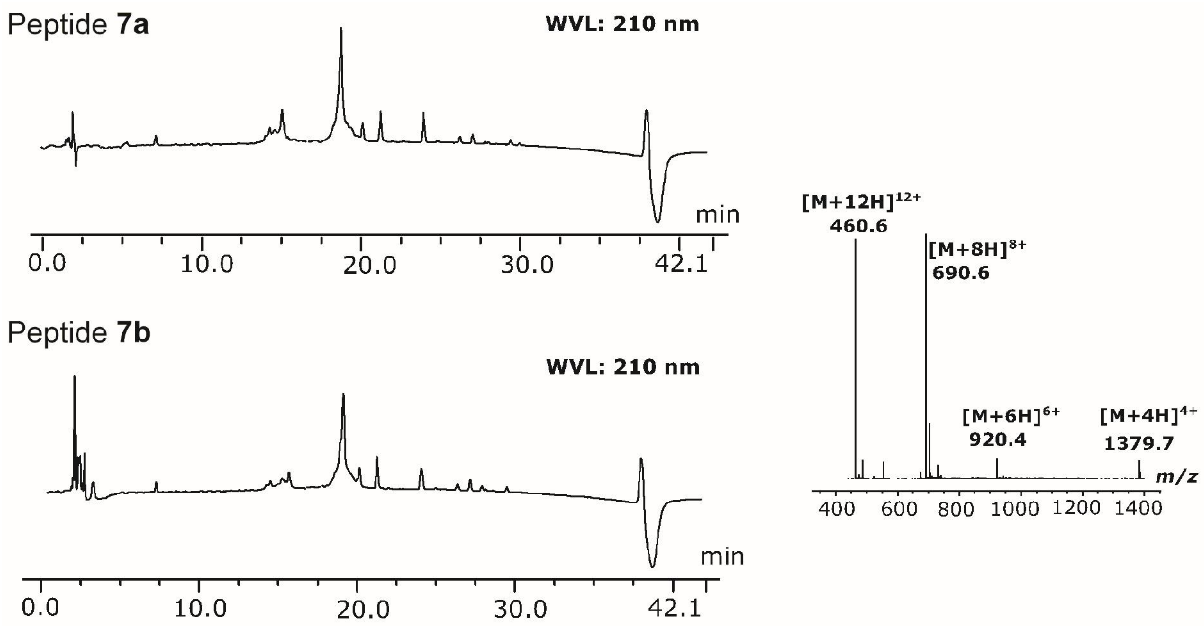

Figure 19. LC-MS chromatograms of purified peptides **7a** and **7b**. Analysed using a linear gradient of 5%B to 75%B over 35 min, *ca* 2%B/min.

4.1.6 Synthesis of polypeptides 8a and 8b

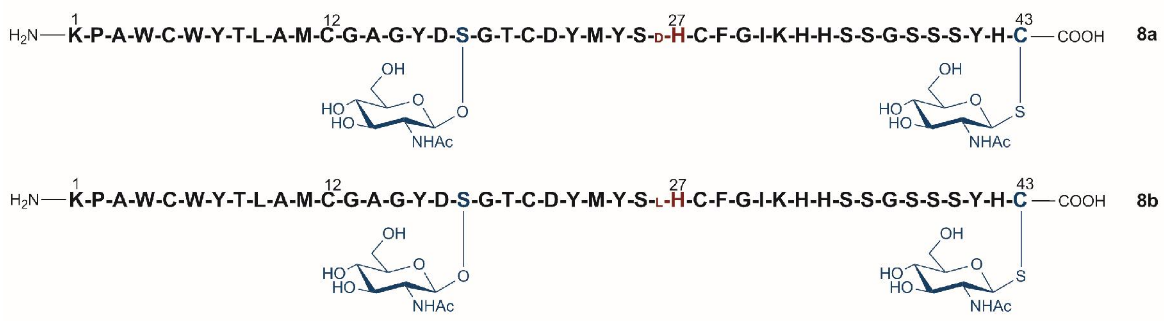

To a mixture of NMP, 6 M Gn·HCl and 1.0 M HEPES (*v/v/v*; 22.5:5.6:9.4, 0.68 mL) was added the crude polypeptide **7a** or **7b** (10 mg, 1.81 µmol). To this solution, a pre-cooled solution of hydrazine with 2-mercaptoethanol (*v/v*; 25:37.5, 1.13 mL) was added and the reaction mixture was left at 0 °C (final peptide conc. 1 mM). Upon completion of the reaction, as judged by LC-MS of the reaction mixture, the product was recovered by solid phase extraction and lyophilised. The reaction afforded epimeric polypeptide **8a**, H_2N-^{1}Lys-^{18}Ser(β-GlcNAc)-*D*-^{27}His-^{43}Cys(β-GlcNAc)-COOH and polypeptide **8b**, H_2N-^{1}Lys-^{18}Ser(β-GlcNAc)-*L*-^{27}His-^{43}Cys(β-GlcNAc)-COOH as amorphous solids, where were used in the next step without any further purification.

Glycopeptide **8a** (4.9 mg, 52% crude yield); R_t 17.2 min; *m/z* (ESI-MS) 1301.2 ($[M+4H]^{4+}$ calculated: 1302.6). Analysed using a linear gradient of 5%B to 75%B over 35 min, *ca* 2%B/min.

Glycopeptide **8b** (5.4 mg, 57% crude yield); R_t 17.5 min; *m/z* (ESI-MS) 1301.6 ($[M+4H]^{4+}$ calculated: 1302.6). Analysed using a linear gradient of 5%B to 75%B over 35 min, *ca* 2%B/min.

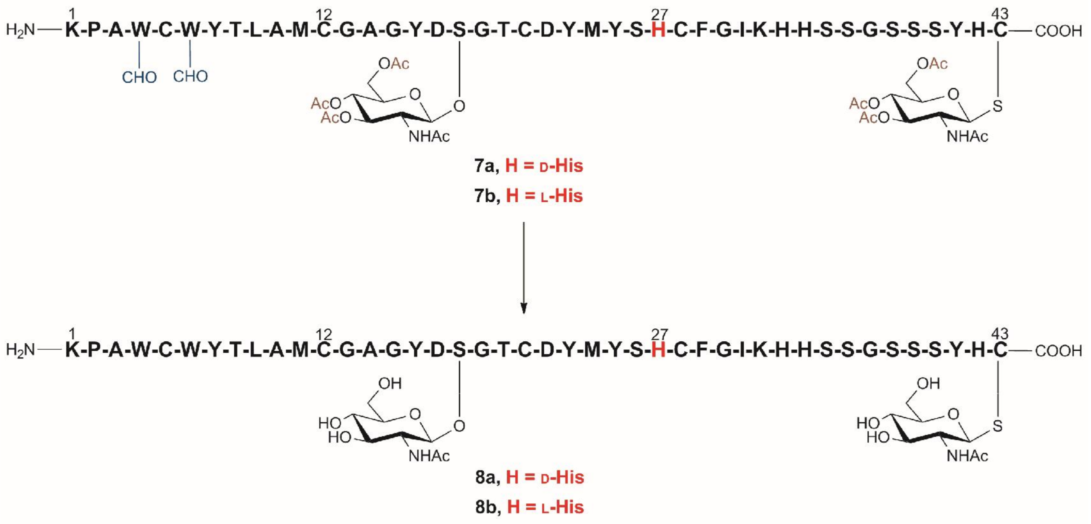

Scheme 8. Global deprotection of acetyl and formyl groups to yield the glycopeptides **8a** and **8b**. *Reagents and conditions*: 6 M Gn.HCl, 1 M HEPES, hydrazine, 2-mercaptoethanol, 0 °C, 30 min.

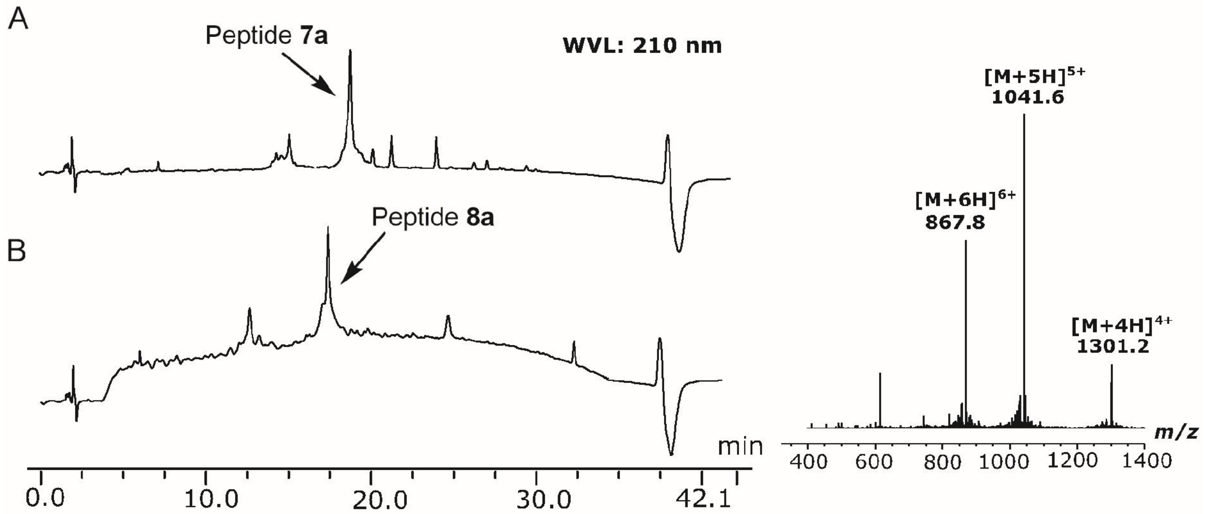

Figure 20. LC-MS chromatograms for synthesis of glycopeptide **8a**. (A) The deprotection reaction at t=0; (B) Generation of the desired glycopeptide at t=30 min. Analysed by XTerra® MS C_{18} column (4.6 x 150 mm; 5 µm), gradient 5-75% B over 35 mins, 1.0 mL/min.

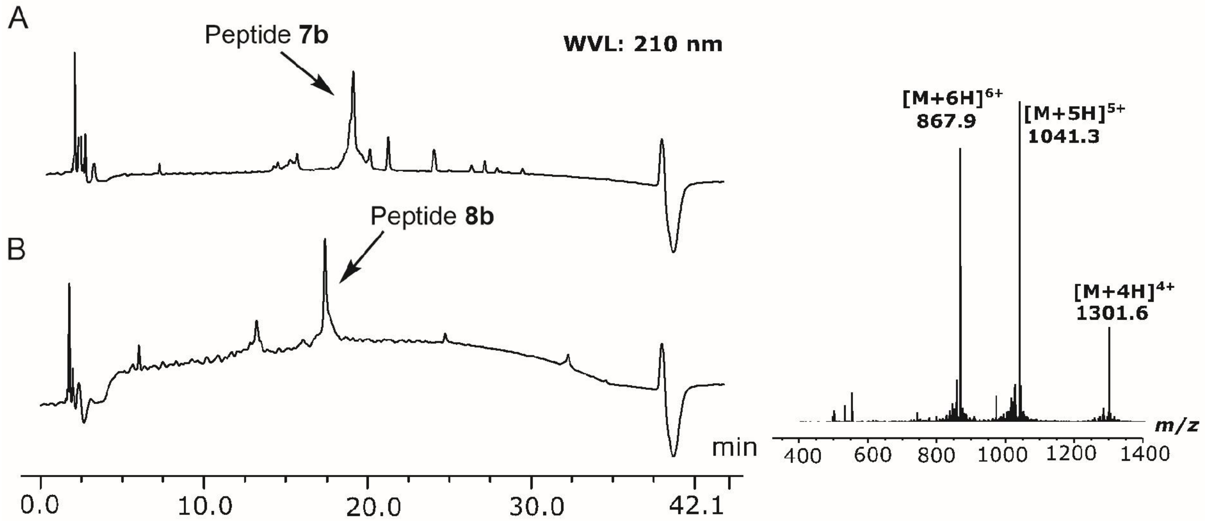

Figure 21. LC-MS chromatograms for the synthesis of glycopeptide **8b**. (A) The deprotection reaction at t=0; (B) Generation of the desired glycopeptide at t=30 min. Analysed by XTerra® MS C_{18} column (4.6 x 150 mm; 5 µm), gradient 5-75% B over 35 mins, 1.0 mL/min.

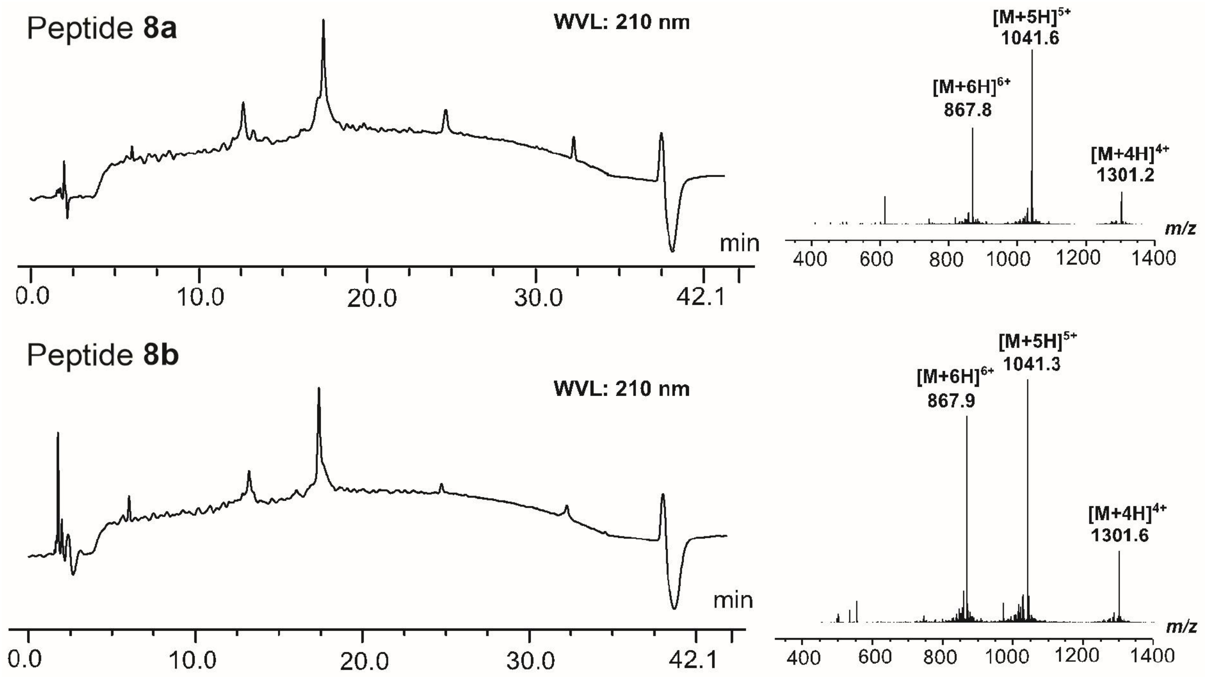

Figure 22. LC-MS chromatograms of peptides **8a** and **8b**. Analysed using a linear gradient of 5%B to 75%B over 35 min, *ca* 2%B/min.

4.1.7 Synthesis of *epi*-(D-[27]His)-GccF 1a and native GccF 1b

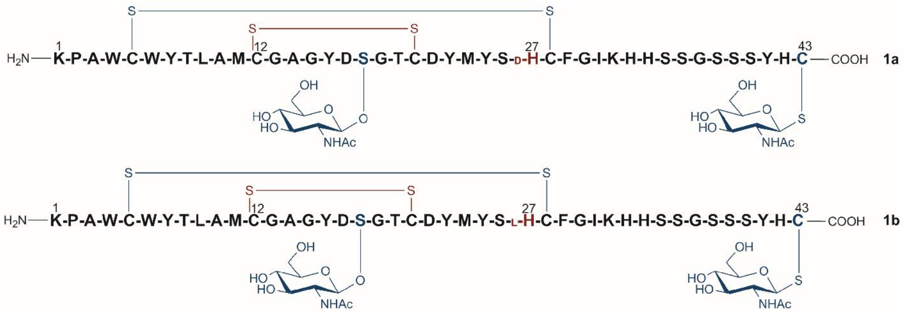

8 mM cysteine, 1 mM cystine and 0.1 mM EDTA were dissolved in a buffer solution containing 6 M Gn·HCl and 200 mM Na_2HPO_4 and the pH adjusted to 8.2 by the addition of 10 M NaOH. The crude polypeptide **8a** or **8b** (4 mg, 0.77 µmol) was dissolved to a final peptide concentration of 1 mM. Peptide was dissolved by sonication, then diluted to 0.25 mM final peptide concentration (*ca*. 1.5 M Gn·HCl, 50 mM Na_2HPO_4, 2 mM cysteine, 0.25 mM cystine, 0.025 mM EDTA) by the addition of Milli-Q water. The resultant mixture was stored at 4 °C until completion of the reaction as judged by LC-MS. Purification by RP-HPLC afforded **1a**, native GccF, and **1b**, *epi*-(D-[27]His)-GccF as amorphous solids.

epi-(D-[27]His)-GccF **1a** (1.1 mg, 29% yield, 99% purity); R_t 17.3 min; *m/z* (HRESI-MS) 1040.8203 ($[M+5H]^{5+}$ requires for $C_{226}H_{316}N_{57}O_{72}S_7$: 1041.4634). Analysed by XTerra® MS C_{18} column (4.6 x 150 mm; 5 µm), gradient 5-75% B over 35 mins, 1.0 mL/min.

GccF **1b** (1.3 mg, 32% yield, 99% purity); R_t 17.5 min; *m/z* (HRESI-MS) 1040.8203 ($[M+5H]^{5+}$ requires for $C_{226}H_{316}N_{57}O_{72}S_7$: 1041.4634). Analysed by XTerra® MS C_{18} column (4.6 x 150 mm; 5 µm), gradient 5-75% B over 35 mins, 1.0 mL/min.

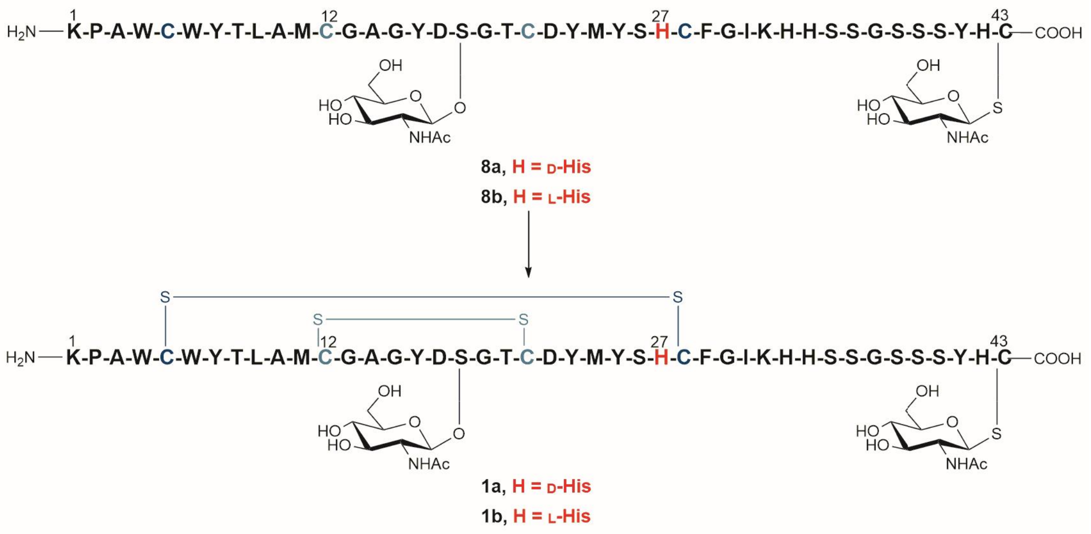

Scheme 9. The final oxidative folding reaction to synthesise *epi*-(*D*-^{27}His)-GccF **1a** and native GccF **1b**. *Reagents and conditions*: 1.5 M Gn·HCl, 50 mM Na_2HPO_4, 2 mM cysteine, 0.25 mM cystine, 0.025 mM EDTA, pH 8.2, 0.25 mM peptide, 4 ^{o}C, 16h.

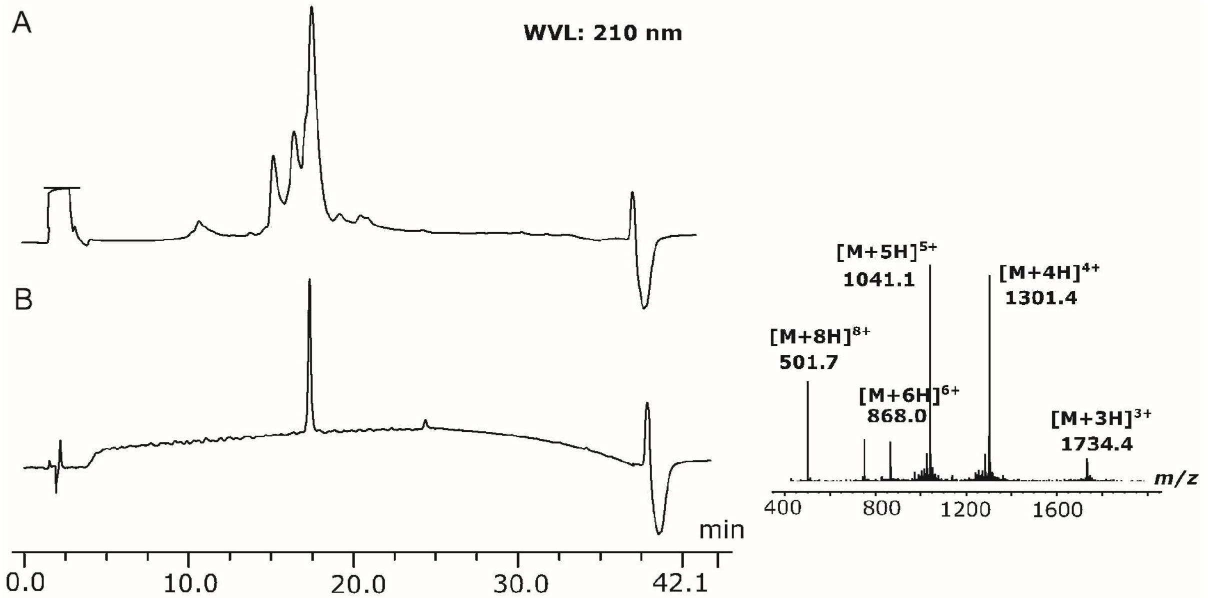

Figure 23. LC-MS chromatograms for the folding reaction. The folding reaction of peptide **8a** to generate *epi*-(*D*-^{27}His)-GccF **1a**. (A) crude profile of the overnight folding reaction; (B) the purified *epi*-(*D*-^{27}His)-GccF **1a**. Analysed by XTerra® MS C_{18} column (4.6 x 150 mm; 5 µm), gradient 5-75% B over 35 mins, 1.0 mL/min.

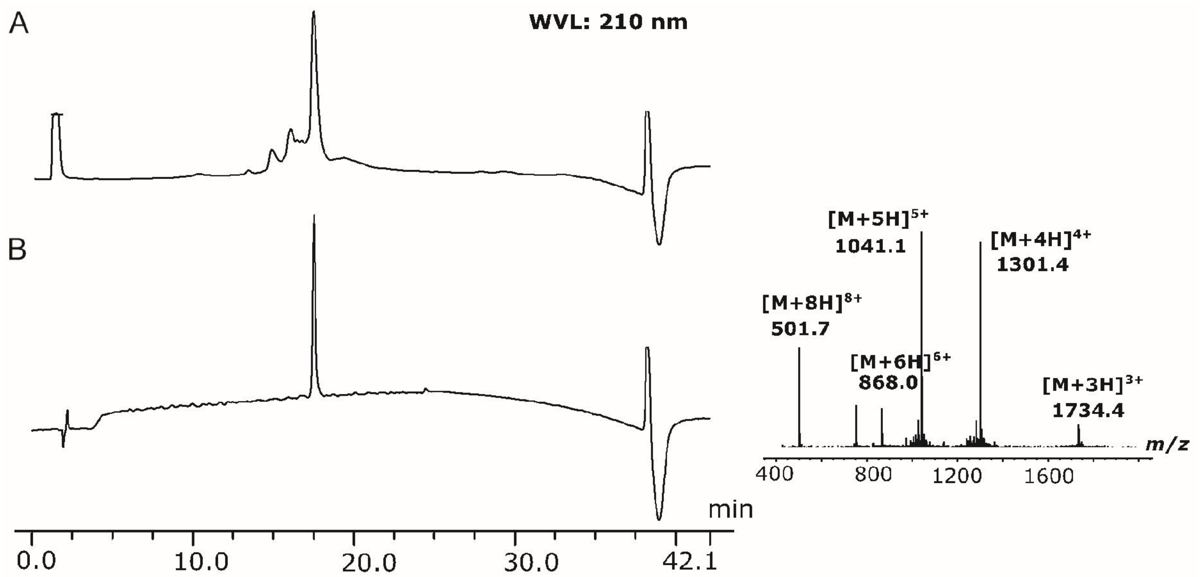

Figure 24. LC-MS chromatograms for the folding reaction. The folding reaction of peptide **8b** to generate native GccF **1b**. (A) crude profile of the overnight folding reaction; (B) the purified native GccF **1b**. Analysed by XTerra® MS C_{18} column (4.6 x 150 mm; 5 µm), gradient 5-75% B over 35 mins, 1.0 mL/min.

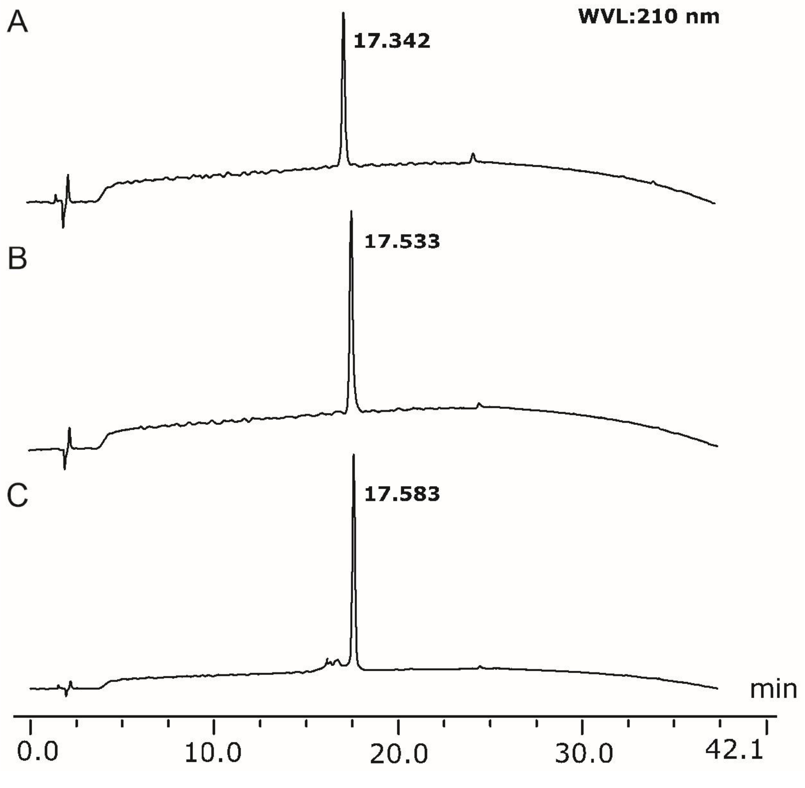

Figure 25. Analytical RP-HPLC chromatograms of: (A) synthetic *epi*-(*D*-^{27}His)-GccF **1a**; (B) synthetic native GccF **1b**; (C) isolated GccF from *Lactobacillus plantarum*. Analysed by XTerra® MS C_{18} column (4.6 x 150 mm; 5 µm), gradient 5-75% B over 35 mins, 1.0 mL/min.

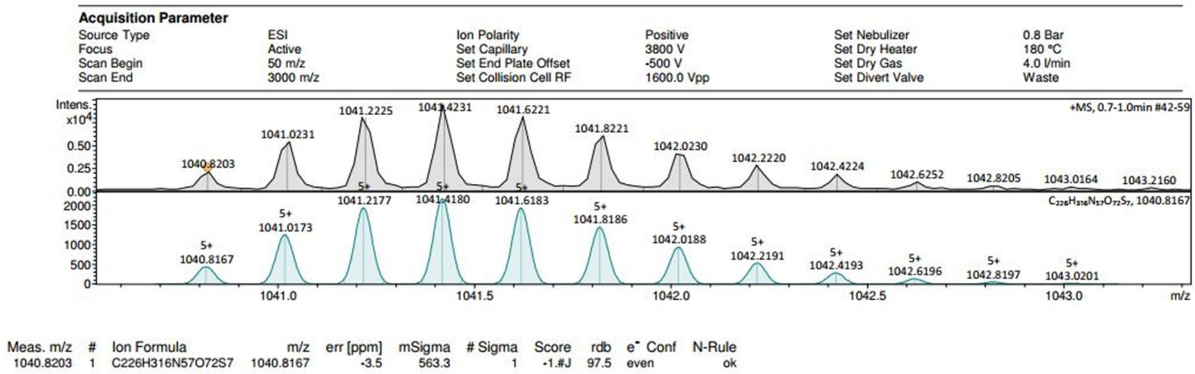

Figure 26. HRESI-MS analysis of synthetic native GccF **1a**.

4.2 Optimised synthesis of fragment H_2N-[12]Gly-[27]His-COOH, by Fmoc-SPPS

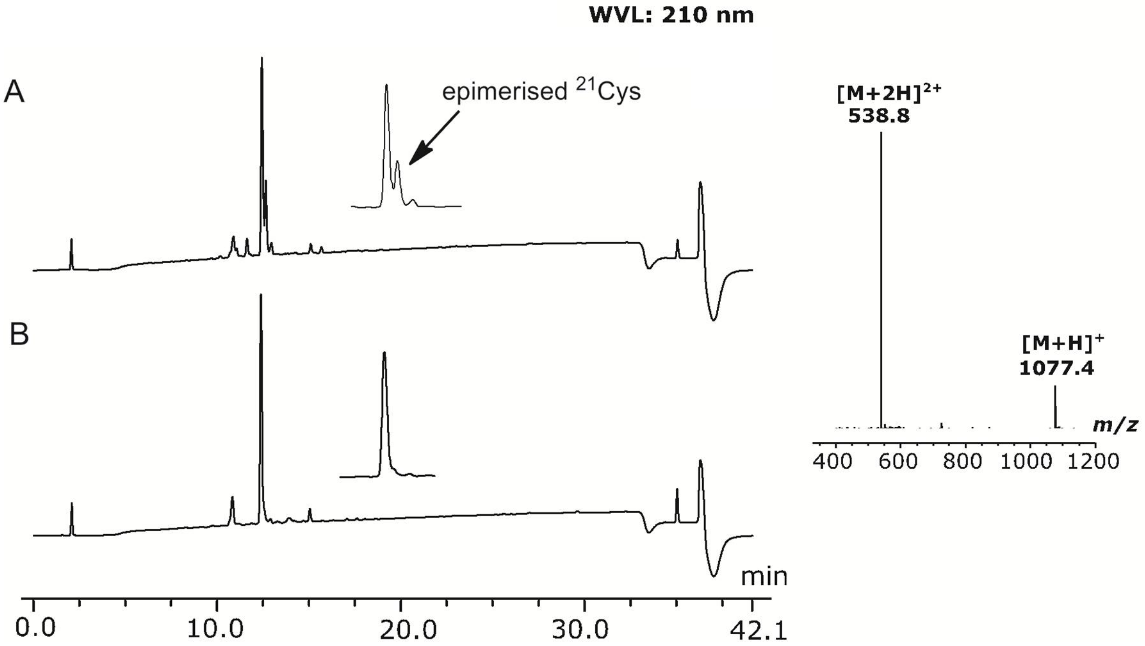

Figure 27. LC-MS chromatograms of H_2N-^{12}Gly-^{27}His-COOH. A) peptide fragment synthesized using 2-ClTrtCl-based resin, B) Repeated synthesis of the same peptide fragment using epimerisation-suppression conditions for the coupling of ^{21}Cys. Analysed using a gradient of 1-61% B over 30 mins, 1.0 mL/min.

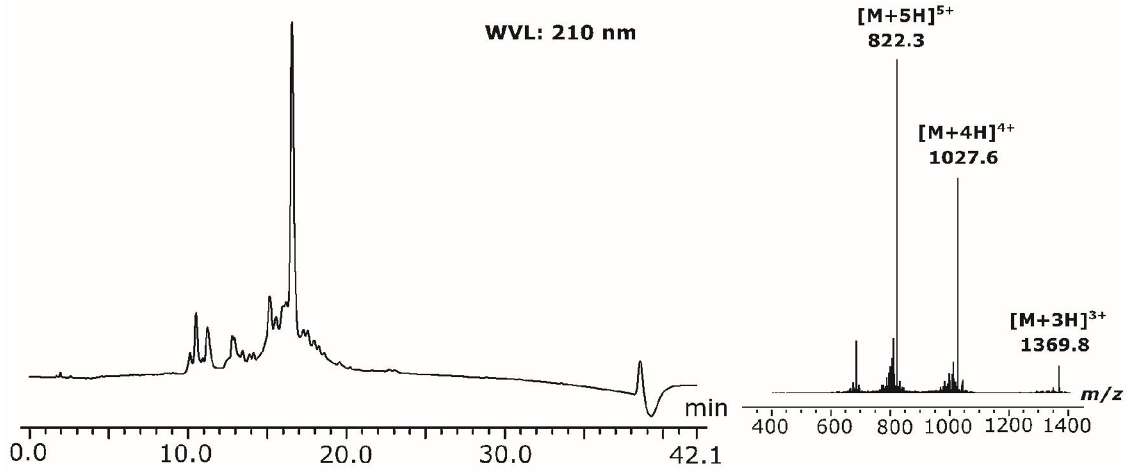

Figure 28. LC-MS chromatogram for the optimised direct synthesis of peptide **5**. Analysed using a gradient of 5-75% B over 35 mins, 1.0 mL/min.

4.3 Synthesis of 'Glyco-mutant' of GccF 10 and 11

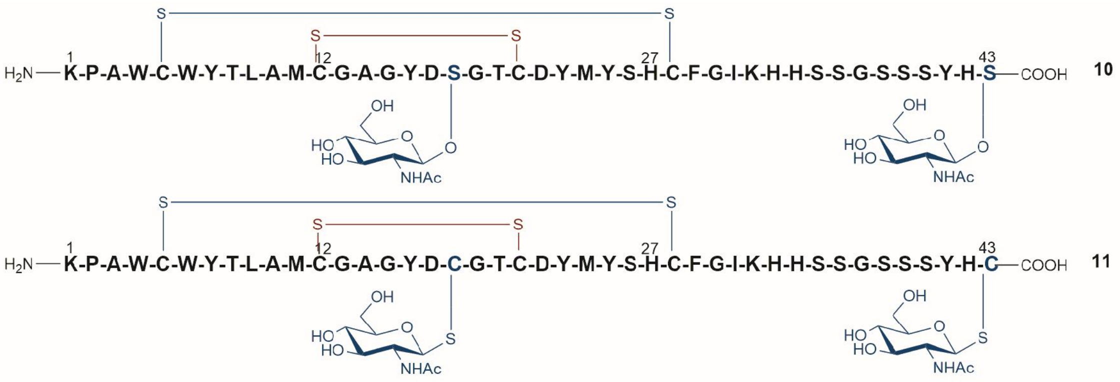

4.3.1 Direct synthesis of peptides 14 and 15

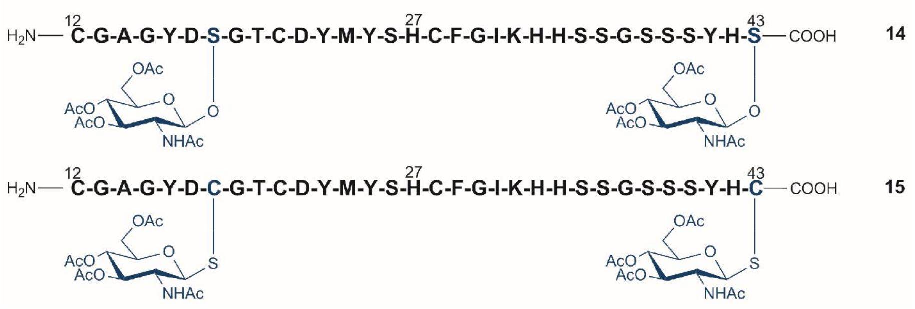

Fmoc-*L*-Ser(β-GlcNAc(OAc)$_3$)-OH **S6** (for peptide **14**) or Fmoc-*L*-Cys(β-GlcNAc(OAc)$_3$)-OH **S11** (for peptide **15**) were dissolved separately in CH_2Cl_2 (2 mL) and added separately to 2-ClTrtCl resin (53 mg, 0.1 mmol, loading of 0.8-3.0 mmol/g). *i*Pr$_2$EtN (35 µL, 0.2 mmol) was then added to the separate mixtures and the reactions were shaken at room temperature for 1 h. The resins were then filtered, washed with DMF, and unreacted resin were capped by treatment with CH_2Cl_2/CH_3OH/*i*Pr$_2$EtN (*v/v/v*; 80:15:5) for 2 x 10 min.

The remaining sequences were assembled *via* Fmoc-SPPS, using the same conditions described previously in the optimised synthesis of peptide **3** (Section 4.1.2, Procedure two). Fmoc-Gly-Thr(ψ^{Me,Me}pro)-OH, Fmoc-Tyr(*t*Bu)-Ser(ψ^{Me,Me}pro)-OH, and Fmoc-Ser(*t*Bu)-Ser(ψ^{Me,Me}pro)-OH were also coupled in their respective positions as per the conditions described in Section 4.1.2.

The resin-bound peptides **12** and **13** were then treated separately with TFA/*i*Pr$_3$SiH/H_2O/EDT (*v/v/v/v*; 94:1:2.5:2.5, 10 mL) for 2 h. After work-up and RP-HPLC purification, glycopeptide **14**, H$_2$N-^{12}Cys-^{18}Ser(β-GlcNAc(OAc)$_3$)-^{43}Ser(β-GlcNAc(OAc)$_3$)-COOH and **15**, H$_2$N-^{12}Cys-^{18}Cys(β-GlcNAc(OAc)$_3$)-^{43}Cys(β-GlcNAc(OAc)$_3$)-COOH, were afforded as amorphous solids.

Glycopeptide **14** (70 mg, 17% yield, 98% purity); R_t 15.9 min; *m/z* (ESI-MS) 1364.5 ([M+3H]$^{3+}$ calculated: 1364.6). (Analysed using a linear gradient of 5%B to 75%B over 35 min, *ca* 2%B/min).

Glycopeptide **15** (57.7 mg, 14% yield, 98% purity); R_t 15.8 min; *m/z* (ESI-MS) 1375.3 ($[M+3H]^{3+}$ calculated: 1375.3). (Analysed using a linear gradient of 5%B to 65%B over 30 min, *ca* 2%B/min).

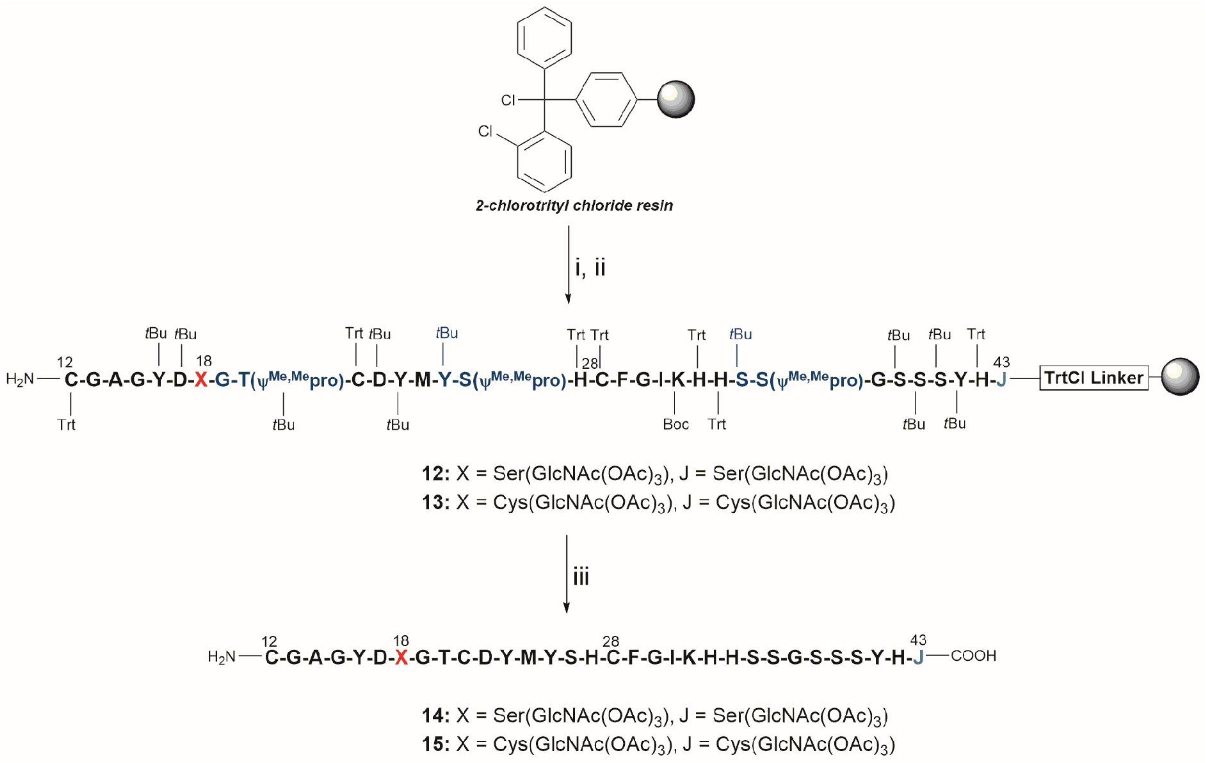

Scheme 10. Synthesis of peptide fragments **14** and **15**. *Reagents and conditions*: (i) Fmoc-*L*-Cys(β-GlcNAc(OAc)$_3$)-OH **S11** or Fmoc-*L*-Ser(β-GlcNAc(OAc)$_3$)-OH **S6**, *i*Pr$_2$EtN, CH_2Cl_2, r.t, 1 h; (ii) Fmoc-SPPS (Fmoc deprotection: 20% piperidine in DMF (*v*/*v*), r.t, 2 x 5 min; coupling: Fmoc-amino acid (4.0 eq.), HATU (3.6 eq.), *i*Pr$_2$EtN (8.0 eq.), DMF, r.t, 40 min (except Fmoc-*L*-^{18}Ser(β-GlcNAc(OAc)$_3$)-OH **S6** (2.0 eq.), HATU (1.95 eq.), HOAt (1.95 eq.), TMP (1.95 eq.), DMF, r.t, overnight and Fmoc-^{21}Cys(Trt)-OH (4.0 eq.), HATU (3.6 eq.), HOAt (3.6 eq.), TMP (8.0 eq.), CH_2Cl_2/DMF (1:1, *v*/*v*), r.t, 2 x 1 h); (iii) TFA/EDT/H_2O/*i*Pr$_3$SiH (94:2.5:2.5:1, *v*/*v*/*v*/*v*), r.t, 2 h.

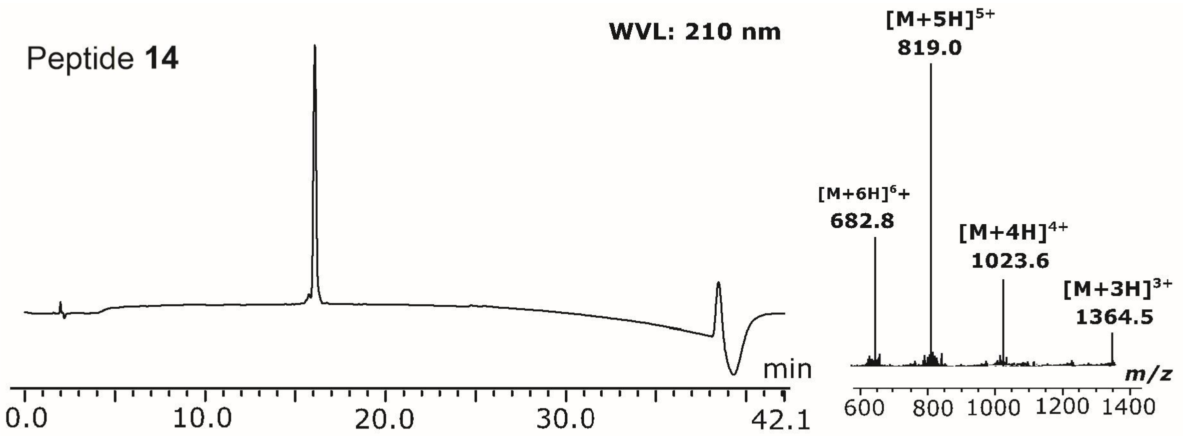

Figure 29. LC-MS chromatogram of purified peptide **14**. Analysed using a linear gradient of 5%B to 75%B over 35 min, *ca* 2%B/min.

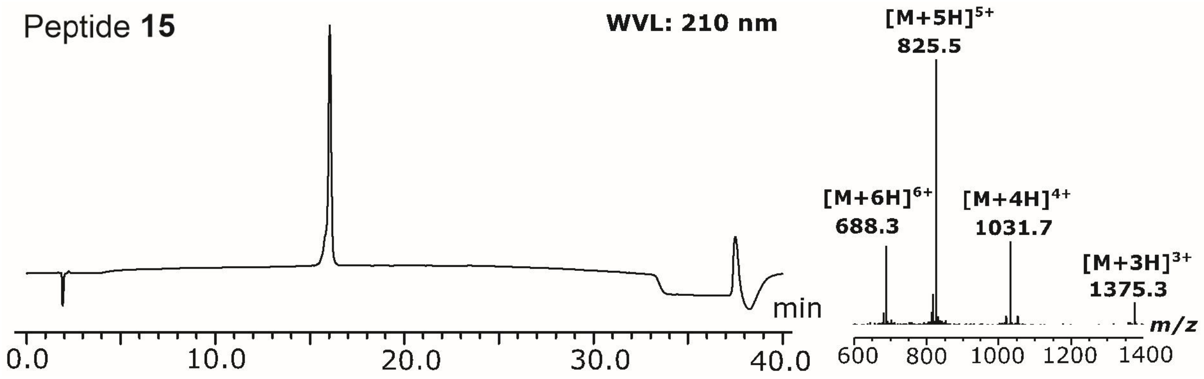

Figure 30. LC-MS chromatogram of purified peptide **15**. Analysed using a linear gradient of 5%B to 65%B over 30 min, *ca* 2%B/min.

4.3.2 Synthesis of polypeptides S21 and S22

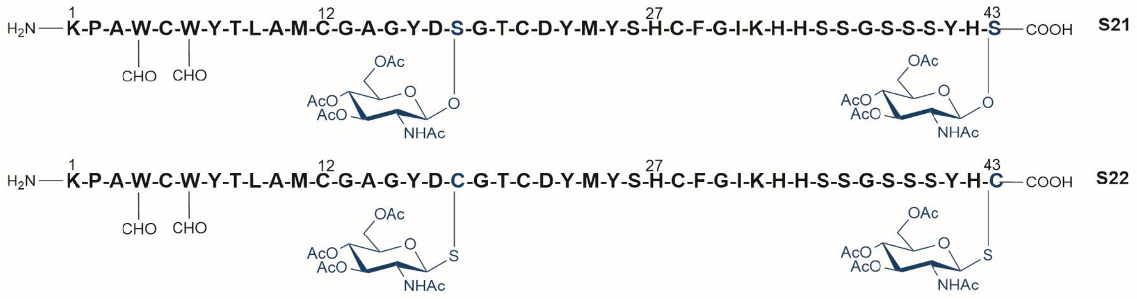

NCL reactions of peptides **14** (19.9 mg, 4.87 µmol) and **15** (20.1 mg, 4.87 µmol) with peptide **6** (10.5 mg, 4.87 µmol) were performed as described previously in the synthesis of peptide **7** using the ligation buffer (6 M GnHCl, 0.2 M Na_2HPO_4, 20 mM TCEP, and 100 mM MPAA) at a final peptide concentration of 1 Mm (Section 4.1.5). Purification by RP-HPLC afforded **S21**, H_2N-^{1}Lys-^{18}Ser(β-GlcNAc(OAc)$_3$)-^{43}Ser(β-GlcNAc(OAc)$_3$)-COOH, and **S22**, H_2N-^{1}Lys-^{18}Cys(β-GlcNAc(OAc)$_3$)-^{43}Cys(β-GlcNAc(OAc)$_3$)-COOH as amorphous solids.

Glycopeptide **S21** (10.7 mg, 40% yield, 97% purity); R_t 19.4 min; *m/z* (ESI-MS) 1375.3 ([M+4H]$^{4+}$ calculated: 1375.3). (Analysed using a linear gradient of 5%B to 75%B over 35 min, *ca* 2%B/min).

Glycopeptide **S22** (11.9 mg, 44% yield, 99% purity); R_t 19.2 min; *m/z* (ESI-MS) 1383.4 ([M+4H]$^{4+}$ calculated: 1383.3). (Analysed using a linear gradient of 5%B to 65%B over 30 min, *ca* 2%B/min).

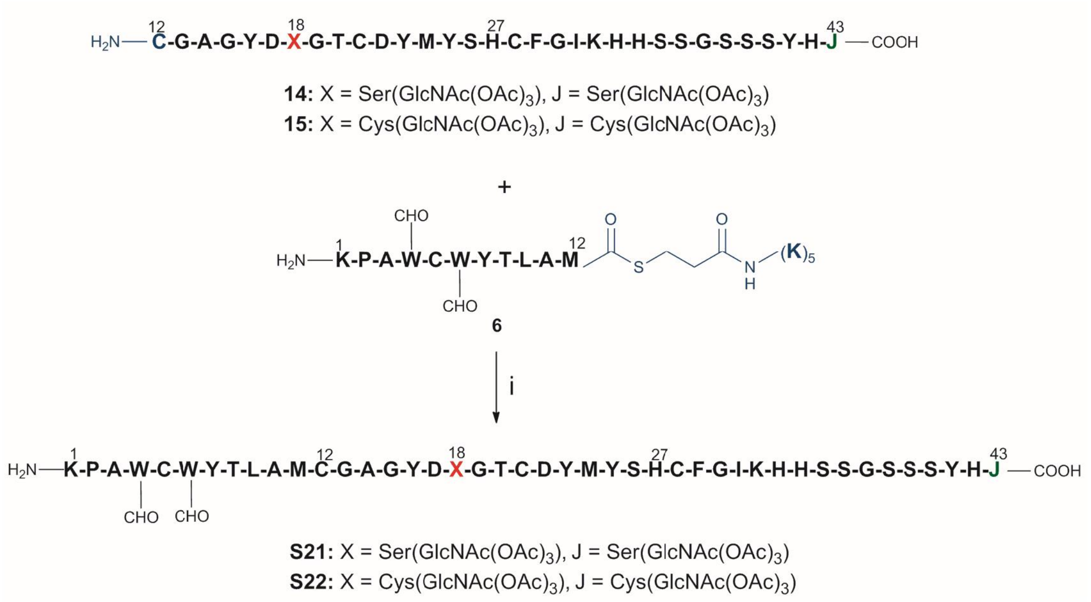

Scheme 11. Synthesis of glycopeptides **S21** and **S22**. *Reagents and conditions*: (i) 6 M Gn.HCl, 0.2 M Na_2HPO_4, 100 mM MPAA, 20 mM TCEP, pH 6.8, r.t, 4 h.

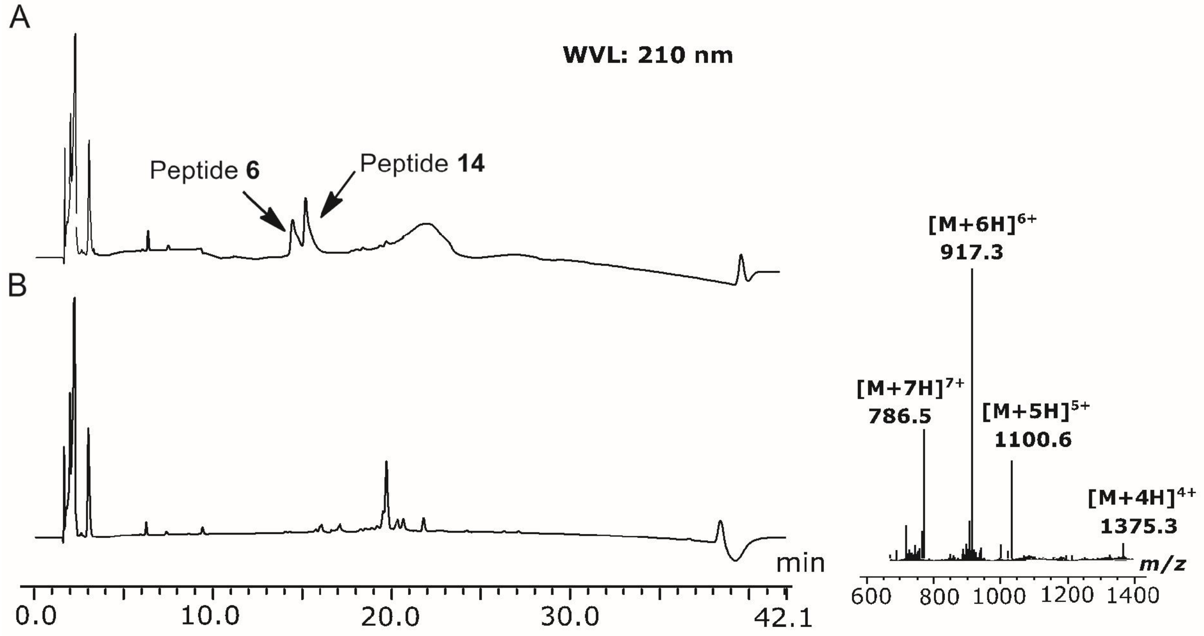

Figure 31. LC-MS chromatograms for synthesis of glycopeptide **S21**. (A) NCL of peptides **6** and **14** at t=0 min; (B) Generation of the desired glycopeptide at t = 4 h. Analysed by Vydac TP Diphenyl column (4.6 x 250 mm; 5 µm), gradient 5-75% B over 35 mins, 1.0 mL/min.

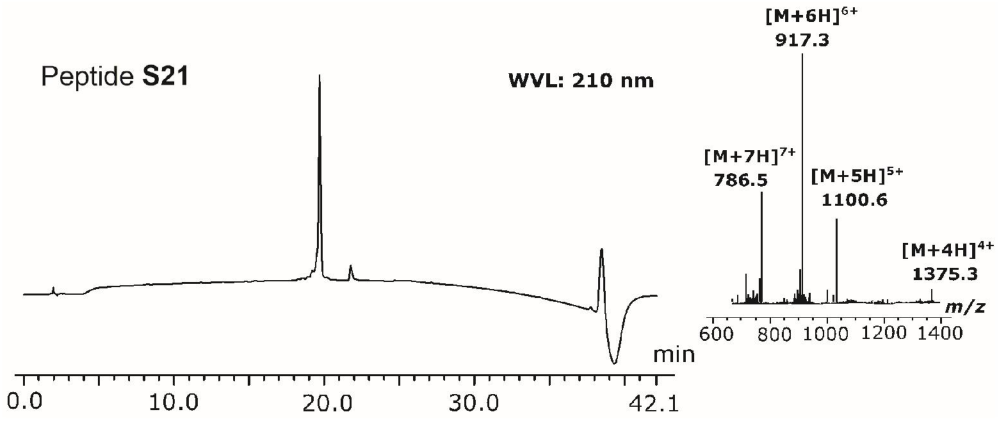

Figure 32. LC-MS chromatogram of purified peptide fragment **S21**. Analysed using a linear gradient of 5%B to 75%B over 35 min, *ca* 2%B/min.

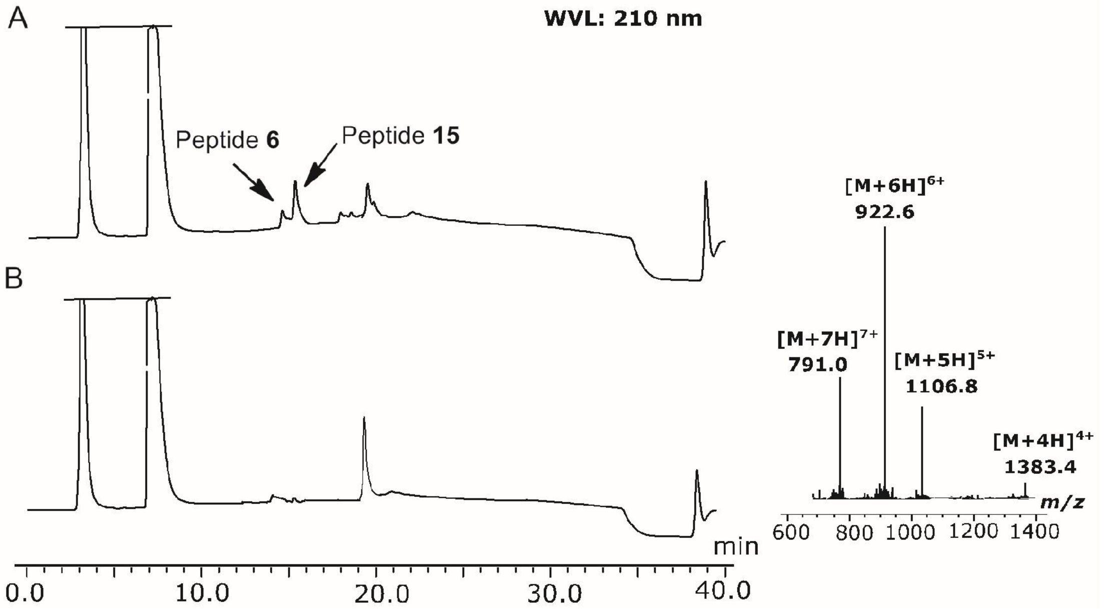

Figure 33. LC-MS chromatograms for synthesis of glycopeptide **S22**. (A) NCL of peptides **6** and **15** at t=0 min; (B) Generation of the desired glycopeptide at t = 4 h. Analysed by Grace Vydac TP Diphenyl column (4.6 x 250 mm; 5 µm); gradient 5-65% B over 30 mins, 1.0 mL/min.

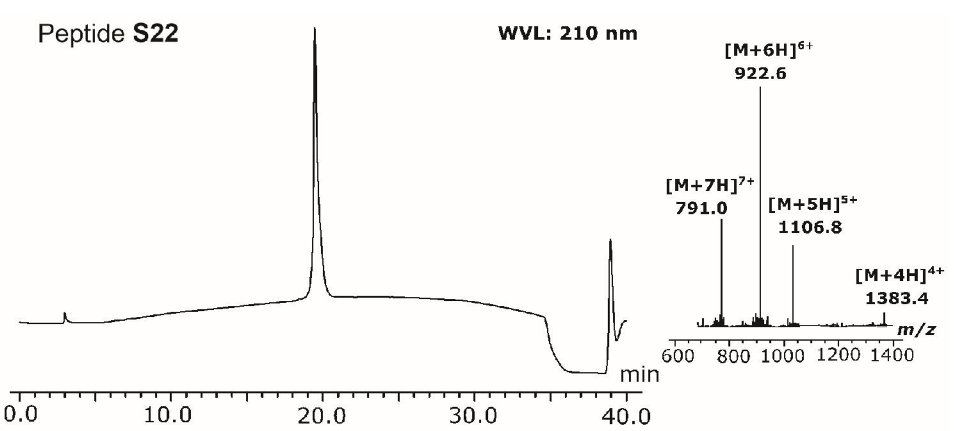

Figure 34. LC-MS chromatogram of purified peptide fragment **S22**. Analysed using a linear gradient of 5%B to 65%B over 30 min, *ca* 2%B/min.

4.3.3 Synthesis of polypeptides S23 and S24

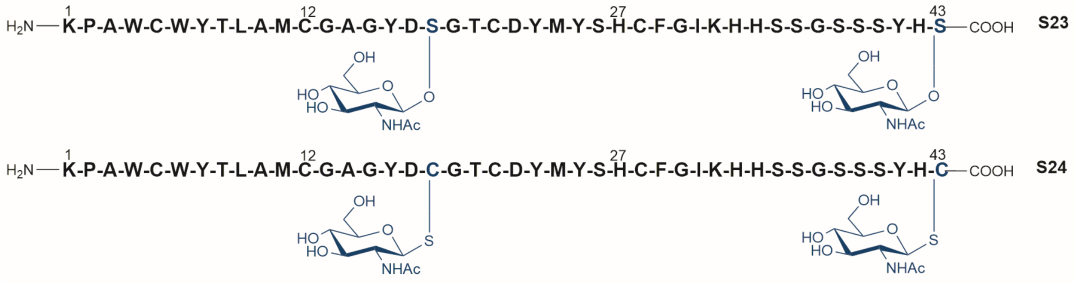

Glycopeptides **S21** (10.7 mg, 1.95 µmol) or **S22** (11.9 mg, 2.15 µmol) were subjected to global deprotection for simultaneous cleavage of the formyl groups on the side-chain of Trp and acetyl group on sugar moiety using the same conditions described previously in the synthesis of glycopeptide **8** (Section 4.1.6). Purification by RP-HPLC afforded **S23** H_2N-^{1}Lys-^{18}Ser(β-GlcNAc)-^{43}Ser(β-GlcNAc)-COOH, and **S24**, H_2N-^{1}Lys-^{18}Cys(β-GlcNAc)-^{43}Cys(β-GlcNAc)-COOH as amorphous solids.

Glycopeptide **S23** (3.14 mg, 31% yield, 98% purity); R_t 17.8 min; *m/z* (ESI-MS) 1298.1 ([M+4H]$^{4+}$ calculated: 1298.6). (Analysed using a linear gradient of 5%B to 75%B over 35 min, *ca* 2%B/min).

Glycopeptide **S24** (5.84 mg, 52% yield, 98% purity); R_t 18.2 min; *m/z* (ESI-MS) 1306.6 ([M+4H]$^{4+}$ calculated: 1306.6). (Analysed using a linear gradient of 5%B to 75%B over 35 min, *ca* 2%B/min).

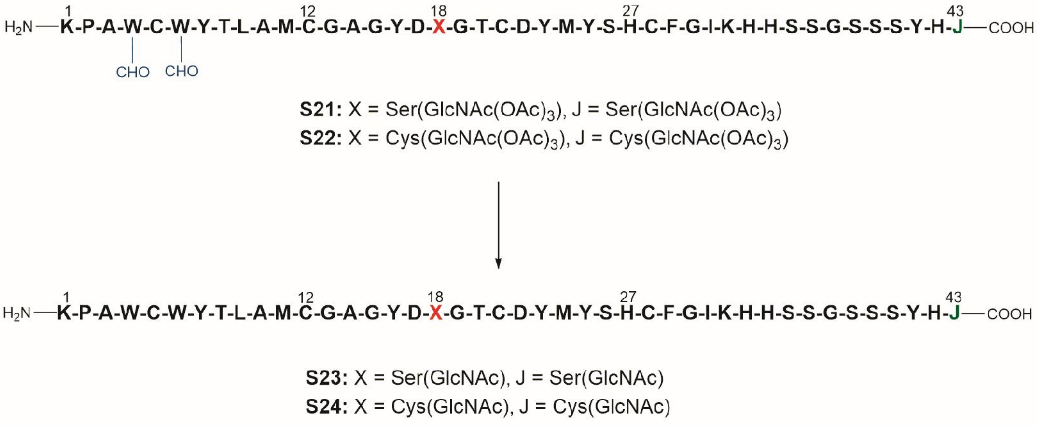

Scheme 12. Deprotection of acetyl and formyl groups to yield the glycopeptides **S23** and **S24**. *Reagents and conditions*: 6 M Gn.HCl, 1 M HEPES, hydrazine, 2-mercaptoethanol, 0 ^{o}C, 30 min.

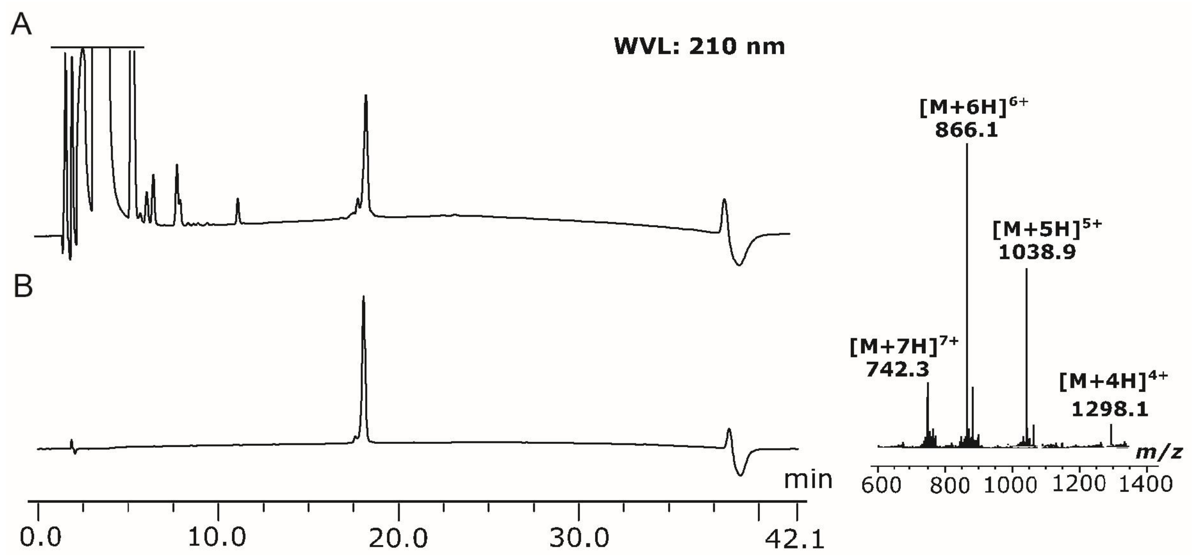

Figure 35. LC-MS chromatograms for the synthesis of glycopeptide **S23**. (A) The deprotection reaction after 30 min; (B) The purified glycopeptide **S23**. Analysed by XTerra® MS C_{18} column (4.6 x 150 mm; 5 µm), gradient 5-75% B over 35 mins, 1.0 mL/min.

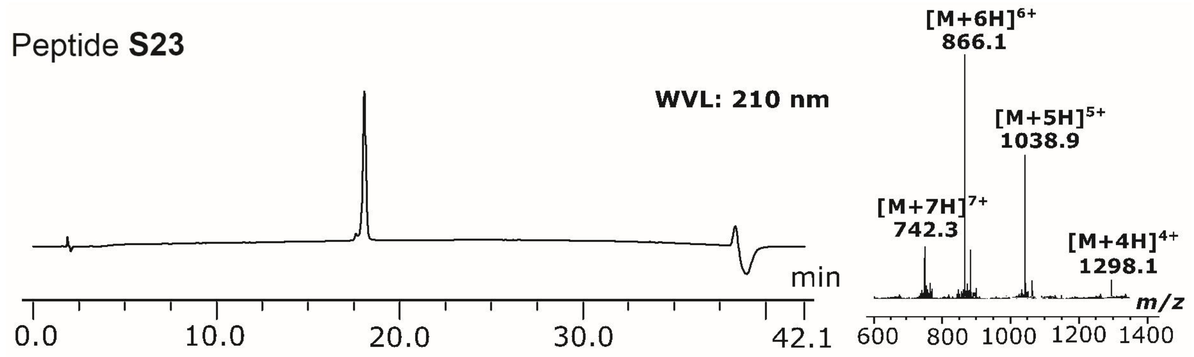

Figure 36. LC-MS chromatogram of purified peptide fragment **S23**. Analysed using a linear gradient of 5%B to 75%B over 35 min, *ca* 2%B/min.

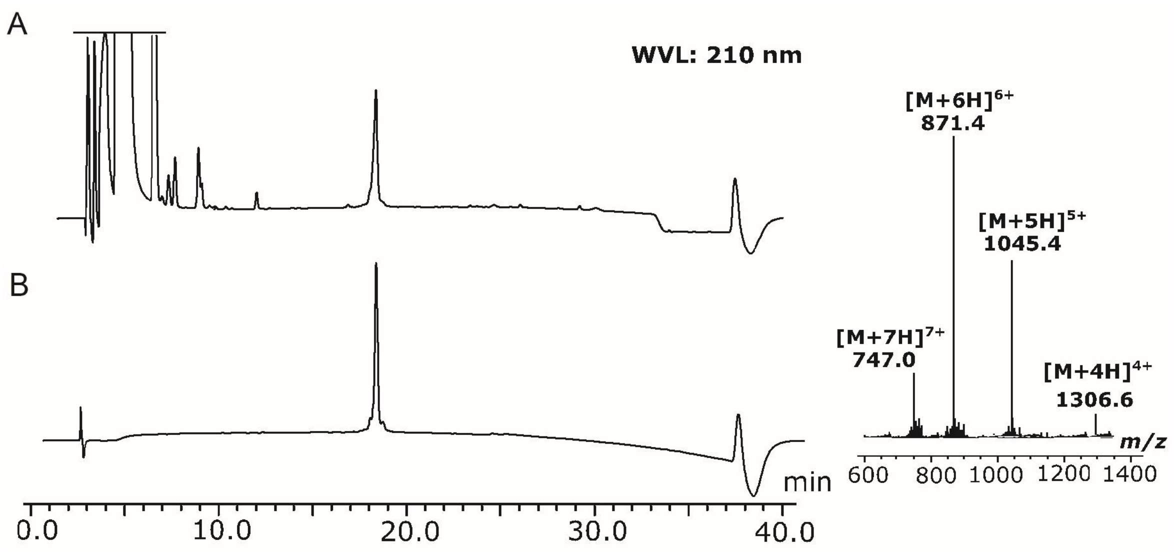

Figure 37. LC-MS chromatograms for the synthesis of glycopeptide **S24**. (A) The deprotection reaction after 30 min; (B) The purified glycopeptide **S24**. Analysed by XTerra® MS C_{18} column (4.6 x 150 mm; 5 µm), gradient 5-75% B over 35 mins, 1.0 mL/min.

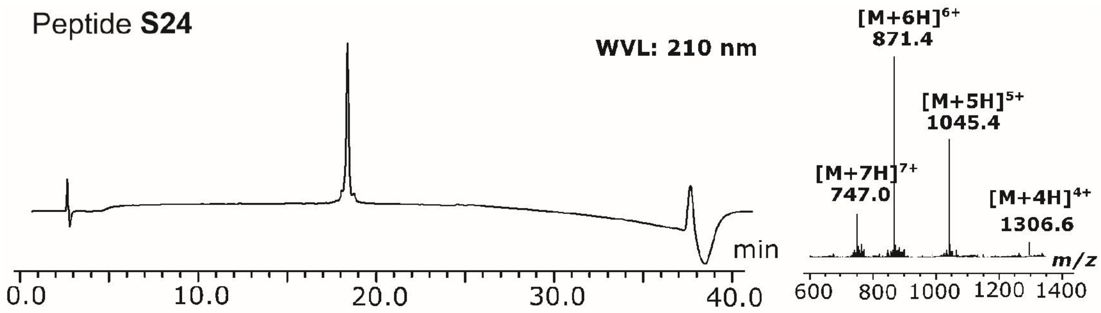

Figure 38. LC-MS chromatogram of purified peptide fragment **S24**. Analysed using a linear gradient of 5%B to 75%B over 35 min, *ca* 2%B/min.

4.3.4 Synthesis of 'Glyco-mutant' 10 and 11

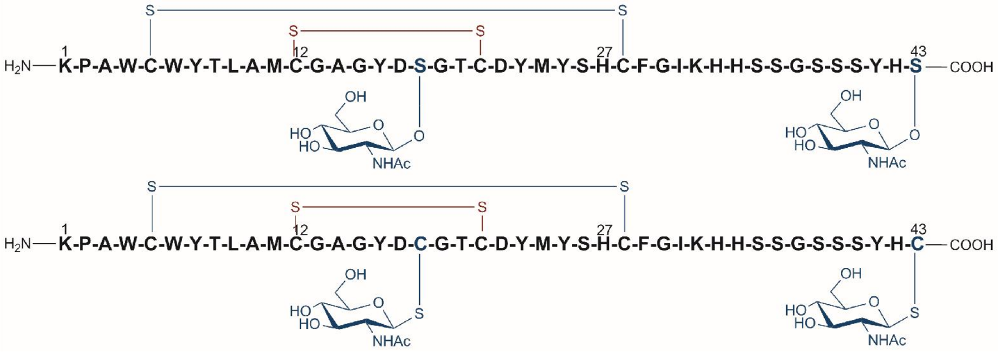

10

11

Glycopeptides **S23** (3.14 mg, 0.60 µmol) or **S24** (5.84 mg, 1.12 µmol) were folded using the same conditions described previously in the synthesis of native GccF **1** (Section 4.1.7). Purification by RP-HPLC afforded 'glyco-mutant' **10** and **11** as amorphous solids.

'glyco-mutant' **10** (0.9 mg, 29% yield, 99% purity); R_t 17.5 min; *m/z* (HRESI-MS) 1037.6191 ($[M+5H]^{5+}$ requires for $C_{226}H_{316}N_{57}O_{73}S_6$: 1038.2672). (Analysed using a linear gradient of 5%B to 75%B over 35 min, *ca* 2%B/min).

'glyco-mutant' **11** (1.8 mg, 31% yield, 99% purity); R_t 17.4 min; *m/z* (HRESI-MS) 1044.0154 ($[M+5H]^{5+}$ requires for $C_{226}H_{316}N_{57}O_{71}S_8$: 1044.6594). (Analysed using a linear gradient of 5%B to 75%B over 35 min, *ca* 2%B/min).

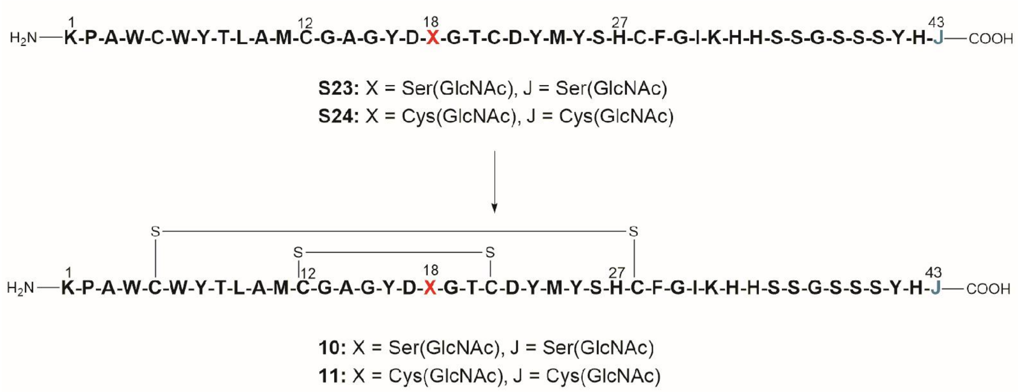

Scheme 13. The final oxidative folding of **S23** and **S24** to synthesise 'glyco-mutant' of GccF **10** and **11**, respectively. *Reagents and conditions*: 1.5 M Gn·HCl, 50 mM Na_2HPO_4, 2 mM cysteine, 0.25 mM cystine, 0.025 mM EDTA, pH 8.2, 0.25 mM peptide, 4 ^oC, 16h.

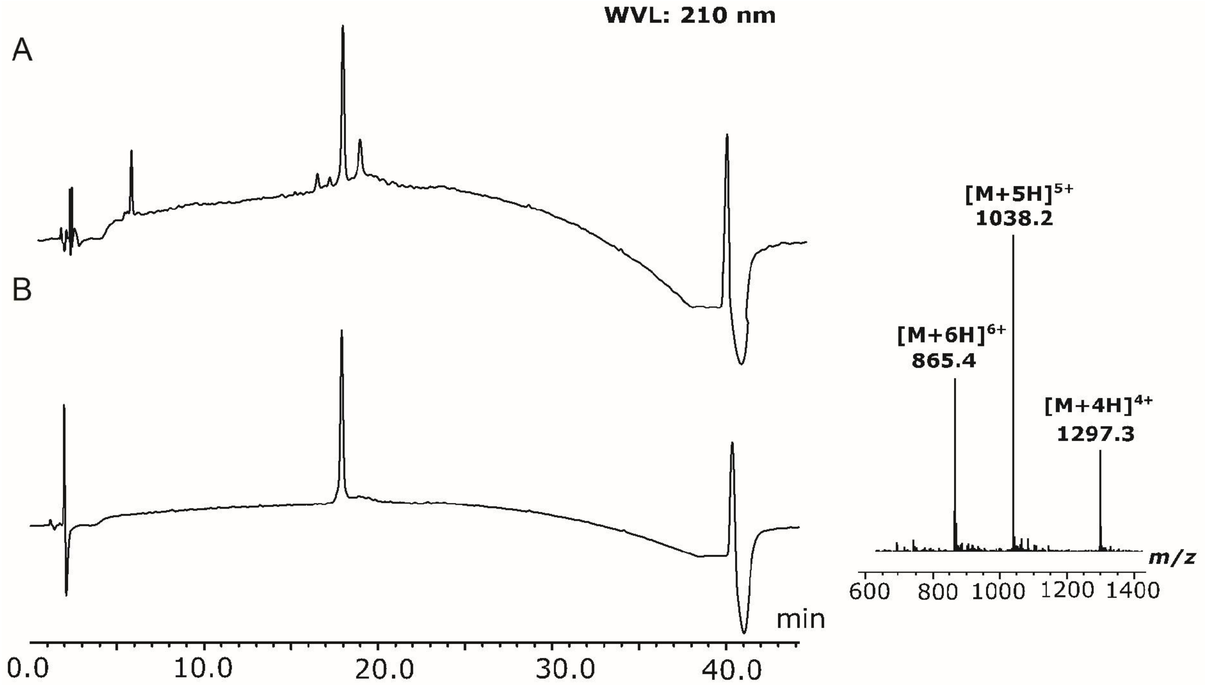

Figure 39. LC-MS chromatograms for the folding reaction of GccF analogue **10**. (A) crude profile of the overnight folding reaction; (B) the purified GccF analogue **10**. Analysed by XTerra® MS C_{18} column (4.6 x 150 mm; 5 µm), gradient 5-75% B over 35 mins, 1.0 mL/min.

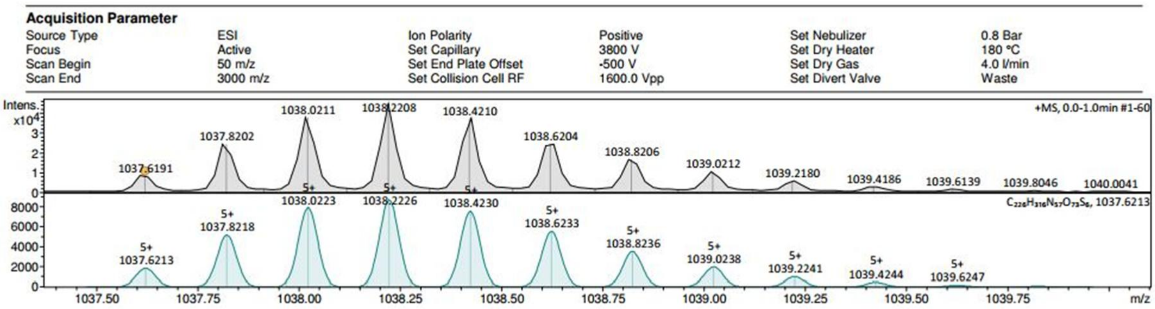

Meas. m/z	#	Ion Formula	m/z	err [ppm]	mSigma	# Sigma	Score	rdb	e⁻ Conf	N-Rule
1037.6191	1	C226H316N57O73S6	1037.6213	2.1	556.1	1	-1.#J	97.5	even	ok

Figure 40. RP-HPLC and HRESI-MS traces of peptide **10**.

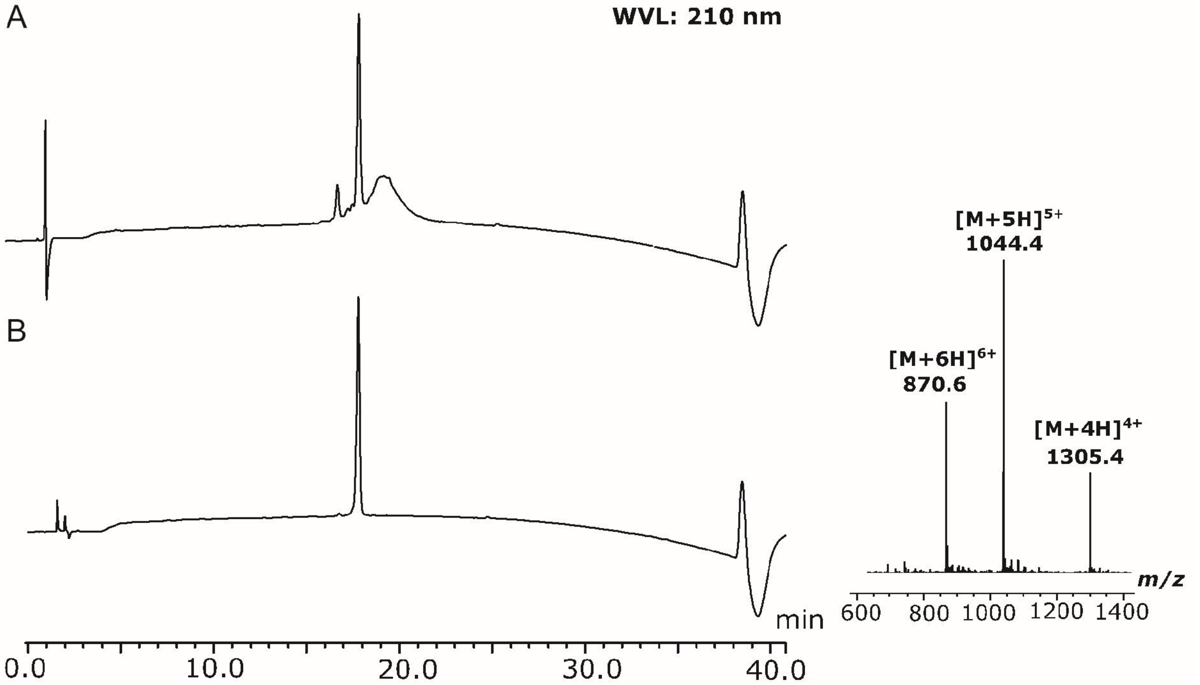

Figure 41. LC-MS chromatograms for the folding reaction of GccF analogue **11**. (A) crude profile of the overnight folding reaction; (B) the purified GccF analogue **11**. Analysed by XTerra® MS C_{18} column (4.6 x 150 mm; 5 µm), gradient 5-75% B over 35 mins, 1.0 mL/min.

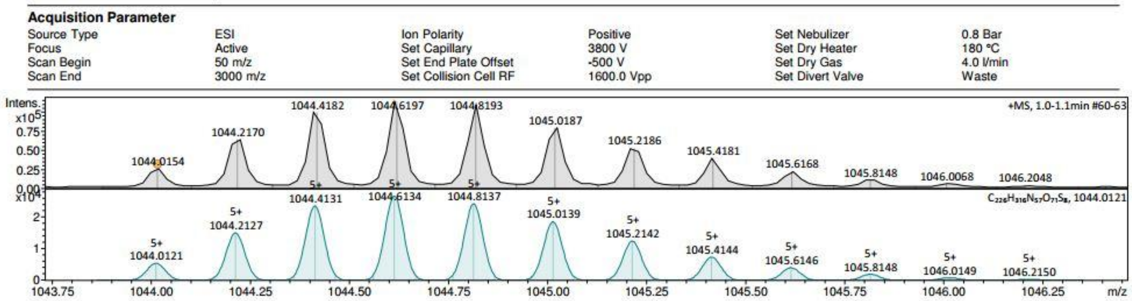

Meas. m/z	#	Ion Formula	m/z	err [ppm]	mSigma	# Sigma	Score	rdb	e⁻ Conf	N-Rule
1044.0154	1	C226H316N57O71S8	1044.0121	-3.1	570.8	1	-1.#J	97.5	even	ok

Figure 42. RP-HPLC and HRESI-MS traces of peptide **11**.

5 Circular Dichroism (CD) Spectroscopy

All CD spectra were recorded using a Chirascan CD spectrometer (Applied Photophysics Ltd., UK) at 20 °C with a cuvette of 0.1 mm path length (106-QS, Hellma Analytics, Müllheim, Germany) in the range from 180 and 260 nm at 0.5 nm intervals with a time-to-point of 0.5 s. Each CD spectrum measurement was normalised to 1 mg mL^{-1} concentration. The Spectra the average of ten scans obtained with a 1 nm optical bandwidth. The baseline scans were collected with the solvent alone (milliQ Water), averaged as above, and then subtracted from the sample scans. Data are expressed as mean residue ellipticities [Θ] in (deg cm^2 $dmol^{-1}$), and were calculated using Pro-Data Viewer (version 4.1.1, Applied Photophysics Ltd., UK).

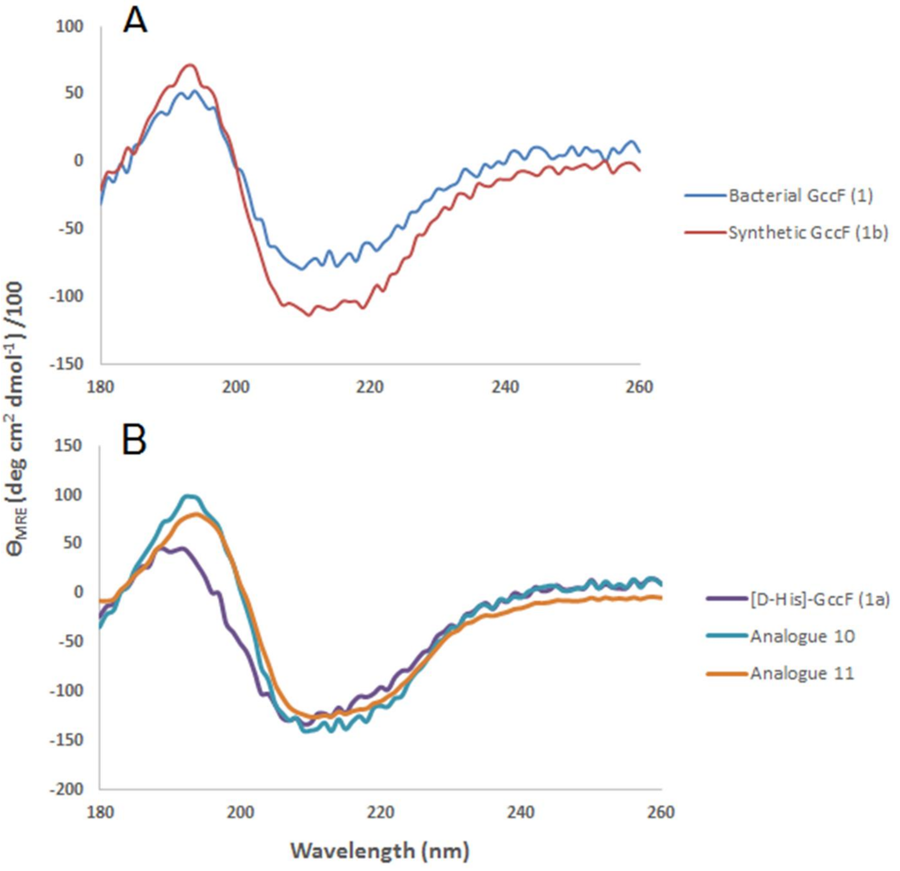

Figure 43. Circular dichroism spectroscopy data acquired at 1 mg mL^{-1} peptide in MilliQ Water of (A) Natural GccF and the synthetic derivative **1b**; (B) GccF analogues **1a**, **10** and **11**.

6 Antibacterial Assay

The glycopeptides were assayed for activity against *Lactobacillus plantarum* strain 8014, using a 96-well plate reader. Serial dilutions of the compound to be tested were prepared in MRS broth (Merck) and was added, in triplicate, to a 96-well plate containing an equal volume of *L. plantarum* 8014 at optical density 0.05. Additional positive controls (cells with no treatment) and negative controls (MRS media with no cells) were included, also in triplicate. Cells were grown in a Multiskan GO plate reader (Thermo Scientific) for 12-15 h, at 30 °C, and absorbance measured at 600 nm every 15 min. The growth of cells in each peptide concentration was modelled against the internal positive control, and the percentage inhibition derived at each time point as the difference between the optical density of the positive control and the optical density of cells within a given peptide concentration. Taking the maximum point from plots of percentage inhibition versus time for each peptide concentration allowed a polynomial fit to be applied, and the concentration required to reach 50% inhibition derived. Statistical error was taken as the maximum variance across the triplicates of each concentration in a given plate reader run.

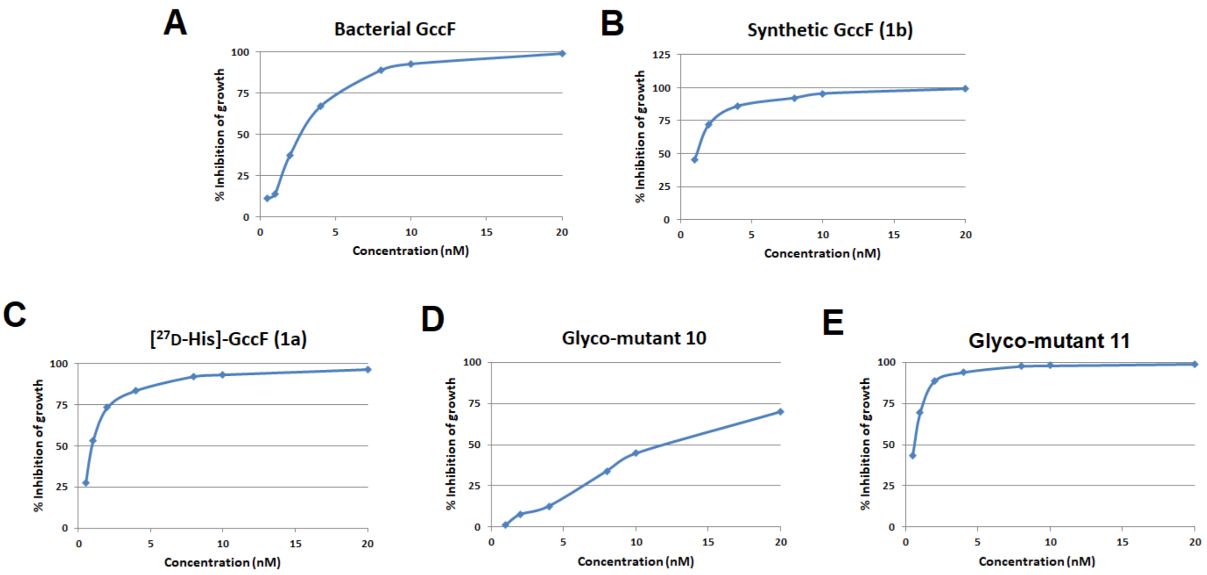

Figure 44. The results from testing the bacterially-isolated GccF, synthetic GccF (**1b**) and analogues **1a**, **10**, and **11**, plotted as percentage inhibition of *Lactobacillus plantarum* ATCC 8014 growth vs concentration of the glycopeptide.

7 References

1. P. W. Harris, D. J. Lee and M. A. Brimble, *J. Pept. Sci.*, 2012, **18**, 549-555.
2. Y. Chen, C. T. Mant and R. S. Hodges, *J. Chromatogr.*, 2007, **1140**, 112-120.
3. M. A. Brimble, P. J. Edwards, P. W. Harris, G. E. Norris, M. L. Patchett, T. H. Wright, S. H. Yang and S. E. Carley, *Chem. Eur. J.*, 2015, **21**, 3556-3561.
4. D. Horton, *Methods Carbohydr. Chem.*, 1972, **6**, 282-285.
5. A. C. Cunha, L. O. Pereira, R. O. de Souza, M. C. B. De Souza and V. F. Ferreira, *Nucleosides Nucleotides Nucleic Acids*, 2001, **20**, 1555-1569.
6. A. Kuhn and H. Kunz, *Angew. Chem. Int. Ed.*, 2007, **46**, 454-458.
7. M. Ginisty, C. Gravier-Pelletier and Y. Le Merrer, *Tetrahedron: Asymmetry*, 2006, **17**, 142-150.
8. S. Nakabayashi, C. D. Warren and R. W. Jeanloz, *Carbohydr. Res.*, 1986, **150**, c7-c10.
9. V. Wittmann and D. Lennartz, *Eur. J. Org. Chem.*, 2002, **2002**, 1363-1367.
10. I. Carvalho, S. L. Scheuerl, K. P. Ravindranathan Kartha and R. A. Field, *Carbohydr. Res.*, 2003, **338**, 1039-1043.
11. B. Paul and W. Korytnyk, *Carbohydr. Res.*, 1984, **126**, 27-43.
12. Y. S. Hsieh, B. L. Wilkinson, M. R. O'Connell, J. P. Mackay, J. M. Matthews and R. J. Payne, *Org. Lett.*, 2012, **14**, 1910-1913.
13. E. Kaiser, R. L. Colescott, C. D. Bossinger and P. I. Cook, *Anal. Biochem.*, 1970, **34**, 595-598.
14. M. Schnolzer, P. Alewood, A. Jones, D. Alewood and S. B. H. Kent, *Int. J. Pept. Protein Res.*, 1992, **40**, 180-193.
15. T. M. Hackeng, J. H. Griffin and P. E. Dawson, *Proc. Natl. Acad. Sci.*, 1999, **96**, 10068-10073.
